# Targeting Histone Modifications in Breast Cancer: A Precise Weapon on the Way

**DOI:** 10.3389/fcell.2021.736935

**Published:** 2021-09-14

**Authors:** Wei Li, Hao Wu, Shiyao Sui, Qin Wang, Shouping Xu, Da Pang

**Affiliations:** ^1^Harbin Medical University Third Hospital: Tumor Hospital of Harbin Medical University, Harbin, China; ^2^Heilongjiang Academy of Medical Sciences, Harbin, China

**Keywords:** histone modification, breast cancer, acetylation, methylation, epi-drugs

## Abstract

Histone modifications (HMs) contribute to maintaining genomic stability, transcription, DNA repair, and modulating chromatin in cancer cells. Furthermore, HMs are dynamic and reversible processes that involve interactions between numerous enzymes and molecular components. Aberrant HMs are strongly associated with tumorigenesis and progression of breast cancer (BC), although the specific mechanisms are not completely understood. Moreover, there is no comprehensive overview of abnormal HMs in BC, and BC therapies that target HMs are still in their infancy. Therefore, this review summarizes the existing evidence regarding HMs that are involved in BC and the potential mechanisms that are related to aberrant HMs. Moreover, this review examines the currently available agents and approved drugs that have been tested in pre-clinical and clinical studies to evaluate their effects on HMs. Finally, this review covers the barriers to the clinical application of therapies that target HMs, and possible strategies that could help overcome these barriers and accelerate the use of these therapies to cure patients.

## Introduction

**FIGURE 1:**
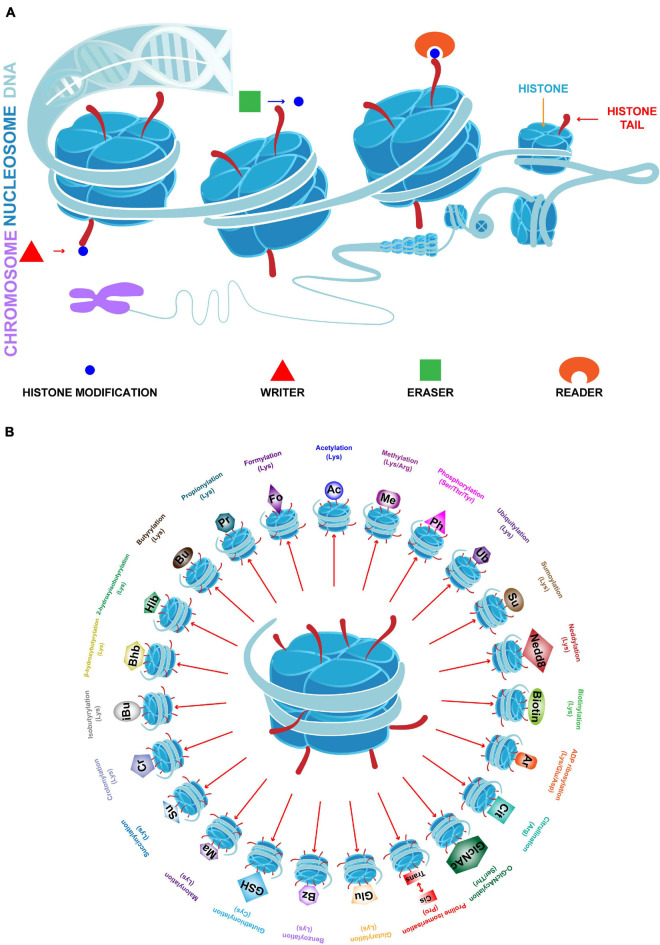
Chromatin structure and 23 classes of HMs. **(A)** In eukaryotic cells, chromatin is the complex formed by DNA and histones. The basic functional unit of chromatin is the nucleosome which contains a histone octamer (H2A, H2B, H3, and H4) which is wrapped by DNA. Histone tails undergo numerous posttranslational modifications, which are deposited by writers, removed by erasers and read by readers, and may either loosen or tighten DNA-histone binding which active or silence transcription. **(B)** Specific HMs and modified amino acids are indicated.

Breast cancer (BC) has the highest incidence and mortality rate among cancer cases in women (Sung et al., 2021). The development and progression of BC depends on complicated genetic and epigenetic changes. These changes include histone modifications (HMs) that regulate gene expression without altering DNA sequence, which may contribute to the tumorigenesis and progression of BC (Egger et al., 2004; Huang et al., 2011). Chromatin is a macromolecular complex of DNA and proteins that act as a scaffold in assembling the entire genome into nucleosomes, the basic functional unit of chromatin. The core component of the nucleosome is an octamer consisting of four pairs of histones (H3, H4, H2A, and H2B). Histones are wrapped around a segment of 147 base pairs of DNA. The highly basic histone amino (N)-terminal tails protrude from each of the eight histones that are enriched with large amount of covalent posttranslational modifications (PTMs), which are deposited by “writer” modules and removed by “eraser” modules in a histone and sequence-specific manner; in addition, PTMs serve as scaffolds for “reader” modules (Figure 1A; Strahl and Allis, 2000). While different aspects of HMs are relatively well understood, a deeper understanding of these processes is needed to clarify the roles of HMs and their enzymatic mechanisms in BC. Although at least 23 classes of HMs are known, only a few have been shown to be related to BC. A summary of the known specific targets for each HM class is shown in Figure 1B. This review primarily focuses on histone acetylation and methylation, which are the most widely studied classes. The aberrant regulation of these processes alters the balance of gene expression in BC, and leads to abnormal cellular proliferation, invasion, metastasis, and drug resistance (Jovanovic et al., 2010; Karsli-Ceppioglu et al., 2014).

We summarize the existing evidence regarding HMs and their influence on BC progression. Furthermore, we review research done on enzymes involved in HMs and the potential regulation of these enzymes. Finally, we focus on existing and emerging drugs that target HMs and related enzymes, potential barriers to their clinical application, and possible strategies to help overcome those barriers.

## HMs in BC

There is evidence that HMs play vital roles in the tumorigenesis and progression of BC, and changes in global patterns of HMs can produce different effects. For example, seven HM markers (H3K9ac, H3K18ac, H4K12ac, H4K16ac, H3K4me2, H4K20me3, and H4R3me2) were evaluated in 880 BC specimens, and expressions of all seven markers were negatively correlated with tumor grade. Moreover, high levels of H4R3me2 and H3K9ac were detected at lower lymph node stages, and H4R3me2, H3K9ac, and H4K16ac are lower in large tumor sizes. More importantly, low or missing H4K16ac was detected in most specimens, implying that this change represented an early event in BC, which may be used for BC diagnosis. In addition, high levels of these markers in various BC subtypes were associated with a better prognosis, and were almost exclusively detected in luminal BC. In contrast, low levels of these markers were observed in triple-negative breast cancer (TNBC) and HER2-positive BC, which have a poorer prognosis (Elsheikh et al., 2009). There is a global pattern alteration of HMs during breast malignant transformation. For instance, in 58 breast samples, acetylated histone H4, H4K12ac, acetylated tubulin, HDAC1, HDAC2, and HDAC6 were lower in ductal carcinoma *in situ* (DCIS) and invasive ductal carcinoma (IDC) than in normal mammary epithelium (Suzuki et al., 2009). In BC, phospho-histone H3 (PPH3) is a proliferative marker that is more robust to predict prognosis than Ki67, and associated with a poor overall survival (Skaland et al., 2007, 2009; Gerring et al., 2015; Kim et al., 2017). Upon DNA double-strand breaks, activated PI3K family members, ATM and ATR catalyze the phosphorylation of histone H2AX, which is known as γH2AX. As a consequence, γH2AX which is identified to be a biomarker for DNA damage and repair, triggers the cell cycle check and double-strand repair (Lord and Ashworth, 2009). In BC, γH2AX is associated with lower estrogen receptor (ER) and progesterone receptor (PR) expression and poor clinicopathological characteristics, including larger tumor size, higher grade, and more lymph nodes infiltration (Varvara et al., 2019). In TNBC, γH2AX is correlated with shorter telomeres and poorer prognosis (Nagelkerke et al., 2011, 2015). In addition, various HMs contribute to the activation of oncogenes or inhibition of tumor suppressor genes (TSGs), which lead to sustained proliferative signaling, acceleration of cell cycle, angiogenesis, invasion and metastasis, DNA damage, resistance to death, reprogramming of energy metabolism, and evasion of immune destruction. In summary, HMs have gained a significant position as biomarkers of BC diagnosis and prognosis. Research on the underlying mechanism of HMs also provide hope for development of specific inhibitors. The specific HMs and their distinct roles in BC will be discussed in the following sections.

## Histone Acetylation

Histone acetylation is characterized by addition of an acetyl group to the lysine residues of histone tails (Kouzarides, 2007). This modification alters the interaction between the tails and negatively charged DNA by neutralizing the positive charge on the lysine residue, which, in turn, facilitates chromatin opening and promotes transcription. This has been confirmed to occur in promoters, enhancers, as well as in the whole transcribed region (Heintzman et al., 2007; Wang Z. et al., 2008). Acetylation of the histone lysine residues is a dynamic and reversible process that is regulated by the competitive actions of two enzyme types: histone acetyltransferases (HATs or histone acetylation “writers”) and histone deacetylases (HDACs or histone acetylation “erasers”) (Ropero and Esteller, 2007; Sheikh and Akhtar, 2019). Moreover, acetylated lysine also serves as a target for the binding of numerous proteins (readers) which recognize this modification (Taverna et al., 2007; Josling et al., 2012).

### Histone Acetylation Writers

**FIGURE 2:**
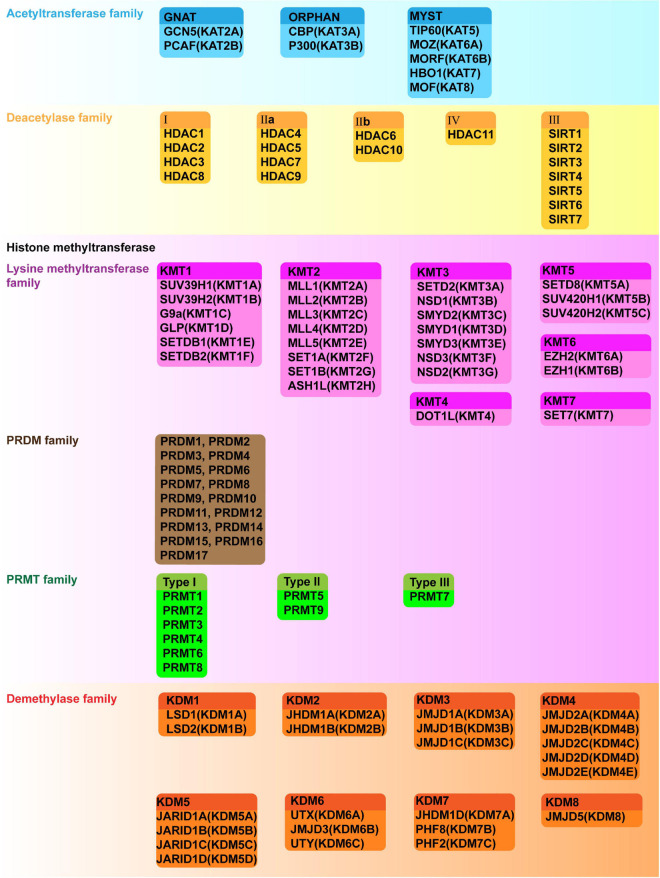
Classification, formal names and aliases of HATs, HDACs, HMTs and KDMs.

**FIGURE 3:**
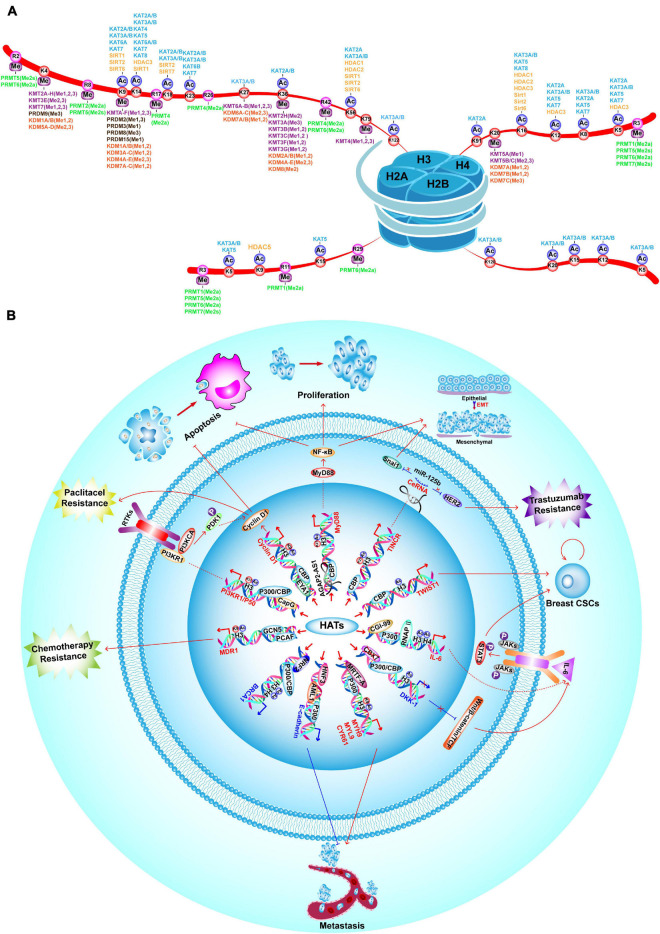
Landscape of HM modifiers with their substrates, and the functions of HATs in BC. **(A)** Landscape of histone acetylation and methylation writers, erasers and their catalyzed substrates. **(B)** HATs promote BC proliferation, invasion and metastasis, drug-resistance, EMT, CSC properties and inhibit apoptosis by enhancing transcription of oncogenes, such as *TINCR*, *MyD88*, *Cyclin D1*, *PI3KR1/P50*, *MDR1*, *MYH9*, *MYL9*, *CYR61*, *IL-6*, and *TWIST1*. On the contrary, P300/CBP inhibit BC tumorigenesis, metastasis and CSC properties by enhancing transcription of TSGs, such as *E-cadherin* and *BRCA1*.

Histone acetylation is catalyzed by a group of HATs. In humans, there are three major families of HATs (Figures 2, 3A): the GNAT family (HAT1, GCN5, and PCAF), the MYST family (TIP60, MOZ, MORF, HBO1, and MOF), and the ORPHAN family (P300/CBP) (Marmorstein and Zhou, 2014). Among these histone acetylation “writers,” the orphan family members (P300 and CBP) possess HAT domains, transcription factor binding domains, and bromodomains (BRDs), which allows them to serve as global acetyltransferases, transcriptional coactivators, and the readers of HMs (Ogryzko et al., 1996; Garcia-Carpizo et al., 2019). In addition, P300 and CBP have similar sequences and functions, such as combining with common viral and DNA binding transcription factors, for this reason, they are named as P300/CBP. Overexpression of P300 contributes to an increased risk of BC recurrence and reduced survival (Xiao et al., 2011). Furthermore, P300/CBP contribute to the transcription of oncogenes and TSGs, which promote or inhibit numerous BC-related processes, including proliferation (Wu et al., 2013; Dong et al., 2018; Chi et al., 2019), invasion and metastasis (He et al., 2015; Lin et al., 2017; Yu et al., 2017), epithelial-mesenchymal transition (EMT) (Manupati et al., 2019), development of cancer stem cells (CSCs) (Liang et al., 2015; Lin et al., 2017), apoptosis (Dong et al., 2018; Tomita et al., 2019), and drug resistance (Supplementary Table 1; Figure 3B; Toth et al., 2012; Dong et al., 2018; Manupati et al., 2019; Tomita et al., 2019). Furthermore, P300/CBP mediates alterations in histone acetylation landscape, promoting the relaxation of chromatin, and allowing the binding of transcriptional factors to activate transcription. For example, the binding of ER to estrogen response elements (EREs) is NF-κB dependent and is promoted by CBP-mediated changes in histone acetylation, thus potentiating TNF-dependent expression of the antiapoptotic gene *BIRC3* (Pradhan et al., 2012). Similarly, P300 can be activated by YB-1, changing the histone acetylation landscape to promote chromatin relaxation and allowing YB-1 to bind the promoter and transcriptionally regulate *BMI1*, which promotes a stem-like BC phenotype (Davies et al., 2014). In addition to acting alone, different writers collectively mediate HMs that modify the expression of a particular gene to promote or inhibit BC. For example, MLL1, MLL3, and P300/CBP are recruited to the promoter of a long non-coding RNA molecule (*HOTAIR*), which increases H3K4me3 and histone acetylation, activates transcription, suppresses apoptosis, and potentiates the progression of BC (Bhan et al., 2013).

### Histone Acetylation Erasers

**FIGURE 4:**
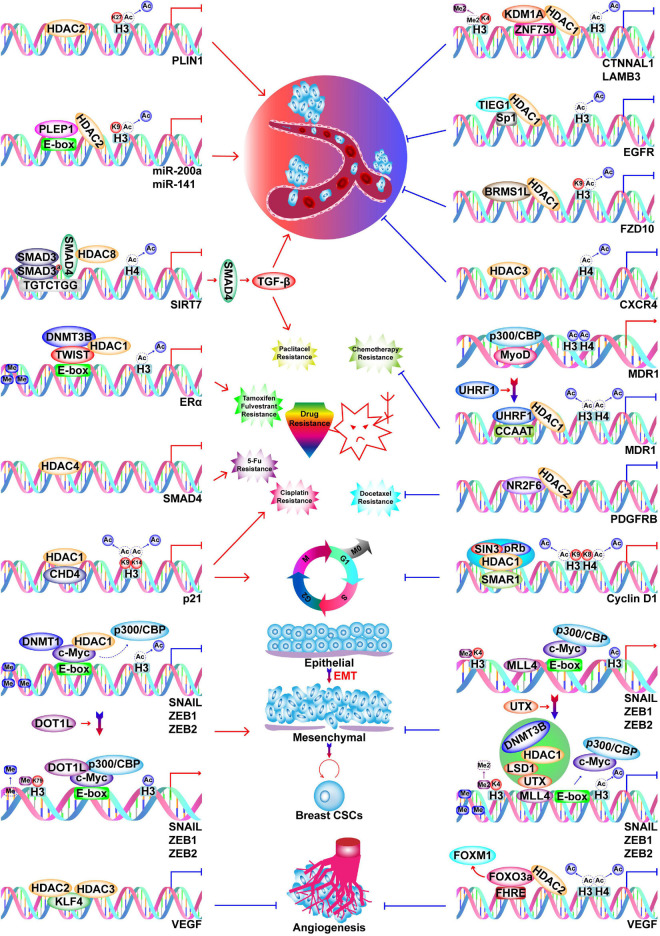
Functions of HDACs in BC. HDACs play dual roles in BC progression by inhibiting transcription of oncogenes and TSGs. HDACs promote BC metastasis by silencing *PLIN1*, *miR-200a*, *miR-141*, and *SIRT7*, while they inhibit metastasis by silencing *CTNNAL1*, *LAMB3*, *EGFR*, *FZD10*, and *CXCR4*. Silencing of *SIRT7* induces paclitaxel resistance via SIRT7/SMAD4/TGF-β pathway. Silencing of *ER*α, *SMAD4*, and *p21* by HDACs cause resistance to tamoxifen and fulvestrant, paclitaxel, 5-Fu, and cisplatin respectively. Silencing of *p21* by HDAC1 also causes enhanced cell cycle progression. HDAC2 silences *PDGFRB* to inhibit docetaxel resistance.

There are five classes of HDACs, which are listed in Figure 2. In BC, HDACs exert dual roles by epigenetically regulating cell cycle progression, proliferation (Liu R. et al., 2009; Hou et al., 2017; Yu et al., 2020), EMT, angiogenesis (Karadedou et al., 2012; Ray et al., 2013), metastasis (Kim et al., 2010; Jin et al., 2012; Gong et al., 2014; Roy et al., 2014; Cassandri et al., 2020; Lu C. et al., 2020; Tang et al., 2020), and drug resistance (Supplementary Table 1; Figure 4; Jin et al., 2010; Vesuna et al., 2012).

Transcription of ERα and the estradiol-mediated ER target genes is generally regulated by HDACs (Khurana et al., 2011; Vesuna et al., 2012; Nait Achour et al., 2014; Linares et al., 2019). For example, HDAC1 and DNMT3B are recruited by Twist to the ERα promoter, where they repress transcription (Vesuna et al., 2012). However, treatment of BC cells with HDAC inhibitors (HDACis) restores the expression of ERα (Kawai et al., 2003; Liu and Bagchi, 2004; Sharma et al., 2005, 2006). Therefore, epigenetically reactivated ER serves as a target of tamoxifen; specifically, tamoxifen-bound reactivated ER recruits the nucleosome remodeling and histone deacetylation (NuRD) complex and the nuclear receptor corepressor (NCoR)–HDAC3 complex to transcriptionally suppress estrogen-response genes (Liu and Bagchi, 2004; Sharma et al., 2006). However, caution is required when using HDACis because in addition to a suppression of lymph node tumor growth, pharmacological inhibition of HDAC11 enhances metastasis from the lymph node to distant sites (Leslie et al., 2019). Changes in HMs can also explain the malignant transformation of BC in response to a high-calorie diet or obesity. In this context, the elevated NAD^+^/NADH ratio leads to a decrease in binding of the transcriptional co-repressor C terminal-binding protein (CtBP) to the *BRCA1* promoter, which is accompanied by loss of HDAC1, increased histone acetylation, and thus, increased *BRCA1* transcription (Di et al., 2010). However, the functions of HDACs are not limited to transcriptional suppression since HDAC7 plays roles in transcriptional activation and repression, which depend on both the cell line and microenvironment (Caslini et al., 2019).

The class III HDACs consist of sirtuins (SIRT1–7), with SIRT1 predominantly located in the nucleus, although it can shuttle between the nucleus and cytoplasm (Tanno et al., 2007). In BC, SIRT1 plays dual roles by epigenetically silencing TSGs and oncogenes. For example, SIRT1 insufficiency reactivates abnormally silenced TSGs via increased H4K16 acetylation on various promoters, including genes that encode *SFRP1*, *E-cadherin*, and *GATA-5* (Pruitt et al., 2006). In contrast, SIRT1 causes silencing of *Survivin* via deacetylation of H3K9, which inhibits BC-related gene transcription, expression, and ultimately tumor growth (Wang R. H. et al., 2008). The precise role of SIRT1 is principally determined by the tumor subtype, because it can promote tumorigenesis in luminal molecular subtypes and inhibit carcinogenesis in TNBC (Rifaï et al., 2017, 2018). Furthermore, overexpression of SIRT2 in TNBC cells promotes histone H4 deacetylation at *ARRDC3* (a TSG), which contributes to an aggressive biological behavior (Soung et al., 2014). SIRT3-5 members are predominantly located in the mitochondria, where they modify various substrates involved in energy metabolism via deacetylation, ADP-ribosylation, or desuccinylation.

### Histone Acetylation Readers

The “reading” of histone acetylated lysine is performed by the BRD motif in reader proteins, which share high sequence homology, structural similarity, and play important roles in regulating gene expression (Fujisawa and Filippakopoulos, 2017). These proteins act as scaffolds, transcription factors, transcriptional co-regulators, HATs, histone methyltransferases (HMTs), helicases, and ATP-dependent chromatin-remodeling complexes. The various functions of BRD-containing proteins in BC are listed in Supplementary Table 2. Among these, the most studied group are the bromodomain and extra terminal domain (BET) family. The BRD4 member functions as a scaffold to facilitate the assembly of larger protein complexes, which leads to oncogene expression and BC tumorigenesis. The BRD4 protein has two BRDs that bind acetylated histone H4 and di-acetylated Twist, which create an activated Twist/BRD4/P-TEFb/RNA-PolII complex at the *WNT5A* promoter and enhancer regions. Activation of *WNT5A* causes tumorigenicity, enhanced invasion, and CSC-like properties (Shi et al., 2014). However, BRD4 also promotes homologous recombination (HR)-mediated DNA repair via enhanced transcription of related genes (*BRCA1* and *RAD51*). Pharmacological inhibition of BRD4 impairs the ability of TNBC cells to manage DNA damage after exposure to platinum salts, which leads to massive cell death and synthetic lethality when the platinum salts are combined with poly ADP ribose polymerase (PARP) inhibitors (PARPi) (Mio et al., 2019). Interestingly, the less abundant short isoform of BRD4 is oncogenic, whereas its long isoform suppresses BC cell proliferation and migration as well as BC tumor formation and metastasis (Wu et al., 2020). The oncogenic functions of BRD4 can be reversed via inhibitor treatment, which is discussed in section Histone acetylation reader inhibitors below.

## Histone Methylation

Histone methylation occurs on lysine and arginine residues, where it involves a more sophisticated set of modifications in contrast to acetylation. Lysine can be mono-, di-, or trimethylated, while arginine can be symmetrically or asymmetrically methylated (Barski et al., 2007). Histone methylation is a reversible process that is stringently regulated by various methyltransferase and demethylases. Some of these markers (H3K4, H3K36, and H3K79) are associated with transcriptional activation, whereas other markers (H3K9, H3K27, and H4K20) are associated with transcriptional repression (Jenuwein and Allis, 2001). The alterations in BC caused by writers and erasers of histone methylation are summarized in Supplementary Table 1.

### Histone Methylation Writers

Histone methyltransferases or “writers” are generally divided in three groups. The first group of methyltransferases have the SET domain [SET: Su(var)3-9, enhancer of zeste and trithorax] and consists of lysine methyltransferases (KMTs) with the exception of DOT1L (KMT4) (Dillon et al., 2005). The second group of methyltransferases have a non-SET domain and consists of DOT1L and the PRDM (PRD-BF1 and RIZ homology domain containing) protein family members, which have an N-terminal PR domain (Feng et al., 2002; Mzoughi et al., 2016). The third group of methyltransferases is the PRMT family (protein arginine methyltransferase), which shares a common methyltransferase domain (Kawai et al., 2003; Liu and Bagchi, 2004; Sharma et al., 2005, 2006). These “writers” have unique functions, with limited redundancy. The family members and their substrates are described in Figures 2, 3A.

#### KMTs

The KMTs include seven major families (KMT1–7) that alter HMs to activate or inactivate oncogenes and TSGs to either slow down or accelerate BC progression (Supplementary Table 1).

Active histone H3K4 methylation is performed by the KMT2 family and SET7 (KMT7) (Wilson et al., 2002; Shilatifard, 2012). Increased or decreased MLL4-mediated H3K4me1 at the boundaries in CpG islands in normal cells results in the gain or loss of DNA methylation encroachment in cells (Skvortsova et al., 2019). However, extensive recombinant human cancer epigenome studies have shown that the CpG islands in gene promoter regions were hypermethylated, whereas the intergenic regions and CpG-poor promoters were hypomethylated (Stirzaker et al., 2014). The KMT2 members individually or cooperatively promote estrogen-dependent gene activation, which mainly involves active histone methylation at the enhancer (H3K4me1/2) and promoter (H3K4me3) regions of oncogenes or pro-metastatic genes, and these processes contribute to BC proliferation and invasion (Jeong et al., 2011; Kim et al., 2014; Deb et al., 2016; Park et al., 2016; Su et al., 2016). Furthermore, MLL1 promotes the transcription of *TFF1* (an estrogen-dependent gene) via H3K4me1/2 at CpG islands of the enhancer region, which maintains a permissive chromatin structure for binding ERα and its pioneer factor (FOXA1). This process leads to chromatin relaxation to facilitate the binding of ERα and transcriptional activity in BC (Carroll et al., 2005; Hurtado et al., 2011; Jeong et al., 2014). Moreover, MLL3 cooperates with SET1A to bind to the *ESR1* promoter and epigenetically activate ERα transcription. Inhibition of MLL3 or SET1A significantly reduces ERα expression and reverses tamoxifen resistance (Kim S. S. et al., 2020). Finally, MLL2 is epigenetically involved in the acquisition of lapatinib resistance; specially, lapatinib induces the expression of a general anti-tumor transcription factor (FOXO), which paradoxically increases c-Myc transcription in a MLL2/GCN5/BRD4-dependent manner and ultimately causes lapatinib resistance (Matkar et al., 2015).

Methylation of H3K36 is catalyzed by ASH1L (KMT2H), NSD1–3 (KMT3B, F, G), SETD2 (KMT3A), and SMYD2 (KMT3C) (Hamamoto et al., 2004; Wagner and Carpenter, 2012; Zhu et al., 2016). NSD2 is highly expressed in BC and causes endocrine resistance via different pathways. Moreover, it is a positive regulator of ERα signaling and itself regulated by ERα, which creates a positive feedback regulatory loop. The NSD2 protein is recruited to the ERα promoter by BRD3/4 and facilitates ERα expression, although inhibition of BRD3/4 via JQ1 suppresses the ERα signaling pathway and growth of tamoxifen-resistant BC cells by disrupting the BRD/WHSC1/ERα axis (Feng et al., 2014). Endocrine resistance can also be induced via NSD2 control of H3K36me2, which activates various enzymes involved in glucose metabolism (e.g., *HK2*, *G6PD*, and *TIGAR*) and alters metabolism to enhance the pentose phosphate pathway. This ultimately promotes NADPH production and decreases the levels of reactive oxygen species (ROS) (Wang J. et al., 2016). Moreover, NSD2 promotes the survival and invasion of BC cells by activating *ADAM9* and *EGFR*, which leads to gefitinib resistance (an EGFR inhibitor) (Wang et al., 2019). Other H3K36 methylation writers are closely related to BC, although further studies are needed to clarify their roles in modulating HMs (Al Sarakbi et al., 2009; Newbold and Mokbel, 2010).

Histone H3K79 methylation is only catalyzed by DOT1L (KMT4), whereas there are no reported enzymes that can induce H3K79 demethylation (Feng et al., 2002). In BC, DOT1L increases H3K79me2, activates EMT-related transcription factors (*SNAIL*, *ZEB1*, *ZEB2*) and the *BCAT1*, which enhances cell migration, sphere formation, and the EMT (Cho et al., 2015; Oktyabri et al., 2016). Furthermore, DOT1L promotes ERα expression and ER-dependent gene transcription by promoting H3K79me2. Inhibition of DOT1L induces apoptosis and cell cycle arrest in hormone-responsive BC, and also reduces ERα expression and tumor growth in antiestrogen-resistant BC cells (Nassa et al., 2019).

**FIGURE 5:**
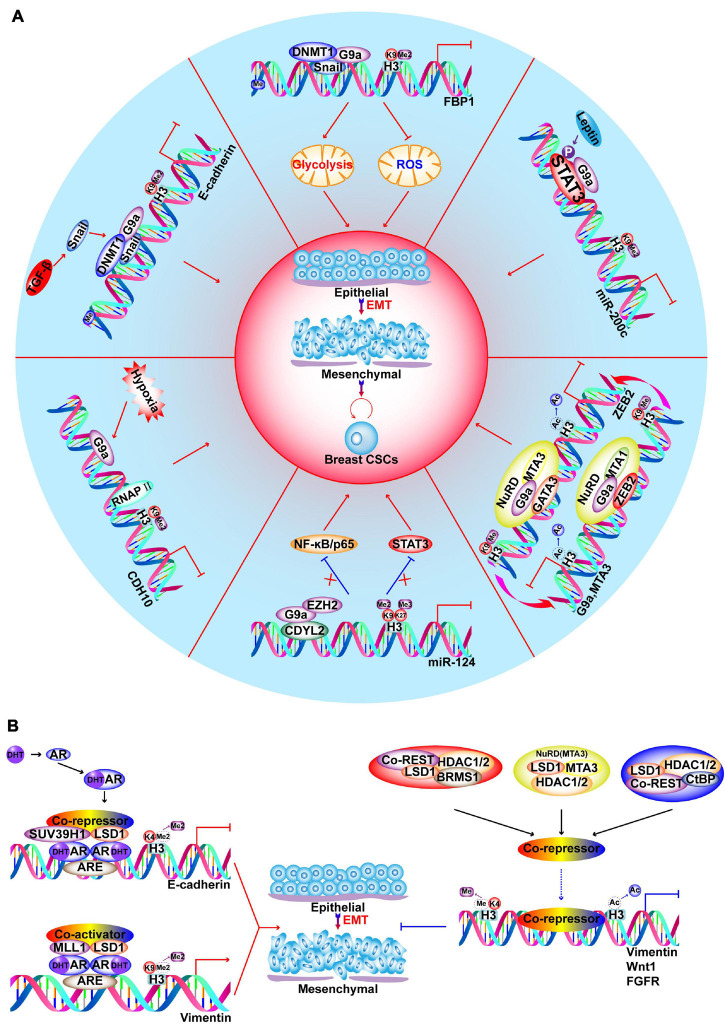
Functions of G9a and LSD1 in regulating EMT and CSC-like characteristics of BC. **(A)** G9a promotes EMT and development of CSCs via different pathways, such as hypoxia response, metabolic reprogramming, obesity induced leptin and TGFβ signaling stimulation. **(B)** Androgen receptor (AR) binds to AR-response elements (ARE) on target genes to recruit LSD1. On *E-cadherin* promoter, LSD1 demethylates H3K4 to repress gene expression; on *vimentin* promoter, LSD1 demethylates H3K9 to activate gene expression. LSD1 is a component of different corepressors complex, such as CoREST, CtBP, NuRD and SIN3A/HDAC. BRMS1-LSD1/CoREST/HDAC1/2 complexes cooperate in suppressing *Vimentin*; SIX3/LSD1/NuRD (MTA3) complexes coorperate in repressing *Wnt1*; ZNF516-CtBP/LSD1/CoREST complexes cooperate in inhibiting *EGFR* oncogene to inhibit proliferation, invasion and EMT of breast cancer cells.

Inactive H3K9 methylation is performed by the KMT1 family and forms heterochromatin to repress transcription (Rea et al., 2000; Schultz et al., 2002; Rice et al., 2003; Falandry et al., 2010; Shinkai and Tachibana, 2011). In this context, G9a promotes the EMT and invasive CSC properties via diverse mechanisms, including epigenetic silencing of epithelial markers (Dong et al., 2012), hypoxia response (Casciello et al., 2020), metabolic reprogramming (Dong et al., 2013), and obesity-mediated BC progression (Figure 5A; Chang et al., 2015; Si et al., 2015; Siouda et al., 2020). In *BRCA1*-mutated BC, EHMT1 (KMT1D) is up-regulated, leading to H3K9 methylation and decrease in GCN5-mediated H3K9ac, which synergistically promote the inhibition of phosphatidylethanolamine N-methyltransferase to potentiate BC tumorigenesis (Li D. et al., 2014).

Histone H4K20 methylation is mediated by the KMT5 family to repress transcription, and H4K20me3 is significantly decreased in BC, where it independently predicts a poor prognosis (Jørgensen et al., 2013; Yokoyama et al., 2014). Decreased H4K20me3 increases the invasiveness of BC cells, which can be reversed by upregulating the expression of SUV420H1/SUV420H2. Moreover, SUV39H2 reduces the EMT and BC cell invasiveness via transcriptional silencing of *Tensin-3*, *EGR1*, and *CTGF* (Shinchi et al., 2015; Wu et al., 2019). In this context, the EMT is potentiated by up-regulation of SET8 and its cooperation with Twist, while SET8-mediated H4K20me1 plays dual roles in regulating the expression of Twist-regulated genes, inactivating E-cadherin and activating N-cadherin. The diverse biological effects of H4K20me1 may related to different readers and other cofactors (Yang et al., 2012). Inhibition of SET8 using a specific antagonist or siRNA effectively overcomes paclitaxel resistance via Wnt/β-catenin signaling, although further studies are needed to confirm whether SET8 regulates the Wnt signaling pathway through epigenetic mechanisms (Wang et al., 2017).

**FIGURE 6:**
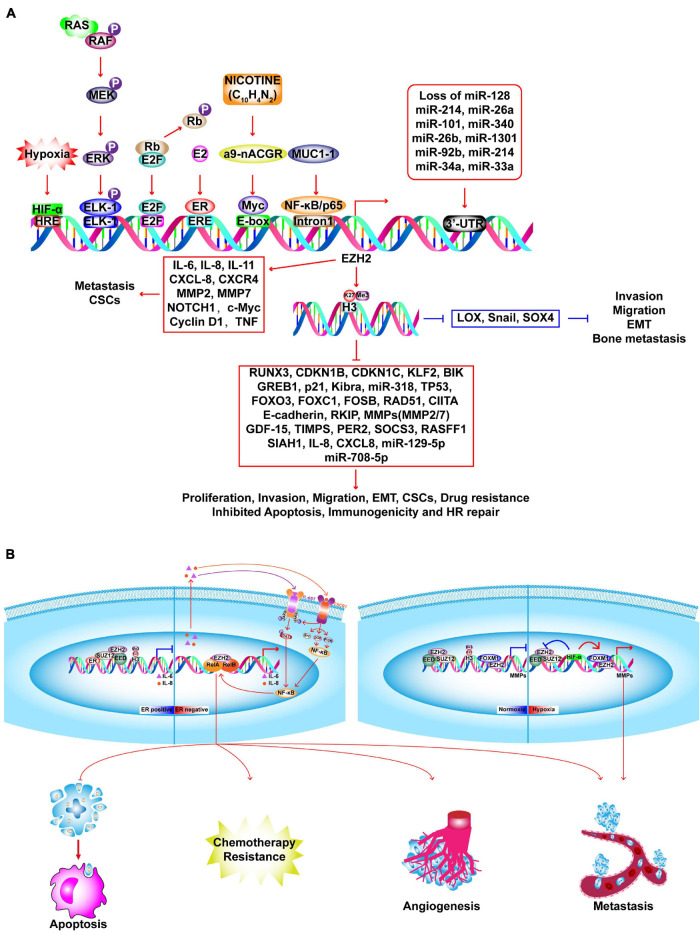
Molecular insights into EZH2-driven BC tumorigenesis. **(A)** In BC cells, EZH2 expression is upregulated by several factors and loss of microRNAs. Upregulated EZH2 elevate the H3K27me3 and transcriptional repression of several TSGs, such as *RUNX3*, *CDKN1B*, *CDKN1C*, which promote the proliferation, invasion, migration, EMT, CSCs, drug resistance and inhibit apoptosis, immunogenicity and HR repair. EZH2 promotes activation of oncogenes in a PRC2-independent manner, such as *IL-6*, *CXCL-8*, *NOTCH1*, *MMP2/7*, etc. EZH2 suppresses invasion, migration, EMT, and bone metastasis via inhibiting *LOX*, *SNAIL*, and *SOX4*. **(B)** EZH2 executes context-dependent activation which can be either dependent or independent on its methyltransferase activity. In ER-positive BC, ER recruits PRC2 complex to the promoter of NF-κB target genes (*IL-6*, *IL-8*) to inactivate transcription. In ER-negative basal-like BC, EZH2 acts as a co-activator of RelA and RelB to promote the expression of *IL-6*, *IL-8*, and *IL-11* which in turn activates NF-κB signaling pathway through a positive feedback leading to constitutive activation of these genes and anti-apoptosis, angiogenesis, metastasis, and chemotherapy resistance. In TNBC, upon normoxia, PRC2 inactivates matrix metalloproteinase gene (*MMPs*) by catalyzing H3K27me3 at the promoter region. Upon hypoxia, HIF1-α inhibits PRC2 activation by repressing protein expression of SUZ12 and EED, leading to functional switching to EZH2/FOXM1-depedent induction of MMPs expression.

The most comprehensively investigated methyltransferase, EZH2 is the catalytic component of polycomb repressive complex 2 (PRC2), which generates H3K27me3 via its SET domain and contributes to transcriptional suppression. The PRC2 core complex consists of EED, SUZ12, NURF55, Rbap46/48, and two catalytic constituents: EZH2 (KMT6A) and EZH1 (KMT6B) (Margueron and Reinberg, 2011). Relative to normal cells, BC cells have upregulated expression of EZH2 mRNA and protein; and increased protein expression is associated with aggressiveness and poor clinical outcomes. Furthermore, EZH2 expression is an independent predictor of BC recurrence with its levels steadily increasing from the normal epithelium to epithelial hyperplasia, DCIS, IDC, and distant metastasis (Ding and Kleer, 2006). Overexpression of EZH2 promotes anchorage independent cell growth and invasion, which require an intact SET domain and HMT activity, and is correlated with accelerated proliferation and reduced differentiation in BC (Kleer et al., 2003; Raaphorst et al., 2003; Bachmann et al., 2006; Collett et al., 2006; Gong et al., 2011; Wu and Crowe, 2015; Granit et al., 2018). After radiation therapy, EZH2-positive patients with inflammatory BC have a significantly lower 5-year locoregional free-survival rate than those with no EZH2 expression (Debeb et al., 2014). In conclusion, EZH2 may be a clinical biomarker to identify patients at high risk for BC before histological alterations occur. Figure 6A describes the abnormal expression of EZH2 and its functions as a tumor promoter or suppressor via methyltransferase-dependent or -independent mechanisms, as well as the effects on transcriptional activation in BC. In addition to its transcriptional inhibition function, which is mediated by H3K27me3, EZH2 also has context-dependent activation functions that are independent of its methyltransferase activity, which can be related to ER status or hypoxia (Figure 6B; Lee et al., 2011; Hartman et al., 2013; Mahara et al., 2016; Huang et al., 2018; Jiang et al., 2019). Therefore, BC cells are more sensitive to EZH2 degradation, but resistant to EZH2 inhibitors. The activity of EZH2 can be up- or down-regulated by phosphorylation at various sites, which may be a strategy for influencing the catalytic activity of EZH2 (Cha et al., 2005; Yang et al., 2015; Ko et al., 2016; Wan et al., 2018). PARP1 causes PARylation of EZH2 and leads to down-regulation of EZH2 and EZH2-mediated CSC characteristics (Yamaguchi et al., 2018). However, treatment of BC using PARPi mediates EZH2 activity and is associated CSC enrichment, which can abrogate the therapeutic efficacy of the PARPi. A combination of an EZH2 inhibitor (EZH2i, SHR2554) and PARPi (SHR3162) exert a synergistic effect on BC, which will be tested in a phase II clinical trial (NCT04355858).

Paradoxically, EZH2 can be negatively correlated with H3K27me3 in BC, and high EZH2 with low H3K27me3 may predict poor outcomes even in ER-positive BC (Wei et al., 2008; Holm et al., 2012; Bae et al., 2015). This may be related to non-canonical methylation of non-histone substrates by EZH2 (Bae and Hennighausen, 2014). Increased EZH2 expression in tumors is due to the combination of tumor proliferation and H3K27me3, which can ensure H3K27me3 homeostasis (Wassef et al., 2015). However, relative to more differentiated BC subtypes, TNBC with lower H3K27me3 may reflect fewer Polycomb gene targets in tumor cells (Holm et al., 2012). TNBC cells are thought to originate from luminal progenitor cells, which is compatible with the discovery that H3K27me3 is relatively low in stem/progenitor cells and is upregulated in luminal lineages (Lim et al., 2009; Pal et al., 2013).

#### PRDM Family of Methyltransferases

In humans, the PRDM family contains 17 members, which typically share a common structure that involves an N-terminal PR domain. This domain has similar structure and function to those of the SET domain, although its methyltransferase activity is restricted to lysine residues (Mzoughi et al., 2016). Existing studies have shown that PRDM2, PRDM3, PRDM8, PRDM9, and PRDM15 have methyltransferase activity toward H3K9me1/3, although none of these enzymes are known to play a role in BC via influencing HMs. However, these enzymes can play oncogenic and tumor suppressor roles in BC (He et al., 1998; Gazzerro et al., 2006; Taniguchi et al., 2017). Therefore, epigenetic mechanisms related to PRDMs are a promising field for BC-related research.

#### PRMT Family of Arginine Methyltransferases

The PRMT family contains nine members that are divided into three types: type I (PRMT1–4, PRMT6, and PRMT8), type II (PRMT5 and PRMT9), and type III (PRMT7). Type III PRMTs catalyze the formation of a mono-methylated intermediate, type I PRMTs catalyze the generation of asymmetric dimethylarginine, and type II PRMTs catalyze the formation of symmetric dimethylarginine. PRMTs regulate a myriad of biology process, including signal transduction, gene transcription, DNA repair, and mRNA splicing (Yang and Bedford, 2013). Furthermore, PRMTs modulate BC progression by regulating the transcription of oncogenes and TSGs (Supplementary Table 1). Although histone lysine methylation and acetylation are dynamically reversible processes, there are no known enzymes that can reverse arginine methylation, which is another promising avenue for further study.

### Histone Methylation Erasers

Histone lysine methylation was considered irreversible before the discovery of LSD1 (KDM1A) (Shi et al., 2004). However, there are eight main KDM families. The family members and substrates are described in Figures 2, 3A. In addition to the KDM1 family, all other KDMs are Fe^2+^/oxoglutarate-dependent enzymes that contain a JumonjiC (JmjC) domain (Tsukada et al., 2006). H3K4me1/2 and H3K4me2/3 can be demethylated by KDM1 and KDM5, respectively; furthermore, H3K9me1/2 is also demethylated by LSD1 (Shi et al., 2004; Metzger et al., 2005; Christensen et al., 2007; Garcia-Bassets et al., 2007; Fang et al., 2010). LSD1 is a member of various transcriptional corepressors including the RE1-silencing transcription factor corepressor (CoREST), NuRD, CtBP, SIN3A, and HDAC complexes. In this context, LSD1 suppresses the transcription of various genes that are involved in BC cell proliferation and motility (Lee et al., 2005; Wang et al., 2007, 2009; Chen M. S. et al., 2017; Qiu et al., 2018; Yang et al., 2018; Zheng et al., 2018). Nevertheless, LSD1 also promotes androgen-dependent transcriptional activation via demethylation of inactive H3K9me2, which facilitates EMT and metastasis (Figure 5B; Feng et al., 2017). In TNBC, LSD1 epigenetically silences chemokines that attract cytotoxic T-cells, as well as *PD-L1*, while inhibition of LSD1 improves T-lymphocyte trafficking to the tumor microenvironment and the response to immune checkpoint blockade (Qin et al., 2019). Inhibition of LSD1 is also a promising strategy in both ER-positive and ER-negative BC, as LSD1 demethylase activity is significantly associated with ER and ER-dependent transcriptional activity, as well as enhanced BC proliferation (Pollock et al., 2012; Bai J. W. et al., 2017). Moreover, inhibition of histone demethylation and deacetylation exerts synergistic effects on the regulation of gene expression in BC (Huang et al., 2012; Vasilatos et al., 2013).

Relative to LSD1, there are fewer studies on the role of LSD2 in BC, although LSD2 expression is significantly elevated in BC tissues (Katz et al., 2014). Furthermore, upregulation of LSD2 in TNBC cells promotes the expression of other epigenetic modifiers, such as LSD1, HDAC1/2, and DNMT3B, which enhance cell proliferation ability and CSCs traits, but repress invasion and motility (Chen L. et al., 2017). However, the mechanisms underlying the epigenetic modifications and their opposing roles are unclear.

Histone H3K36 demethylation is catalyzed by KDM2 (H3K36me1/2), KDM4 (H3K36me2/3), and JMJD5 as the only member of the KDM8 family (H3K36me2/3) (Chen et al., 2006; Tsukada et al., 2006; Whetstine et al., 2006; Hsia et al., 2010). In BC, KDM2A is generally upregulated and plays dual roles in regulating ribosomal RNA (rRNA) transcription. Cancer cells exhibit accelerated transcription of ribosomal DNA (rDNA) to build more ribosomes and synthesize more protein to fulfill the demands of rapid cellular division (Thomas, 2000; Ruggero and Pandolfi, 2003). However, in a state of mild glucose starvation, KDM2 inhibits rRNA transcription by demethylating H3K36me2 at its promoter, and ultimately suppresses BC cell proliferation (Tanaka et al., 2015). In contrast, SF-KDM2A (a variant of the KDM2A protein) lacks a JmjC domain and has no demethylase activity. Binding of SF-KDM2A to the rRNA promoter downregulates H4K20me3 in a zf-CXXC domain-dependent manner and activates rRNA transcription, which promotes BC tumorigenesis (Okamoto et al., 2017). When SF-KDM2A binds to the rDNA promoter, SF-KDM2A may modulate the H4K20 methylation state via effects on various H4K20me3 writers, such as SUV420H1/2 and SET8. Furthermore, JHDM1B also inhibits the transcription of rRNA and knockdown of JHDM1B causes increased rRNA transcription and enhanced proliferation in p53-compromised BC cells, while p53-competent cells undergo cellular senescence and death (Penzo et al., 2015). Knockdown of JHDM1B also induces significant 45S pre-rRNA transcription and processing via increased H3K36me2 levels at rDNA loci, as well as changes in DNA methylation at specific CpG sites in rDNA genes. JHDM1B knockdown and increased ribosome generation confers aggressiveness in BC cells (Galbiati et al., 2017).

Inactive H3K9 is demethylated by the proteins belonging to KDM3, KDM4, and KDM7 families. The KDM3 family demethylates H3K9me1/2, whereas the KDM4 family has broader demethylase activities that targets H3K9me2/3 and H3K36me2/3 (Chen et al., 2006; Whetstine et al., 2006; Cloos et al., 2008). The KDM7 family includes JHDM1D (KDM7A), PHF8 (KDM7B), and PHF2 (KDM7C). JHDM1D and PHF8 demethylate H3K9me1/2, H3K27me1/2, and H4K20me1/2, whereas PHF2 (KDM7C) demethylates H3K9me1/2, H3K27me1/2, and H4K20me3 (Horton et al., 2010; Qi et al., 2010; Tsukada et al., 2010; Wen et al., 2010; Stender et al., 2012). During BC transformation, the level of KDM3A is increased, which is accompanied by decreased H3K9me2/3. Furthermore, KDM3A promotes BC cell proliferation and migration via activation of *MYC*, *PAX3*, *Cyclin D1*, *MMP-9*, *S100A4*, and *JUN* (Zhao et al., 2016; Qin et al., 2017; Ramadoss et al., 2017). Interestingly, KDM3A can exert a tumor suppressor function via inducing anoikis in BC, which is a special form of apoptosis that occurs when epithelial cells lose attachment to the extracellular matrix or attach to an inappropriate extracellular matrix (Pedanou et al., 2016). Moreover, KDM3A activates ER-target genes and promotes ER-positive BC growth and resistance to endocrine therapy, whereas KDM3A and KDM4B interact to co-regulate pro-proliferative and ER-target genes. Inhibition of both KDM3A and KDM4B provide a greater effect on ER activity and cell growth, which may be an effective strategy for treating ER-driven BC (Mahajan et al., 2014; Wade et al., 2015; Jones et al., 2019). In this context, KDM4B is a constituent of the MLL2 complex that coordinates ERα transcription, where H3K9 demethylation is a precondition for H3K4 methylation. Depletion of KDM4B impairs the estrogen-induced G1/S transition in the cell cycle; and since KDM4B is a target of ER transcription, it may reflect a positive feedback loop that promotes hormone response to BC carcinogenesis (Shi et al., 2011).

In BC, upregulated KDM4A reduces centromere-associated H3K9me3, which impairs the maintenance of pericentromeric heterochromatin and contributes to chromosome instability, and these steps lead to tumor progression and drug resistance (Slee et al., 2012). Hypoxia-driven copy gains are closely dependent on KDM4A demethylase activity, which leads to heterogeneity in copy number and expression. This process can be reversed by KDM4A inhibitors, which may be useful co-therapeutics to suppress copy gains (Black et al., 2015). Expression of KDM4C is higher in TNBC, where it promotes tumor growth in a demethylase-dependent manner (Liu G. et al., 2009; Garcia and Lizcano, 2016). In addition, KDM4C functions as the co-activator of HIF-1α to trigger transcription of genes related to metabolic reprogramming and metastasis, such as *BNIP3*, *LDHA*, *PDK1*, *SLC2A1*, *LOXL2*, and *L1CAM* (Luo et al., 2012).

PHF8 usually functions as an oncogene in BC, and promotes proliferation and EMT via H3K9me1/2 demethylation (Wang Q. et al., 2016; Shao et al., 2017). Expression of PHF8 is upregulated by HER2 in HER2-positive BC. In turn, PHF8 functions as a coactivator to increase expression of HER2, which drives the EMT and cytokine production. The HER2-PHF8-IL6 regulatory axis also contributes to tumorigenesis and trastuzumab resistance (Liu et al., 2020).

Demethylation of H3K27 is mainly catalyzed by the proteins of the KDM6 family, which includes UTX (KDM6A), JMJD3 (KDM6B), and UTY (KDM6C). The UTX and JMJD3 members act on H3K27me2/3, whereas UTY has weak demethylase activity toward H3K27me3. These demethylation activities reduce the repression of oncogenes and TSGs (Agger et al., 2007; Walport et al., 2014). In BC, UTX generally inhibits the EMT and acquisition of CSCs properties by activating *CDH10* and *DICER* (Taube et al., 2017; Yu W. et al., 2019). In addition, UTX is a known component of the MLL complex, where it inhibits the EMT in a H3K27me3-independent manner. Furthermore, UTX forms a transcriptional repression complex with LSD1, DNMT1, and HDAC1, which represses H3K4me2 and H3 acetylation to silence *SNAIL*, *ZEB1*, and *ZEB2* (Choi et al., 2015). Transactivation of UTX is also performed by ER, which creates a positive feedback loop in the regulation of the hormone response (Xie et al., 2017).

Similar to UTX, JMJD3 (KDM6B) expression is induced by TGF-β, and epigenetically activates *SNAIL1* transcription to facilitate the EMT (Ramadoss et al., 2012). In this context, regulators of H3K27 methylation play an antagonistic role in dynamically regulating the expression of certain genes. Demethylation of H3K27me3 by KDM6B promotes expression of the *IGFBP5* oncogene, and antagonizes EZH2-mediated repression, whereas pharmacological inhibition of KDM6B augments the apoptotic response to PI3K/AKT inhibitor treatment (Wang et al., 2018).

### Histone Methylation Readers

Methylated histone lysine is read and interpreted by the reader proteins that contain various specialized recognition motifs. Similar to BRD-containing proteins, several methyllysine readers have been implicated in BC (Supplementary Table 2). These proteins serve as a scaffold to recognize HMs and recruit different protein complexes to modulate the transcription of oncogenes and TSGs, which can either promote or suppress BC development. For example, PHF20L1 contains a TUDOR domain that reads H3K27me2 and leads to transcriptional repression by recruiting the PRC2 and the NuRD complex, which links PRC2-mediated methylation and NuRD-mediated deacetylation of H3K27. Furthermore, PHF20L1 may play an oncogenic role in the response to hypoxia by promoting glycolysis, proliferation, and metastasis of BC cells via direct inhibition of various tumor suppressors, such as *HIC1*, *KISS1*, and *BRCA1* (Hou et al., 2020). These findings were used to develop small molecules that could disrupt these protein-protein interactions, such as UNC1079, UNC1215, and UNC2533, although these inhibitors of methyllysine readers have not been tested as a potential treatment for BC.

## Other Types of HMs

Histone phosphorylation is written by kinases and removed by phosphatases and thus, read by specific phosphorylation binding domain, which is involved in chromatin condensation and transcription regulation (Giet and Glover, 2001; Nowak et al., 2003; Soloaga et al., 2003; Baek, 2011). In cellular processes, histone phosphorylation is involved in mitosis, DNA repair, replication, and transcription. Upregulated promoter region H3S10p activates oncogenes, such as *RUNX1, DNMT1, SNAIL*, and *HER2*, which promote BC progression (Mishra et al., 2001; Hsu et al., 2013; Gupta et al., 2014; Del Barco et al., 2018). Several Aurora kinase inhibitors have been discovered and tested in BC, including PF-03814735, GSK1070916, and PHA-680632, which effectively repress proliferation and promote polyploid formation, ultimately contributing to potentiated apoptosis and reduced tumor growth (Soncini et al., 2006; Hardwicke et al., 2009; Jani et al., 2010).

Ubiquitination modification is mediated by sequential catalytic reactions of three enzymes: E1 activating, E2 conjugating, and E3 ligase enzymes. All histones can be mono- or polyubiquitinated on different lysine residues; however, the most understood are mono-ubiquitination of H2A and H2B, especially H2AK119ub1 and H2BK120ub1. H2AK119ub1 is catalyzed by RING1A/B which are components of polycomb repressive complex 1 (PRC1) and involved in transcriptional inhibition, chromatin compaction, and DNA damage repair, while it is removed by BAP1 and USP16 deubiquitinase (Wang et al., 2004; Scheuermann et al., 2010; Chagraoui et al., 2011; Kakarougkas et al., 2014; Gu et al., 2016). BRCA1 protein has E3 ligase activity to catalyze H2Aub1 to sustain heterochromatin status and silence PR-target genes (Calvo and Beato, 2011; Zhu et al., 2011). Another E3 ligase, TRIM37 is overexpressed in BC and promotes tumorigenesis by silencing TSGs in a H2AK119ub1-dependent manner together with PRC2 and PRC1 (Bhatnagar et al., 2014). However, H2BK120ub1 is associated with transcription elongation, DNA damage repair and is catalyzed by RNF20/40 E3 ligase, and removed by many deubiquitianses (USP2/22/27X/36/42/43/44/49/51) with USP22 being the most characterized one (Zhu et al., 2005; Kim et al., 2009; Nakamura et al., 2011; Jeusset and McManus, 2017; Li et al., 2018). In active genes, H2BK120ub1 usually coexists with H3K4 and H3K79 at transcriptional start site and transcribed regions (Sun and Allis, 2002; Kim et al., 2005, 2009; Valencia-Sánchez et al., 2019). H2Bub1 has dual roles in breast tumorigenesis in different BC sub-types (Prenzel et al., 2011; Zhang et al., 2011). In TNBC, high H2Bub1 have improved survival, whereas the opposite results are seen in luminal sub-type. In TNBC cells, reduced *RNF20* promoted the expression of *IL-6* and *IL-8* via NF-κB pathway. While in luminal BC cells, silencing of *RNF20* inhibited the expression of ER-targeted genes, such as *PGR*, *CXCL12*, and *FOXA1*, which promote proliferation and migration (Tarcic et al., 2017). In HER2-positve BC, RNF40 and H2Bub1 promote breast tumorigenesis by transcriptionally activating genes associated with enhanced actin-cytoskeleton dynamics (Wegwitz et al., 2020). The deubiquitinases of H2Bub1, USP22, USP51, USP27X, and USP44 promote BC proliferation, invasion, and tumor growth (Atanassov et al., 2016; Lan et al., 2016). The PHD finger of UBR7 can catalyze H2BK120ub1 on cell adhesion genes (*CDH4*), and inhibit the Wnt/β-Catenin signaling pathway to inhibit invasion, metastasis and EMT (Adhikary et al., 2019). Ubiquitinase inhibitors have effective anti-cancer roles against BC. For example, E2 RAD6B inhibitor, twelve triazine analog, effectively inhibits MDA-MB-231 BC cell proliferation, migration, and colony formation (Sanders et al., 2013).

Histone citrullination/deimination is characterized as a conversion of peptidyl-arginine to peptidyl-citrulline mediated by peptidyl arginine deiminase (PAD) enzymes (Fuhrmann et al., 2015). Although it is a reversible process, there is no known citrulline eraser. PAD4 is upregulated in BC and deiminates R2, R8, and R17 of histone H3, which inhibits the methylation of these residues by PRMT4, leading to transcriptional silence of target genes. In turn, methylation of arginine inhibits deamination (Cuthbert et al., 2004; Chang and Han, 2006; Chang et al., 2009). PAD2 is also upregulated in BC and regulates transcription by deiminating R2, R8, and R17 at promoter regions (Cherrington et al., 2012). Upon estradiol, ER recruits PAD2 to target promoters to activate transcription by citrullinating H3R26 (Zhang X. et al., 2012). More importantly, PAD2 promotes migration of BC cells, which can be reversed by the PAD2 inhibitor BB-Cl-Amidine (Horibata et al., 2017). PAD2 is upregulated in tamoxifen-resistant BC cells, and silencing PAD2 restores tamoxifen sensitivity. Furthermore, PAD2 inhibitor Cl-amidine effectively overcomes tamoxifen resistance and enhances the efficacy of docetaxel (Li et al., 2019).

Histone O-GlcNAcylation is dynamically regulated by O-linked β-N-acetylglucosamine (O-GlcNAc) transferase (OGT) and the glycoside hydrolase O-GlcNAcase (OGA) to add or remove O-GlcNAc to Ser or Thr. This modification is involved in mitosis, chromatin dynamics, and gene expression by crosstalk with other HMs (Sakabe and Hart, 2010; Nakamura et al., 2011; Fong et al., 2012; Xu Q. et al., 2014; Rønningen et al., 2015; Wang et al., 2015). In BC, the expression of OGT is higher in poorly-differentiated tumors, and inhibition of OGT can inhibit tumor growth (Caldwell et al., 2010; Krześlak et al., 2012). However, no clear roles of histone O-GlcNAcylation in BC progression have been found.

Sumoylation is characterized by the covalent attachment of a small ubiquitin-like modifier (SUMO) to lysine resides in histone tails which results in transcriptional silencing. It is catalyzed by four isoforms SUMO named SUMO-1, -2, -3, -4 and is reversed by sentrin-specific proteases (Kim et al., 2000; Tatham et al., 2001; Shiio and Eisenman, 2003; Yeh, 2009). Neddylation refers to the conjugation of NEDD8, a protein highly homologous to ubiquitin, to lysine residues of histone tails. Both histone H4 and H2A neddylation are catalyzed by E3 ligases RNF111 and RNF168, respectively, are involved in regulating DNA damage repair (Ma et al., 2013; Li T. et al., 2014).

Histone biotinylation is a rare modification of lysine residues that is catalyzed by biotinidase and holocarboxylase synthetase (Hymes et al., 1995; Hassan and Zempleni, 2008). Biotinylation of histone is involved in cell proliferation, DNA damage, apoptosis, and silencing of transcription (Xu Y. M. et al., 2014). Although biotinylation is a reversible process, debiotinylase has not been discovered.

Histone can be mono- and/or poly-ADP-ribosylated by PARPs, which hydrolyze nicotinamide adenine dinucleotide and transfer ADP-ribose to lys/glu/asp residues (Messner and Hottiger, 2011). Histone ADP-ribosylation can be reversed by ADP-ribosylhydrolases and PAR glycohydrolases. ADP-ribosylation of histones influence DNA repair, replication and transcription.

Proline residues exist in two different peptide bond conformations: *cis* and *trans*, which are regulated by prolyl *cis*-*trans* isomerization. This process is catalyzed by peptidyl-prolyl *cis*-*trans* isomerases. Proline isomerization has been extensively studied in non-histones, but rarely in histones. Proline isomerase Fpr4 is identified to catalyze isomerization of H3 proline P30 and P38 in yeast. The isomerization of H3P38 inhibits the SET2 methyltransferase activity of catalyzing H3K36me3, and contributes to enhanced transcription of target genes (Nelson et al., 2006).

Glutathionylation is a reversible process that adds glutathione to cysteine residues which is involved in DNA compaction, cell cycle, and DNA repair. Glutathionylation on cysteine of histone tails was first discovered in treatment of doxorubicin-resistant BC cell line with nitrosoglutathione, which reversed the resistance by enhanced histone glutathionylation and accumulation of doxorubicin in the nucleus (de Luca et al., 2011).

In addition to acetylation, lysines can also be modified by formylation, propionylation, butyrylation, crotonylation, 2-hydroxyisobutyrylation, β-hydroxybutyrylation, succinylation, malonylation, glutarylation, benzoylation, and the recently discovered isobutyrylation (Zhu et al., 2021). Similar to acetylation, histone butyrylation (Goudarzi et al., 2016), crotonylation (Tan et al., 2011), 2-hydroxyisobutyrylation (Dai et al., 2014), and β-hydroxybutyrylation (Xie et al., 2016) promote chromatin decompaction and transcriptional activation. These non-acetyl histone acylations are catalyzed by KATs and HDACs or can occur in an enzyme-independent manner, which means no specific enzymes have been discovered (Moellering and Cravatt, 2013; Wagner and Payne, 2013; Choudhary et al., 2014; Zhao et al., 2018). This prompts us to consider whether these newly discovered modifications have new functions or they are functionally redundant with acetylation. Furthermore, they are also involved in spermatogenesis, kidney injury, ketogenesis, depression, HIV latency, and cancer progression (Sabari et al., 2017; Wan et al., 2019). However, few roles of these modifications described in this section have been studied in BC, and further explorations are needed.

## Crosstalk Among HMs in BC

**FIGURE 7:**
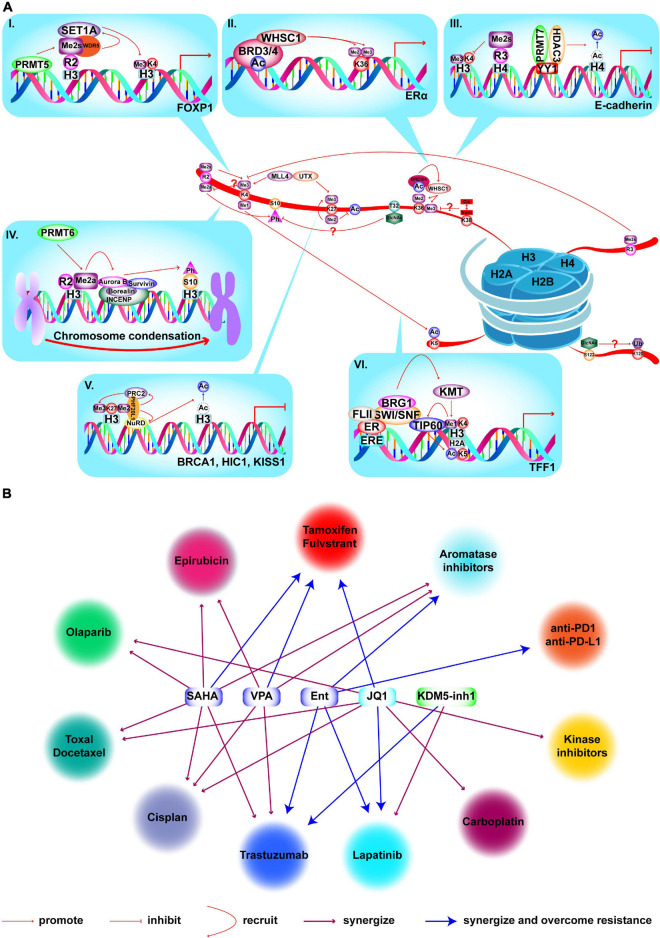
Crosstalk among HMs and combined treatments with epi-drugs in BC. **(A)** Crosstalk among HMs and mechanisms in BC. (I) PRMT5 is recruited to *FOXP1* promoter to catalyze H3R2me2s which is recognized by WDR5. WDR5 further recruits SET1 to catalyze H3K4me3 to activate transcription which results in enhanced BC stem cell function. (II) WHSC1 is recruited to *ER*α promoter by BRD3/4 and catalyze H3K36me2/3 to promote transcription and tamoxifen resistance. (III) PRMT7 and HDAC3 are recruited by YY1 to *E-cadherin* promoter and catalyze H4R3me2s and deacetylation of H4. Elevated H4R3me2s antagonizes H3K4me3 deposition and HDAC3-mediated deacetylation to inactivate *E-cadherin* transcription and lead to BC metastasis and EMT. (IV) PRMT6 catalyzes H3R2me2a to recruit the chromosomal passenger complex that includes Aurora B, Borealin, INCENP, and Survivn to catalyze H3S10p to promote chromosome condensation. (V) PHF20L1 promotes BC tumorigenesis by coordinating corepressor complex complex. PHF20L1 recognizes H3K27me2 to recruit PRC2 and NuRD complex which catalyze H3K27me3 and deacetylation of H3K27 to inactivate tumor suppressors transcription, such as *HIC1*, *KISS1*, and *BRCA1*. (VI) After E2 stimulation and MLL1 recruitment, MLL1 catalyzes H3K4me1 and facilitates the recruitment of TIP60 which catalyzes H2AK5ac to promote transcription of ERα target genes. Crosstalk which is marked by “?”, has not been studied for its role in BC. **(B)** Existing epi-drugs that either synergize or overcome resistance to other anti-cancer drugs in treatment of BC.

As mentioned above, histones are modified by at least 23 different PTMs, which occur in a variety of combinations. This means that a single mark not only executes active or suppressive functions, but can also affect the deposition or recognition of other marks, which is referred to crosstalk. Therefore, it is important to clarify whether the mark is a driving or acting factor for its application in the treatment of BC (Figure 7A). First, one mark can antagonize the deposition of other marks. For example, elevated H4R3me2s mediated by PRMT7 antagonize MLL4-mediated H3K4me3. PRMT7 is co-recruited with HDAC3 and co-participates in *E-cadherin* silencing by inhibiting H3K4me3 and H4ac, leading to EMT and metastasis in BC (Yao et al., 2014). Furthermore, methylation of H3K4 and H3R2me2a are mutually exclusive marks, but the effect of this coexistence on BC has not been studied (Guccione et al., 2007; Hyllus et al., 2007). Second, HMs also cooperatively regulate transcription in a sequential process. PRMT5 is recruited to the *FOXP1* promoter to catalyze H3R2me2s which is recognized by WDR5 and further recruits SET1 to catalyze H3K4me3 to active transcription, leading to increased BC stem cells (Chiang et al., 2017). Furthermore, combinations also occur in different kinds of HMs. MLL1-mediated H3K4me1 facilitates the recruitment of TIP60, which further catalyzes H2AK5ac to activate transcription of ERα target genes in BC (Jeong et al., 2011). Some marks can directly regulate the expression of other modifiers. For example, LSD1 represses the expression of TRIM37, encoding a histone H2A ubiquitin ligase, by demethylating H3K4me2 and inhibit BC metastasis (Hu et al., 2019). Lastly, histone phosphorylation and methylation also promote chromosome condensation via crosstalk in BC. PRMT6-mediated H3R2me2a is demonstrated to promote H3S10p by Aurora B to enhance chromosome condensation (Kim S. et al., 2020). Crosstalk mechanisms between HMs are complicated and of great significance in BC, which requires further research. For instance, knockdown of LSD1 reduces expression of HDACs, while silencing HDAC5 causes accumulation of H3K4me2 in TNBC cells. Combination of LSD1 inhibitor (LSD1i) and HDACi leads to enhanced cell death compared to that achieved in monotherapy (Vasilatos et al., 2013). However, the clear mechanisms are unknown. Finally, some crosstalk events among O-GlcNAcylation and proline isomerization with other PTMs were discovered but not studied in BC, as listed in Figure 7A. In summary, understanding the specific mechanisms of crosstalk among HMs, and applying target inhibitors to the treatment of BC are the current difficulties that must be overcome.

## HM-Associated BC Therapy

Epigenetic drugs (epi-drug) prevent BC progression by restoring the aberrantly activated oncogenes or suppressed TSGs. Furthermore, epi-drugs help overcome drug resistance, enhance the effectiveness of other anti-cancer drugs, and improve radiotherapy effect (Figure 7B). Many epi-drugs have been tested in clinical trials and provided good outcomes, which are summarized in Supplementary Table 3.

### HAT Inhibitors

A few inhibitors targeting HATs have been studied but none have entered clinical trials. The currently available HATis include bi-substrate inhibitors, small molecule HATis (natural or synthetic), and library screened inhibitors (Supplementary Table 4). Bi-substrate mimics, such as Lys-CoA, have effective inhibitory ability but limited applications in cellular systems because of their large molecular weight (Lau et al., 2000; Cole, 2008). A newly synthesized compound (1r), which is based on C646, also provided more potent inhibitory ability, better drug-like characteristics, and less cellular proliferation after removal of the toxic nitro group (Liu et al., 2019). Instead of inhibiting acetyltransferase activity, ICG-001 targets the protein-protein interactions between CBP and β-catenin to prevent BC progression (Ring et al., 2018; Sulaiman et al., 2018; Fatima et al., 2019). The substrate specificity and acetyltransferase activity of HATs is determined by multi-subunit protein complexes. Unfortunately, these complexities pose a significant barrier to moving from *in vitro* experiments to *in vivo* experiments. The current inhibitors are also limited by their poor selectivity and low efficiency, although they may be useful starting points for developing new inhibitors.

### HDAC Inhibitors

Structural characteristics were used to create four categories of HDACis: hydroxamic acids, cyclic peptides, aliphatic fatty acids, and benzamides (Supplementary Table 4). Three hydroxamic acid HDACis have currently been approved by the US Food and Drug Administration (FDA): vorinostat (SAHA), belinostat (PXD101), and panobinostat (LBH-589). These drugs also exert anti-cancer effects in BC. For example, SAHA inhibits proliferation, invasion, migration, and EMT; in addition, it also induces cell cycle arrest, apoptosis, autophagy, differentiation, and anoikis (Huang and Pardee, 2000; Munster et al., 2001; Lauricella et al., 2012; Min et al., 2015; Lee et al., 2016; Wawruszak et al., 2019). Moreover, SAHA has shown remarkable efficacy in promoting response or overcoming resistance to tamoxifen (Hodges-Gallagher et al., 2007; Lee et al., 2012), cisplatin (Wawruszak et al., 2015), olaparib (Min et al., 2015), taxol (Shi et al., 2010), epirubicin (Marchion et al., 2004), docetaxel, and trastuzumab (Bali et al., 2005). Furthermore, SAHA effectively enhances apoptosis mediated by TNF-related apoptosis-inducing ligand (TRAIL), which involves overcoming Apo2L/TRAIL resistance or upregulating CD137 receptors and inducing anoikis (Butler et al., 2006; Bellarosa et al., 2012; Lauricella et al., 2012; Zhou et al., 2016). Paradoxically, SAHA also promotes the EMT and metastasis of TNBC cells by inhibiting HDAC8, which highlights the importance of caution when using SAHA to treat BC, given its potential to promote metastasis (Wu et al., 2016). Belinostat (PXD101) also inhibits proliferation and induces apoptosis via the Wnt/β-catenin and PKC pathways, with synergistic effects observed for a combination of belinostat and a HSP90 inhibitor (17-AAG) (Lu et al., 2019; Zuo et al., 2020). Panobinostat (LBH-589) can reverse the EMT in TNBC by inhibiting *ZEB1/2* (Rhodes et al., 2014). Other hydroxamic acid HDACis exist, such as resminostat, pracinostat, and abexinostat, although they have rarely been investigated in BC, and additional studies are needed to consider their application in this context.

Romidepsin (FK2280) is a cyclic peptide HDACi that is approved by the FDA. A combination of romidepsin and decitabine (a methyltransferase inhibitor) exerts synergistic inhibition of cell growth and induction of apoptosis (Cooper et al., 2012). In inflammatory BC, romidepsin treatment leads to destruction of tumor emboli and lymphatic vascular architecture via repression of VEGF and HIF-1α. Romidepsin also exerts synergistic effects with paclitaxel on primary tumor growth and metastatic lesions at multiple sites (Robertson et al., 2013).

Valproic acid (VPA) is the most widely studied aliphatic fatty acid HDACi, which can inhibit BC development by promoting cell cycle arrest and activating programmed cell death pathways (Fortunati et al., 2008; Travaglini et al., 2009). This drug also inhibits migration by repressing *Survivin* in a HDAC2-dependent manner (Zhang L. et al., 2012). However, VPA can also facilitate the EMT via upregulation of SNAIL and *ZEB1* in a HDAC2-dependent manner, although the underlying HDAC2-related mechanism remains unclear (Zhang et al., 2019). Moreover, VPA exerts synergistic effects when it is combined with other anti-cancer drugs, such as tamoxifen (Hodges-Gallagher et al., 2007; Fortunati et al., 2010), epirubicin (Marchion et al., 2005), cisplatin (Wawruszak et al., 2015), camptothecin (Arakawa et al., 2009), capecitabine (Terranova-Barberio et al., 2016), and hydroxyurea (Tian et al., 2017), which ultimately prevent BC progression.

Entinostat (Ent, MS-275) is a synthetic benzamide derivative HDACi that has a strong immunomodulatory effect in BC (Christmas et al., 2018; McCaw et al., 2019). Moreover, Ent treatment reverses the EMT and the tumor-initiating cell phenotype, which reduces tumorigenesis and metastasis (Shah et al., 2014; Schech A. et al., 2015). When combined with all-trans retinoic acid (ATRA) and doxorubicin, Ent improved the retinoic acid-mediated differentiation by inducing retinoid acid receptor expression and also enhanced doxorubicin-driven cytotoxicity; and Ent combined with ATRA also helped overcome aromatase inhibitor (AI) resistance by reducing the tumor-initiating cell population (Schech A. J. et al., 2015; Merino et al., 2016). Other newly synthetized multifunctional inhibitors have good anticancer effects. For example, a DNMT1 inhibitor (DC-517) and a SAHA-based hybrid compound (C02S) exhibit strong inhibitory activities against HDAC1, DNMT1, DNMT3A, and DNMT3B, which may help reverse abnormal methylation/acetylation and alleviate the repression of TSGs (Yuan et al., 2019). The HDAC/FGFR1 and HDAC1/CDK4/9 dual inhibitors also have excellent activity (Liu J. et al., 2018; Pires et al., 2018).

Sirtuin inhibitors can prevent BC progression via different targets, functions, and structures (Supplementary Table 4); furthermore, they can be combined with chemotherapeutic drugs to overcome multidrug resistance. For example, amurensin G inhibits SIRT1 and further suppresses FoxO1 and MDR1 expression in doxorubicin-resistant BC cells, which can potentiate the cellular uptake of doxorubicin and allow it to suppress oncogenic growth (Oh et al., 2010). Splitomicin reduces cell motility and potentiates the anti-motility activity of paclitaxel, which can be further potentiated by adding a HDAC6 inhibitor (trichostatin A, TSA) (Bonezzi et al., 2012). Researchers have also evaluated SIRT1/2 inhibitors, which include sirtinol, salermide, splitomicin, cambinol, suramin, tenovin, nicotinamide, indole derivatives, and structurally similar analogs. In BC, these compounds generally inhibit cell proliferation and cause p53-mediated apoptosis by upregulating its acetylation, or by inducing expression of some pro-apoptosis genes that are epigenetically silenced by SIRT1 (Peck et al., 2010). Therefore, various SIRT inhibitors may act synergistically with traditional anti-cancer drugs for treating BC. The variable pathways through which SIRTs overcome drug resistance may also suggest that a broad-spectrum SIRT inhibitors can be designed based on existing inhibitors to help balance specificity and effectiveness.

### Histone Acetylation Reader Inhibitors

Inhibitors targeting the BRDs of histone acetylation readers have mainly been developed against the BET family of BRD-containing proteins. The currently available BET inhibitors (BETis), their use for treating BC, and efficacy data are shown in Supplementary Table 4. The most studied BRD4 inhibitor in BC is JQ1, which shows effective anti-cancer functions and synergistic effects with other drugs, such as docetaxel, vinorelbine, cisplatin, carboplatin (Pérez-Peña et al., 2016), fulvestrant (Feng et al., 2014), lapatinib (Stuhlmiller et al., 2015), olaparib (Yang et al., 2017), SAHA (Zeng et al., 2016), mocetinostat (Borbely et al., 2015), volasertib (Sahni et al., 2017), everolimus (Bihani et al., 2015; Vázquez et al., 2017), rapamycin, torin (Marcotte et al., 2016), trametinib (Zawistowski et al., 2017), and GDC-0941 (Stratikopoulos et al., 2015). However, similar to traditional anti-cancer drugs, cells eventually gain resistance to BETis. For example, JQ1 and I-BET151 exert anti-cancer functions by reducing IKBKE expression to block the NF-κB signaling pathway, although tumor-associated macrophages in TNBC confound this effect by increasing IL-6 or IL-10/STAT3/IKBKE/NF-κB axis (Qiao et al., 2020). Therefore, combined inhibition of IKBKE or STAT3 with BETis may be more potent in treatment of TNBC by overcoming BETi resistance. Unfortunately, the short half-life of JQ1 has precluded clinical studies. However, the FDA and European medicines agency (EMA) have approved polymeric biomaterials for pharmaceutical applications, and JQ1-containing nanoparticles (made of poly-lactic-co-glycolic acid) have a prolonged half-life and enhanced activity against TNBC based on *in vivo* and *in vitro* models (Maggisano et al., 2019). Cells from TNBC that are BETi-resistant remain dependent on wild-type BRD4, which supports transcription and cell proliferation in a BRD-independent manner (Shu et al., 2016). Another group of small molecule-based proteolysis-targeting chimeras (PROTACs), which include dBET1 (Winter et al., 2015), dBET6 (Xu et al., 2018), ARV-825 (Lu et al., 2015), ARV-771 (Raina et al., 2016; Sun et al., 2018), BETd-246 (Bai L. et al., 2017), and MZ1 (Noblejas-López et al., 2019), can inhibit the expressions of target proteins via proteasomal degradation. BETd-246 exerted strong effects on growth inhibition and apoptosis and was more effective than its parental compound (BETi-211) in TNBC cells. In addition, MZ1 also exhibits anti-proliferation activity in JQ1-resistant cells.

### Histone Methyltransferase Inhibitors

Methylation catalyzed by HMTs generally involves S-adenosylmethionine (SAM) as the methyl donor. In 2007, Tan et al. first discovered that S-adenosylhomocysteine hydrolase inhibitor 3-Deazaneplanocin A (DZNep) caused the degradation of PRC2 components: SUZ12, EZH2, and EED which was accompanied by a decreased in H3K27me3. Moreover, they showed the anti-cancer function of DZNep in inducing cell death in BC cells by re-expressing PRC2-repressed genes, such as *FBXO32, LAMB3*, *PLAU*, *PPP1R15A*, *TGFBI*, *IGFBP3*, and *TNS3*; furthermore, *FBXO32* was associated with DZNep-induced apoptosis. Until now, the most widely developed HMTis are EZH2 inhibitors (EZHis), which include tazemetostat (EPZ-6438), CPI-0209, CPI-1205, GSK2816126, PF-06821497, and DS-3021. All of these inhibitors have been examined in clinical trials, although their roles in BC are not clear. In BC, EZH2is permit expression of TSGs, such as *FOSB*, *FOXC1*, *RUNX3*, *CDKN1C*, *CHD1*, and *TET1* that had been silenced by EZH2-mediated H3K27me3 in the canonical role. Expression of these genes leads to suppressed proliferation, less invasion, and greater response to adriamycin-based treatment (Song et al., 2016; Hirukawa et al., 2018; Yu Y. et al., 2019; Zhang et al., 2020). In addition, independent of the canonical role of PRC2 or the catalytic function of EZH2, EZH2 also activate oncogenes in BC, such as *Cyclin D1*, *c-Myc*, *NOTCH1*, *IL-6*, *IL-8*, *IL-11*, *TNF*, *CXCR4*, *CXCL18*, *MMP2*, and *MMP7* (Shi et al., 2007; Lee et al., 2011; Hartman et al., 2013; Gonzalez et al., 2014; Mahara et al., 2016; Li et al., 2017; Huang et al., 2018; Jiang et al., 2019). Therefore, EZH2is are insufficient to inhibit cell growth and induce apoptosis in BC cells, whereas silencing of EZH2 by RNA interference effectively suppress tumor growth. The recently discovered EZH2 selective degrader, MS1934, can effectively degrade it at the protein level and kill TNBC cells which helps overcome the limitations of EZHis (Ma et al., 2020). Relative to *BRCA1*-proficient tumors, EZH2 is upregulated in *BRCA1*-dificient tumors which are dependent on EZH2 and more sensitive to DZNep (Puppe et al., 2009). Moreover, the combination of GSK126 and cisplatin produces a greater antitumor effect than monotherapy in *BRCA1*-deficient tumors (Puppe et al., 2019). However, EZH2 acts in conjunction with other epigenetic regulators, and the combined treatment is more effective than monotherapy. For example, the TSG *CDKN1C* is suppressed by EZH2-mediated H3K27me3 and histone deacetylation, which is robustly reversed by the combination DZNep with TSA. Furthermore, a triple combination of 5-aza-2′-deoxycytidine (AZA), DZNep and TSA can further enhance the level of *CDKN1C* which may be due to AZA-induced reduction of H3K9me2 in a DNA methylation independent manner (Yang et al., 2009). More importantly, many gene sets are identified to be regulated by coordinated functions of EZH2 with HDAC and/or DNA methylation. In BC, it implicated the mechanistic heterogeneity implying that the tumor antigen *GAGEs* can be regulated by different epigenetic mechanisms in cell context-dependent manner (Sun et al., 2009). In HER2-positive BC cell line, combined treatment of structural analogue 3-deazaadenosine (DZA) and trastuzumab shows synergistic growth inhibition (Hayden et al., 2011). Furthermore, dual-target inhibitors toward both EZH2 and EHMT2 are being developed, including HKMTI-1-005, HKMTI-1-022, and HKMTI-1-011, which are more effective at re-expressing aberrantly silenced genes and BC cell growth compared to single target inhibitors (Curry et al., 2015). Numerous dietary chemo-preventive agents exhibit potent anti-cancer abilities (Supplementary Table 4). However, the mechanisms underlying EZH2 degradation and modulation of its target proteins remain unclear.

Binding modes can be used to divide G9a inhibitors into three groups. The first group contains substrate competitive inhibitors (BIX01294, UNC0224, UNC0321, UNC0638, UNC0646, UNC0631, UNC0642, E72, A-336, HKMTI-1-247, HKMTI-1-248, and CM-272). The second group includes SAM cofactor competitive inhibitors (BIX01338, BRD9539, BRD4770, CBC-12, chaetocin, and sinefungin). The third group includes inhibitors with unknown mechanism of action (DCG066, CPUY074001, CPUY074020, TM2-115, 867750, and 867751). As a substrate competitive inhibitor, BIX-01249 induces autophagy and increases intracellular ROS concentrations, which sensitizes BC cells to TRAIL by upregulating the expression of the ATF4/CHOP-dependent death receptor (DR5) (Kim et al., 2013, 2018). As another substrate competitive inhibitor, UNC0638 blocks CSC properties and the EMT by targeting the SNAIL-G9a axis and restoring E-cadherin in TNBC cells, which decreases their invasiveness and motility (Liu X. R. et al., 2018). Unfortunately, most studies of HMTis have examined their effects at the cellular level, and both *in vivo* experiments and clinical studies are needed to determine whether these drugs have clinical values.

Protein arginine methyltransferase inhibitors (PRMTis) include arginine competitive inhibitors (MS023, GSK3368715, GSK3359088, TP-064, EPZ020411, GSK591, GSK3235025, GSK3326595, and MS117), SAM cofactor competitive inhibitors (AMI-1, JNJ-64619178, LLY-283, and SGC3027), bi-substrate competitive inhibitors (SKI-72 and SGC8158), and allosteric inhibitors (SGC707, SGC6870, and Compound 1a) (Wu et al., 2021). In 2004, the first generation PRMTis AMI-1 was identified by high-throughput screen, which inhibited the nuclear receptor-mediated transactivation from ERE in MCF-7 cells (Cheng et al., 2004). Furthermore, AMI-1 promotes the sensitivity of BC cells to cetuximab and adriamycin (Li et al., 2016; Nakai et al., 2018). Some PRMTis inhibit proliferation of BC cells, such as MS023 (Eram et al., 2016), DCLX069, DCLX078 (Xie et al., 2014), and LLY-283 (Bonday et al., 2018). A newly synthesized PRMT4-specific inhibitor SKI-73 effectively inhibited BC cell invasion by changing epigenetic plasticity and inhibiting the invasion-prone subpopulation (Cai et al., 2019). However, these proven effects need to be further validated *in vitro*. The allosteric inhibitors may be more promising, which inactivate PRMTs by altering their conformation and show better selectivity. Moreover, different allosteric inhibitors can be designed for the same enzyme based on diverse binding sites. Therefore, their roles in BC should be further studied.

### Lysine Demethylase Inhibitors

The most widely studied lysine demethylase inhibitors (KDMis) are LSD1is, which can be classified as irreversible or reversible inhibitors (Supplementary Table 4). Irreversible inhibitors include tranylcypromine (TCP), pargyline, phenelzine, ORY-1001, GSK2879552, T5342129, bizine, IMG-7892, INCB059872, and ORY-2001 (vafidemstat). Reversible inhibitors have been discovered via high-throughput screening and hit-to-lead optimization, and include SP2509, CC9001, and GSK-690. The monoamine oxidase inhibitor TCP was initially approved by the FDA for treating mood and anxiety disorders, although it is recognized as an LSD1i. In BC, TCP attenuates tumor growth and metastasis by permitting expression of *E-cadherin* and other epithelial marker via disruption of the interaction between LSD1 and Slug (Ferrari-Amorotti et al., 2014). In TNBC, phenelzine enhances the effect of immunotherapy by reducing nuclear demethylase activity and increasing the transcription and expression of M1-like markers, which promote the anti-tumor M1-like phenotype among macrophages (Tan et al., 2019). Compared with monotherapy, a combination of pargyline with SAHA and CtBP inhibitors provided synergistic anti-cancer effects in BC (Vasilatos et al., 2013; Byun et al., 2019). Many natural products have also been identified to block LSD1 activity, such as isoquercitrin (Xu et al., 2019), cryptotanshinone (Wu et al., 2012), flavones (Han et al., 2018a,b), resveratrol (Abdulla et al., 2013), baicalin (Lomenick et al., 2011), and geranylgeranoic acid (Sakane et al., 2014). Isoquercitrin induces the expression of key proteins in the mitochondrial-mediated apoptosis pathway and causes apoptosis in LSD1-overexpressed MDA-MB-231 cells via inhibition of LSD1.

Another group of KDMis contains JmjC inhibitors, which are mainly 2-oxoglutarate competitors and include hydroxamic acid derivatives, hydroxyquinoline derivatives, metal-chelating inhibitors, conjugated arylalkenes, cyclic peptides, metal-containing inhibitors, and inhibitors targeting JmjC-KDM non-catalytic domains. In BC, these inhibitors can kill cancer cells and exert synergistic effects with other anti-cancer drugs, such as trastuzumab (Gale et al., 2016), lapatinib (Paroni et al., 2019), and 5-aza-2′-deoxycytidine (Leadem et al., 2018), which can help overcome treatment resistance.

### Combined Treatment

As described above, epi-drugs show robust anti-tumor effects in BC by interrupting in diverse oncogenic pathways. The combination of multiple epi-drugs among themselves or with chemotherapy, endocrinotherapy, immunotherapy, and radiotherapy have emerged as new tools to enhance anti-tumor efficacy or reverse primary or acquired drug resistance. Epi-drugs synergize with DNA-damaging [epirubicin (Marchion et al., 2005), doxorubicin (Oh et al., 2010; Fatima et al., 2019), cisplatin (Wawruszak et al., 2015; Pérez-Peña et al., 2016; Puppe et al., 2019), and carboplatin (Pérez-Peña et al., 2016)] and anti-mitotic [toxal (Shi et al., 2010), docetaxel (Pérez-Peña et al., 2016), and paclitaxel (Bonezzi et al., 2012; Lai et al., 2020)]. For example, SAHA can enhance de-condensation of chromatin to facilitate DNA access to epirubicin, and result in increased DNA damage and cell death (Marchion et al., 2004). Interestingly, paclitaxel also induces BC stem cell enrichment via KDM6A-mediated epigenetic activation of pluripotency genes, such as *NANOG*, *SOX2*, and *KLF4*. However, pharmacological inhibition of KDM6A by GSKJ4, a H3K27me3 demethylase inhibitor toward JMJD3 and UTX, overcomes the paclitaxel-induced BC stem cell enrichment (Yan et al., 2017; Lu H. et al., 2020).

Tamoxifen resistance is a challenge in treatment of ER-positive BC, and only 20% of resistant patients are sensitive to fulvestrant and AIs. HDACis (SAHA, TSA, and VPA) display synergetic action with AIs tamoxifen or letrozole in ER-positive tamoxifen-sensitive BC cells and overcome resistance in tamoxifen-resistant cells. Importantly, VPA can antagonize the drawback of tamoxifen in promoting proliferation of uterine endometrial cells (Hodges-Gallagher et al., 2007; Lee et al., 2012). However, in ER-negative BC cells, VPA and LBH589 restored the expression of ERα and overcame the primary resistance to antiestrogen therapy (Zhou et al., 2007; Fortunati et al., 2010). BRD3/4 can recruit WHSC1 to activate *ER*α by catalyzing H3K36me2/3. Combination of JQ1 with fulvestrant effectively reversed tamoxifen resistance and inhibited tumor growth of tamoxifen-resistance MCF7 xenografts (Feng et al., 2014). Moreover, Ent can also sensitize ER-negative BC to letrozole by re-expressing ERα and aromatase, and the combination of Ent with letrozole inhibit tumor growth and metastasis to lung in xenografts (Sabnis et al., 2011). A phase II clinical trial has also confirmed that a combination of Ent and exemestane was safe and effective in terms of prolonged survival among postmenopausal women with locally advanced or metastatic ER-positive BC, which might be related to increased protein lysine acetylation in the patients’ peripheral blood cells (Yardley et al., 2013).

In HER2-positive BC, trastuzumab alone or in combination with chemotherapy are effective treatments; however, resistance is common. Treatment using Ent can overcome both trastuzumab and lapatinib resistance, and a clinical trial revealed that polytherapy using Ent, trastuzumab, and lapatinib was safe and effective (Huang et al., 2009; Lee et al., 2014; Lim et al., 2019). Both VPA and SAHA can enhance trastuzumab-mediated antibody-dependent cell-mediated phagocytosis and apoptosis, as well as induce immunogenic cell death (Laengle et al., 2020). Sustained treatment with lapatinib induce BC cells kinome reprogramming by reactivating ERBB2/ERBB3 signaling and activating tyrosine kinases. Combination of JQ1 and lapatinib induce enhanced apoptosis in lapatinib-resistant cells. JQ1 inhibited the expression of kinases which are involved in lapatinib resistance by inhibiting BRD4 localization to the promoters and enhancers of lapatinib-response genes and reducing the accumulation of phosphorylated Ser2 RNAPII at the promoters (Stuhlmiller et al., 2015). Specific pharmacological inhibition of KDM5A by YUKA1 or KDM5-inh1 significantly reduces resistance to trastuzumab and lapatinib in HER2-positive BC (Gale et al., 2016; Paroni et al., 2019).

Triple-negative breast cancer is resistant to numerous chemotherapy agents, and no targeted drugs against it are available. PARPi is a promising agent to treat BC with defects in DNA repair by HR in a synthetic lethal approach, such as *BRCA*-mutated or *PTEN* loss in TNBC cells. However, the antagonism mediated by DNA repair-associated enzymes leads to resistance to PARPis, which calls for new strategies. Combination of SAHA or BETis (JQ1, I-BET762, or OTX015) with PARPi (olaparib) shows synergistic effects. Interestingly, PTEN turned out to be a favorable factor to the combination of SAHA with olaparib in TNBC which promoted both apoptosis and autophagy to inhibit proliferation and accumulated DNA damage, while downregulation of PTEN was unfavorable (Min et al., 2015). JQ1 also sensitized the HR-proficient BC to PARPi by prohibiting HR and facilitating PARPi-mediated DNA damage. JQ1 epigenetically silenced two HR-associated genes *BRCA1* and *RAD51*. These studies suggest that combined use of epi-drugs and PARPi can improve the anti-tumor effect of PARPi in HR-proficient BC with loss *BRCA*-mutated or *PTEN* silencing (Yang et al., 2017).

Immune checkpoint inhibitors (ICI) have been recognized as novel tools for BC treatment. However, the response to ICIs is poor because tumors recruit myeloid-derived suppressor cells to inhibit activation and infiltration of T-cells. Addition of Ent to anti-PD-1 or anti-CTLA-4 reversed the resistance by improving the infiltration and function of immune cells. This combination significantly reduced the inhibition functions of myeloid-derived suppressor cells and increased CD8^+^ T cells (Christmas et al., 2018; McCaw et al., 2019).

Multiple epi-drugs approaches have synergistic effects in BC treatment. For example, JQ1 and SAHA as co-treatments strengthened the anti-cancer efficacy of SAHA by preventing the SAHA-induced re-expression of *LIFR* and activation of downstream JAK1/STAT3 pathway (Zeng et al., 2016). Combining JQ1 and HDACi mocetinostat effectively reduced BC cell viability by enhanced suppression of genes-associated with cell cycle progression and increased expression of *USP17* (Borbely et al., 2015).

In addition to medication, epi-drugs also show synergetic functions with radiotherapy. TIP60 is necessary for activating ATM kinase and γH2AX after the DNA double strand breaks. The combination of TH1834 (a TIP60 inhibitor) pre-treatment and ionizing radiation induced increased γH2AX expression in MCF7 BC cells, while caused reduction in MCF10A cells (Gao et al., 2014). Furthermore, a HDACi TMU-35435 also enhanced radiosensitivity by causing misfolded protein aggregation and autophagy (Chiu et al., 2019). Moreover, a study on BC patients with brain metastasis also revealed that VPA promoted the effects of whole brain radiotherapy and prolonged survival by 6 months (Reddy et al., 2015).

Due to the complexity of crosstalk among HMs and the synergistic anti-cancer effect of available drugs, more potent dual-target inhibitors have been developed. For example, MC3324 (a dual-KDM inhibitor against LSD1 and UTX) up-regulates H3K4me2 and H3K27me3, which caused growth arrest and apoptosis in both hormone-responsive and insensitive BC cells by inhibiting ERα signaling (Benedetti et al., 2019). A dual-acting inhibitor of ER and HDAC has been designed, which exhibits anti-ERα and HDACi activities and exerts more potent anti-cancer effects compared with those of tamoxifen (Tang et al., 2015).

### Epi-Drugs in Clinical Practice

The promising preclinical results provide a solid foundation for translating epi-drugs into clinical trials for BC treatment. We have summarized the clinical trials of epi-drugs in treating BC (mainly from https://www.clinicaltrials.gov/) in Supplementary Table 3. There are numerous completed (NCT00262834, NCT00777049, and NCT01171924) and ongoing (NCT00416130, NCT01638533, and NCT04676516) clinical trials to estimate the safety, pharmacokinetics and pharmacodynamics to find optimal doses and schedules of epi-drugs monotherapies. Oral SAHA at 300 or 400 mg dose, twice daily for 14 days with a 7-day rest in between treatments has been proven to be tolerable (Vansteenkiste et al., 2008). But only two BC patients were included in this study, which was too small to draw the accurate response rate. Other ongoing trials will estimate the optimal dose of SAHA. In addition to monotherapy, epi-drugs are more commonly used in combination regimens in clinical use. For example, a phase II study of SAHA combined with tamoxifen to treat hormone therapy-resistant BC showed an objective response rate of 19% and clinical benefits rate of 40% (Munster et al., 2011). Furthermore, Ent was combined with exemestane, which improved the OS from 19.8 months with exemestane monotherapy to 28.1 months (Yardley et al., 2013). Recently in a phase II study, SAHA was used to improve the immunotherapy response in ER-positive BC by combining it with tamoxifen and immunotherapy agent pembrolizumab. This polytherapy regimen so far obtained an ORR of 4% and clinical benefits rate of 19% (Terranova-Barberio et al., 2020). More importantly, another phase II trial (NCT04190056) is ongoing to further clear the role of this combined regimen in triggering immune response to treat ER-positive BC, and reduce the dosage and side effects. In preclinical trials, both SAHA and BETis have shown synergistic effects with olaparib in BC treatment (Min et al., 2015; Yang et al., 2017). Two ongoing trials (NCT03901469 and NCT03742245) are investigating the efficacy and safety of epi-drugs combination with PARPis by inhibiting DNA damage repair. In summary, the aforementioned clinical trials add to evidence that epi-drugs are effective treatment for BC and needs to be further explored.

### Treatment-Related Adverse Events and Dose-Limiting Toxicities (DLTs)

To some context, the application of epi-drugs in BC patients is limited by the side effects, which lead to adverse events (AEs) and dose-limiting toxicities (DLTs). AEs refer to side effects that occur during treatment and can be classified as dose-related toxicities and other problems. In an early phase II trial of SAHA monotherapy in BC, colorectal, and non-small cell lung cancer, drug-related AEs are similar to those of traditional chemotherapy, including anorexia, fatigue, nausea, diarrhea, vomiting, thrombocytopenia, weight loss, asthenia, anemia, constipation, stomatitis, cancer pain, dry mouth, dyspepsia, upper abdominal pain, and vertigo. Grade 3/4 AEs included thrombocytopenia, anemia, asthenia, and nausea (Vansteenkiste et al., 2008). Relative to traditional chemotherapy or endocrine therapy, addition of SAHA in the regimen only increased the incidence of diarrhea (Ramaswamy et al., 2012; Tu et al., 2014; Terranova-Barberio et al., 2020). In another trial, addition of SAHA also caused neutropenia, lymphopenia, alopecia, and the most dangerous AE, pulmonary emboli (Munster et al., 2011). Therefore, we urgently need to study how to overcome these AEs, and finally achieve optimal regimens.

## Conclusion

Substantial proteomic and genomic analyses have revealed that BC involves numerous molecular alterations. Although specific genetic alterations are known to drive BC, epigenetic pathways also play important roles in its oncogenesis. Furthermore, alterations in HMs may be used as biomarkers for predicting BC aggressiveness and the effectiveness of therapies that target epigenetic mechanisms. Additional research is needed to confirm whether correlations between early epigenetic changes and clinical characteristics can guide strategies to achieve better clinical outcomes.

Unfortunately, there are numerous barriers to clarifying the roles and mechanisms of HMs in BC. First, HMs are dynamic processes that occur within and between nucleosomes (Eissenberg and Shilatifard, 2010). Second, enzymes that catalyze HMs can also catalyze non-histone modifications that affect p53, Rb, and Myc (Singh et al., 2010). Third, HMs can create independent, competitive, or synergistic effects via crosstalk (Lee et al., 2010). Fourth, HM modifiers are generally located in large multi-protein complexes, such as the PRC2, LSD1/NuRD, MLL/COMPASS, and HDAC complexes (Cao et al., 2002; Lee et al., 2005; Wang et al., 2007, 2009; Shilatifard, 2012; Yang et al., 2018). Finally, the same writer/reader combination can have very different biological effects depending on the contexts, which complicates efforts to target these molecules (Patel and Wang, 2013). Therefore, additional studies are needed to clarify the roles of HMs in BC progression, including the contributions of specific modifiers, targets, modes of action, and the tumor microenvironment. These issues also highlight the difficulty in attempting to precisely exploit these mechanisms, and related drugs must be used with caution. For example, pharmacological inhibition of HDAC11 decreases lymph node tumor growth, but also enhances metastasis from the lymph node to distant sites (Leslie et al., 2019). In contexts where HM modifiers influence non-canonical pathways, specific degraders may be useful for controlling related enzymes at the protein level, and thus overcoming the limitations of specific inhibitor drugs (Bai L. et al., 2017; Noblejas-López et al., 2019; Ma et al., 2020).

Reducing the AEs of epi-drugs is a challenging issue in the clinical setting. Treatment-related AEs and DLTs are generally due to various absorptivity, penetrability, half-life, properties, and the complex interactions between different drugs in the human metabolism. Carefully designed combinations of epi-drugs and traditional anti-cancer drugs or synthesized multi-target drugs may produce better therapeutic effects, with greater ability to overcome drug resistance and fewer AEs (Stratikopoulos et al., 2015; Qiao et al., 2020). Relative to synthetic compounds, bioactive natural compounds have powerful anticancer properties, which can involve regulating the epigenetic mechanisms of transformed cells (Xu et al., 2019). However, there are limited data regarding the use of epigenetic regulatory phytochemicals to target human BC. Further studies are needed to provide additional pre-clinical data that may guide the development of epigenetic regulators from the bench to clinical applications.

In conclusion, there is still substantial work needed to clarify the mechanisms of HMs and exploit these mechanisms to develop appropriate drugs and treatment regimens. Epi-drugs have achieved remarkable effects in terms of precise and effective personalized treatment for BC. Thus, it is reasonable to hope that more precise, specific, and effective drugs will be developed to target HMs as a strategy for treating BC.

## Author Contributions

DP and SX conceived the idea and designed the study. WL was the first author, retrieved and analyzed the data and drafted the manuscript. HW, SS, and QW revised and polished the manuscript. All authors read and approved the final manuscript.

## Conflict of Interest

The authors declare that the research was conducted in the absence of any commercial or financial relationships that could be construed as a potential conflict of interest.

## Publisher’s Note

All claims expressed in this article are solely those of the authors and do not necessarily represent those of their affiliated organizations, or those of the publisher, the editors and the reviewers. Any product that may be evaluated in this article, or claim that may be made by its manufacturer, is not guaranteed or endorsed by the publisher.

## Abbreviations

53BP1: TP53-binding protein 1
5-FU: 5-fluorouracil
ADAM9: a disintegrin and metalloproteinase domain 9
AE: adverse event
AI: aromatase inhibitor
AR: androgen receptor
ARE: AR-response elements
ARRDC3: arrestin domain-containing 3
ATF4: activating transcription factor 4
ATRA: all-trans retinoic acid
AZA: 5-aza-2′-deoxycytidine
BAP1: BRCA1 associated protein 1
BARD1: BRCA1-associated RING domain protein 1
BC: breast cancer
BCAT1: branched-chain amino acid transaminase 1
BET: bromodomain and extra terminal domain
BETi: BET inhibitor
BIRC3: baculoviral IAP repeat containing 3
BMI1: B lymphoma Mo-MLV insertion region 1
BNIP3: BCL2 interacting protein 3
BRCA1: breast and ovarian cancer susceptibility protein 1
BRD: bromodomain
CDH4: cadherin 4
CDH10: cadherin 10
CDKN1C: cyclin dependent kinase inhibitor 1C
CHD1: chromodomain helicase DNA binding protein 1
CHOP: C/EBP homologous protein
CoREST: RE1-silencing transcription factor corepressor
CSC: Cancer stem cell
CtBP: C terminal-binding protein
CTGF: connective tissue growth factor
CXCR4: C-X -C motif chemokine receptor 4
CXCL12: C-X -C motif chemokine ligand 12
CXCL18: C-X -C motif chemokine ligand 18
DCIS: ductal carcinoma *in situ*
DLT: dose-limiting toxicity
DR5: death receptor 5
DZA: 3-Deazaadenosine
DZNep: 3-Deazaneplanocin A
EGFR: epidermal growth factor receptor
EGR1: early growth response 1
EMA: European medicines agency
EMT: epithelial-mesenchymal transition
ENT: entinostat
Epi-drug: epigenetic drug
ER: estrogen receptor
ERE: estrogen response element
EZH2i: EZH2 inhibitor
FBXO32: F-BOX protein 32
FDA: US food and drug administration
FOXA1: forkhead box A1
FOXC1: forkhead box C1
FOXP1: forkhead box P1
G6PD: glucose-6-phosphate dehydrogenase
GATA-5: GATA binding protein-5
HAT: histone acetyltransferases
HATi: HAT inhibitor
HDAC: histone deacetylase
HDACi: HDAC inhibitor
HIC1: hypermethylated in cancer 1
HK2: hexokinase 2
HM: histone modification
HMT: histone methyltransferase
HMTi: HMT inhibitor
HOTAIR: HOX transcript antisense RNA
HR: homologous recombination
ICI: immune checkpoint inhibitor
IDC: invasive ductal carcinoma
IGFBP3: insulin like growth factor binding protein 3
IGFBP5: insulin like growth factor binding protein 5
IKBKE: inhibitor of nuclear factor kappa B kinase subunit epsilon
IL-6: interleukin-6
IL-8: interleukin-8
IL-11: interleukin-11
JmjC: JumonjiC
KDMi: lysine demethylase inhibitor
KISS1: KiSS-1 metastasis suppressor
KLF4: kruppel like factor 4
KMT: lysine methyltransferase
L1CAM: L1 cell adhesion molecule
LAMB3: laminin subunit beta 3
LDHA: lactate dehydrogenase A
LIFR: leukemia inhibitory factor receptor
LOXL2: lysyl oxidase like 2
LSD1i: LSD1 inhibitor
MMP2: matrix metallopeptidase 2
MMP7: matrix metallopeptidase 7
MMP9: matrix metallopeptidase 9
NANOG, NANOG: nanog homeobox
NCoR: nuclear receptor corepressor
NOTCH1: notch receptor 1
NuRD: nucleosome remodeling and histone deacetylation
OCT4: octamer binding transcription factor 4
O-GlcNAc: O-linkedβ-N -acetylglucosamine
OGT: O-linkedβ-N -acetylglucosamine transferase
ORR: objective response rate
PAD: peptidyl arginine deminase
PARP: poly ADP ribose polymerase
PARPi: PARP inhibitor
PAX3: paired Box 3
PDK1: pyruvate dehydrogenase kinase 1
PD-L1: programmed death-ligand 1
PR: progesterone receptor
PHF20L1: PHD finger protein 20 like 1
PLAU: plasminogen activator, urokinase
PPH3: phospho-histone H3
PPP1R15A: protein phosphatase 1 regulatory subunit 15A
PRC1: polycomb repressive complex 1
PRC2: polycomb repressive complex 2
PRDM: PRD-BF1 and RIZ homology domain
PRMT: protein arginine methyltransferase
PROTAC: proteolysis-targeting chimera
PTEN: phosphatase and tensin homolog
PTM: posttranslational modification
rDNA: ribosomal DNA
ROS: reactive oxygen species
rRNA: ribosomal RNA
S100A4: S100 calcium binding protein A4
SAM: S-adenosylmethionine
SET: Su(var)3-9, enhancer of zeste, trithorax
SFRP1: secreted frizzled related protein 1
SLC2A1: solute carrier family 2 member 1
SUMO: small ubiquitin-like modifier
TCP: tranylcypromine
TFF1: trefoil factor 1
TGFBI: transforming growth factor beta induced
TIGAR: TP53-induced glycolysis regulatory phosphatase
TNBC: triple-negative breast cancer
TNF: tumor necrosis factor
TNS3: tensin 3
TRAIL: TNF-related apoptosis-inducing ligand
TRIM37: tripartite motif containing 37
TSA: trichostatin A
TSG: tumor suppressor gene
USP16: ubiquitin specific peptidase 16
VPA: valproic acid
WDR5: WD repeat domain 5
WNT5A: Wnt family member 5A
YB-1: Y-box binding protein-1
ZEB1: Zinc finger E-box binding homeobox 1
ZEB2: Zinc finger E-box binding homeobox 2.
