# Supplementary material for: Targeting Histone Modifications in Breast Cancer: A Precise Weapon on the Way
Source: Front Cell Dev Biol. 2021 Sep 14;9:736935. doi: 10.3389/fcell.2021.736935 (PMC8476812; doi:10.3389/fcell.2021.736935)
Supplement: Supplementary file 1 [file Data_Sheet_1.docx]

**Supplemental Information**

**Table S1. Functions of histone modifiers in BC**

| Modifiers | Cooperators | Targets | Substrates | Functions | Refs. |
| --- | --- | --- | --- | --- | --- |
| HATs | | | | | |
| CBP | AGAP2-AS1 | ↑MyD88 | H3K27ac | ↑Proliferation  ↑Trastuzumab resistance  ↓Apoptosis | (Dong et al., 2018) |
| CBP | MTDH | ↑TWIST1 | H3pan-ac | ↑CSCs | (Liang et al., 2015) |
| CBP | EYA1 | ↑Cyclin D1 | H3K9ac | ↑Proliferation | (Wu et al., 2013) |
| CBP | Unknown | ↑TINCR | H3K27ac | ↑Trastuzumab resistance  ↑EMT | (Dong et al., 2019) |
| P300 | CGI-99 | ↑IL-6 | H3K9ac H3K14ac H3K27ac  H4K5ac  H4K8ac  H4K12ac | ↑CSCs  ↑Metastasis | (Lin et al., 2017) |
| P300 | MRTF-A | ↑MYH9  ↑MYL9  ↑CYR61 | H3K9ac | ↑Migration | (He et al., 2015) |
| P300 | HBXIP | ↑MDM2 | H3pan-ac | ↑Proliferation | (Li et al., 2015) |
| P300 | DOT1L  c-Myc | ↑SNAIL  ↑ZEB1  ↑ZEB2 | H3pan-ac  H3K79me2 | ↑CSCs | (Cho et al., 2015) |
| P300 | CBX7 | ↑DKK-1 | H3pan-ac | ↑CSCs | (Kim et al., 2015) |
| P300 | ER  MLL1  MLL3 | ↑HOTAIR | Hpan-ac | ↓Apoptosis | (Bhan et al., 2013) |
| P300/CBP | CapG | ↑PIK3R1/P50 | H3K27ac | ↑Paclitaxel resistance | (Chi et al., 2019) |
| GCN5 | SND1 | ↑SMAD2/3/4 | H3K9ac | ↑Metastasis | (Yu et al., 2017) |
| PCAF  P300 | MyoD  c-myb | ↑BRCA1 | H3pan-ac  H4pan-ac | ↑Cell viability | (Jin et al., 2011) |
| PCAF  GCN5 | Unknown | ↑MDR1 | H3K9ac | ↑Drug resistance | (Toth et al., 2012) |
| HBO1 | SIX1 | ↑HK2  ↑ALDOA  ↑PGK1  ↑ENO1  ↑LDHA | H4K5ac | ↑Proliferation | (Li et al., 2018) |
| MYST3 | Unknown | ↑ER | H3K9ac | ↑Proliferation | (Yu et al., 2017) |
| HDACs | | | | | |
| HDAC1 | CHD4 | ↓P21 | H3K9ac  H3K14ac | ↑Proliferation  ↑Colony formation | (Hou et al., 2017) |
| HDAC1 | ZNF750  KDM1A | ↓LAMB3  ↓CTNNAL1 | H3pan-ac  H3K4me1/2/3 | ↓Invasion  ↓Migration | (Cassandri et al., 2020) |
| HDAC1 | KLF9 | ↓MMP9 | H3K27ac | ↓Metastasis | (Bai et al., 2018) |
| HDAC1 | BRMS1L | ↓FZD10 | H3K9ac | ↓Metastasis | (Gong et al., 2014) |
| HDAC1 | TIEG1 | ↓EGFR | H3pan-ac | ↓Invasion | (Jin et al., 2012) |
| HDAC1 | UHRF1 | ↓MDR1 | H3pan-ac H4pan-ac | ↓Doxorubicin  resistance  ↓Vinblastine resistance | (Jin et al., 2010) |
| HDAC1 | SMAR1 | ↓Cyclin D1 | H3K9ac  H4K8ac | ↓Proliferation | (Rampalli et al., 2005) |
| HDAC2 | TRPS1 | ↓AES  ↓Casp7  ↓PERP | H4K16ac | ↑Proliferation | (Wang et al., 2018) |
| HDAC2 | PELP1 | ↓miR-200a  ↓miR-141 | H3K9ac | ↑Metastasis | (Roy et al., 2014) |
| HDAC2 | KLF-4 HDAC3 | ↓VEGF | Unknown | ↓Migration | (Ray et al., 2013) |
| HDAC2 | FOXO3a | ↓VEGF | H3pan-ac  H4pan-ac | ↓Angiogenesis | (Karadedou et al., 2012) |
| HDAC3 | Unknown | ↓CDKN1A | H3K9ac  H4K16ac | ↑Proliferation | (Yu et al., 2020) |
| HDAC3 | FOXF2 | ↓FOXQ1 | H3K9ac H3K27ac | ↑Migration  ↑Invasion  ↑Multidrug resistance | (Kang et al., 2019) |
| HDAC3 | Unknown | ↓miR-31 | H3K9ac | ↑Proliferation  ↑Aerobic glycolysis | (Zhao et al., 2018) |
| HDAC3 | Unknown | ↓ANCR | H3pan-ac  H4pan-ac | ↑Migration | (Li et al., 2017) |
| HDAC4 | Unknown | ↓SMAD4 | H3pan-ac | ↑5-FU resistance | (Yu et al., 2013) |
| HDAC7 | Unknown | ↓IL-24 | H3K27ac | ↑Proliferation  ↑Invasion  ↑CSCs | (Cutano et al., 2019) |
| HDAC8 | SMAD3/4 | ↓SIRT7 | H4pan-ac | ↑Metastasis  ↑Paclitaxel resistance | (Tang et al., 2020) |
| SIRT1 | Unknown | ↓SFRP1  ↓E-cadherin ↓GATA-5 | H3K9ac  H4K16ac | ↑Viability | (Pruitt et al., 2006) |
| SIRT1 | Unknown | ↓Survivin | H3K9ac | ↓Tumor growth | (Wang et al., 2008) |
| SIRT2 | Unknown | ↓ARRDC3 | H4K16ac | ↑Migration | (Soung et al., 2014) |
| HMTs | | | | | |
| MLL2 | ERα | ↑IL-20 | H3K4me1/2 | ↑Proliferation | (Su et al., 2016) |
| MLL2 | GCN5  FOXOs | ↑c-Myc | H3K4me3  H3pan-ac | ↑Lapatinib resistance | (Matkar et al., 2015) |
| MLL2 | LSD1  UTX  ASXL2 | ↑NCOA3  ↑BMP7  ↑CA4  ↑RSP6KB1 | H3K4me3  H3K9me2  H3K27me3 | ↑Proliferation | (Park et al., 2016) |
| MLL3 | Unknown | ↑AGR3  ↑PGR  ↑CA2 | H3K4me1 | ↑Proliferation | (Gala et al., 2018) |
| MLL3 | SET1A | ↑ESR1 | H3K4me1  H3K4me3 | ↑Proliferation | (Kim et al., 2020) |
| MLL3 | FOXA1  ER | ↑TFF1  ↑PGR  ↑MYC | H3K4me1 | ↑Proliferation | (Jozwik et al., 2016) |
| MLL3 | ER  P300/CBP | ↑HOXB9 | H3K4me3  Hpan-ac | ↑Proliferation | (Deb et al., 2016) |
| SETD1A | Unknown | ↑SKP2 | H3K4me3 | ↑Proliferation  ↓Senescence | (Tajima et al., 2019) |
| SETD1A | Unknown | ↑MMPs | H3K4me3 | ↑Invasion  ↑Migration | (Salz et al., 2015) |
| SET7 | GATA1 | ↑VEGF | H3K4me1 | ↑Vascular endothelial cell proliferation  ↑Migration  ↑Tube formation | (Zhang et al., 2016) |
| SMYD3 | SMAD3 | ↑SNAIL1 | H3K4me3 | ↑EMT | (Fenizia et al., 2019) |
| SMYD3 | MRTF-A | ↑MYL9 | H3K4me2/3 | ↑Migration | (Luo et al., 2014) |
| NSD2 | Unknown | ↑TIGAR  ↑HK2  ↑G6PD | H3K36me2 | ↑Tamoxifen resistance | (Wang et al., 2016) |
| NSD2 | Unknown | ↑ADAM9  ↑EGFR | H3K36me2/3 | ↑Proliferation  ↑Invasion  ↑Gefitinib resistance | (Wang et al., 2019) |
| DOT1L | Unknown | ↑BCAT1 | H3K79me2 | ↑Migration  ↑Sphere formation | (Oktyabri et al., 2016) |
| G9a | EZH2  CDYL2 | ↓miR124 | H3K9me2  H3K27me3 | ↑Invasion  ↑Migration  ↑EMT | (Siouda et al., 2020) |
| G9a | Unknown | ↓CDH10 | H3K9me2 | ↑EMT | (Casciello et al., 2020) |
| G9a | TBX2  HP1  EGR1 | ↓NDRG1 | H3K9me2/3 | ↑Proliferation | (Crawford et al., 2019) |
| G9a | MYC | ↓CDKN1A  ↓GADD45A  ↓HMOX1  ↓VAMP4 | H3K9me2 | ↑Proliferation | (Tu et al., 2018) |
| G9a | E4BP  SUV39H1 | ↓RASSF8 | H3K9me2/3 | ↑Proliferation  ↓Apoptosis | (Karthik et al., 2018) |
| G9a | HDAC1  YY1 | ↓Hephaestin | H3K9me2 | ↑Proliferation | (Wang et al., 2017) |
| G9a | Unknown | ↓ARNTL ↓CEACAM7  ↓GATA2  ↓HHEX  ↓KLRG1  ↓OGN | H3K9me2 | ↑Proliferation  ↑Migration | (Casciello et al., 2017) |
| G9a | STAT3 | ↓miR-200c | H3K9me2 | ↑EMT  ↑CSCs | (Chang et al., 2015) |
| G9a | Unknown | ↓LC3-II  ↓GFP-LC3-II  ↓GFP | H3K9me2 | ↓Autophagy | (Kim et al., 2013) |
| G9a | SNAIL  DNMT1 | ↓FBP1 | H3K9me2 | ↑CSCs | (Dong et al., 2013) |
| G9a | SNAIL  DNMT | ↓E-cadherin | H3K9me2 | ↑Invasion  ↑Migration  ↑EMT | (Dong et al., 2012) |
| G9a | Unknown | ↓Beclin-1 | H3K9me2 | ↓Autophagy | (Park et al., 2016) |
| SETDB1 | SMAD3 | ↓SNAIL1 | H3K9me3 | ↓Invasion  ↓Camptothecin resistance ↓Doxorubicin resistance  ↓EMT | (Du et al., 2018) |
| SUV39H1 | SNAIL | ↓E-cadherin | H3K9me3 | ↑Invasion  ↑Migration  ↑EMT | (Dong et al., 2013) |
| SET8 | TWIST | ↓E-cadherin  ↑N-cadherin | H4K20me1 | ↑EMT  ↑Invasion | (Yang et al., 2012) |
| SUV39H2 | Unknown | ↓Tensin-3 | H4K20me3 | ↓Invasion  ↓Migration | (Shinchi et al., 2015) |
| SUV39H2 | Unknown | ↓EGR1  ↓CTGF | H4K20me3 | ↓Invasion  ↓Migration  ↓EMT | (Wu et al., 2019) |
| EZH2 | Unknown | ↓RAD51 | H3K27me3 | ↓HR repair  ↑Breast tumor initiating cells expansion | (Zeidler et al., 2005; Chang et al., 2011) |
| EZH2 | Unknown | ↓FOXO3 | H3K27me3 | ↑Proliferation | (Gong et al., 2016) |
| EZH2 | Unknown | ↓ERα | H3K27me3 | ↑Tamoxifen resistance | (Nie et al., 2019) |
| EZH2 | Unknown | ↓GATA3 | H3K27me3 | ↑Fulvestrant resistance  ↑Proliferation  ↑Invasion  ↑Migration | (Yomtoubian et al., 2020) |
| EZH2 | LncRNA UCA1 | ↓P21 | H3K27me3 | ↑Cell cycle progression ↑Tamoxifen resistance | (Li et al., 2019) |
| EZH2 | Unknown | ↓FOXC1 | H3K27me3 | ↑Invasion  ↑Migration | (Du et al., 2012; Hirukawa et al., 2018) |
| EZH2 | Unknown | ↓E-cadherin | H3K27me3 | ↑Invasion | (Cao et al., 2008) |
| EZH2 | SUZ12 | ↓RKIP | H3K27me3  H3K9me3 | ↑Invasion | (Ren et al., 2012) |
| EZH2 | Unknown | ↓miR-129-5p | H3K27me3 | ↑EMT  ↑Adriamycin resistance  ↑Vincristine resistance  ↑Paclitaxel resistance | (Luan et al., 2016) |
| EZH2 | Unknown | ↓RUNX3 | H3K27me3 | ↑Proliferation | (Fujii et al., 2008) |
| EZH2 | Unknown | ↓CIITA | H3K27me3 | ↓Tumor immunogenicity | (Truax et al., 2012) |
| EZH2 | Unknown | ↓KLF2 | H3K27me3 | ↑Proliferation | (Taniguchi et al., 2012) |
| EZH2 | Unknown | ↓BIK | H3K27me3 | ↓Apoptosis  ↑Paclitaxel resistance | (Si et al., 2016) |
| EZH2 | YAP | ↓GDF15 | H3K27me3 | ↑Migration | (Wang et al., 2018) |
| EZH2 | Unknown | ↓TIMP | H3K27me3 | ↑Invasion  ↑Migration | (Chien et al., 2018) |
| EZH2 | Unknown | ↓WWC1 | H3K27me3 | ↑Proliferation  ↑Migration | (Liu et al., 2018) |
| EZH2 | Unknown | ↓Period2 | H3K27me3 | ↑Invasion  ↑Colony formation  ↑Mammosphere formation | (Yu et al., 2018) |
| EZH2 | Unknown | ↓TET1 | H3K27me3 | ↑Proliferation  ↓Senescence | (Yu et al., 2019) |
| EZH2 | LINC00511 | ↓CDKN1B | H3K27me3 | ↑Proliferation | (Zhang et al., 2019) |
| EZH2 | DANCR | ↓SOCS3 | H3K27me3 | ↑Viability  ↑Invasion  ↑Migration | (Zhang et al., 2020) |
| EZH2 | LOXL1-AS1 | ↓miR-708-5p | H3K27me3 | ↑Invasion  ↑Migration | (Dong et al., 2020) |
| EZH2 | Unknown | ↓miR-381 | H3K27me3 | ↑Proliferation  ↑Cisplatin resistance | (Dou et al., 2019) |
| EZH2 | Unknown | ↓FOSB | H3K27me3 | ↑Proliferation | (Zhang et al., 2020) |
| EZH2 | YY1 | ↓PHACRT2-AS1 | H3K27me3 | ↑Proliferation  ↑Metastasis | (Chu et al., 2020) |
| EZH2 | SMYD2 | ↓SIAH1  ↓RASSF1  ↓AXIN2 | H3K27me3 | ↑Proliferation  ↑Invasion  ↑EMT | (Zeng et al., 2019) |
| EZH2 | DDX21 | ↓SNAIL | H3K27me3 | ↓EMT  ↓Invasion | (Zhang et al., 2018) |
| EZH2 | LINC01133 | ↓SOX4 | H3K27me3 | ↓Invasion  ↓Migration | (Song et al., 2019) |
| EZH2 | macroH2A1.2 | ↓LOX | H3K27me3 | ↓Bone metastasis | (Kim et al., 2018) |
| EZH2 | Unknown | ↓P21 | H3K27me3 | ↓Taxol sensitivity | (Mu et al., 2019) |
| PRMT1 | Unknown | ↑ZEB1 | H4R3me2as | ↑EMT  ↓Senescence | (Gao et al., 2016) |
| PRMT4 | Unknown | ↑E2F1 | H3R17me2 | ↑Proliferation | (El Messaoudi et al., 2006; Frietze et al., 2008) |
| PRMT5 | SET1 | ↑FOXP1 | H3R2me2s  H3K4me3 | ↑Proliferation  ↑CSCs | (Chiang et al., 2017) |
| PRMT5 | Unknown | ↑OCT4/A ↑KLF4  ↑c-Myc | H3R2me2s | ↑Doxorubicin resistance  ↑CSCs | (Wang et al., 2018) |
| PRMT5 | Unknown | ↓STC-1 | H4R3me2 | ↓Invasion  ↓Migration | (Huang et al., 2018) |
| PRMT6 | Unknown | ↓P21 | H3R2me2a | ↑Proliferation | (Phalke et al., 2012) |
| PRMT7 | HDAC3 | ↓E-cadherin | H4R3me2s  H3K4me3  H3pan-ac  H4pan-ac | ↑EMT  ↑Invasion  ↑Migration | (Yao et al., 2014) |
| KDMs | | | | | |
| LSD1 | Slug | ↓ESR1 | H3K4me2 | ↑Proliferation  ↑Invasion  ↑Migration | (Bai et al., 2017) |
| LSD1 | AR | ↓E-cadherin  ↑Vimentin | H3K4me2  H3K9me2 | ↑Proliferation  ↑Invasion  ↑Migration  ↑EMT | (Feng et al., 2017) |
| LSD1 | Unknown | ↓p21^Cif1/Waf^  ↓HNF4 ↓HoxA10  ↓FoxA2 | H3K4me2 | ↑CSCs | (Wu et al., 2013) |
| LSD1 | CARM1  USP7 | ↓E-cadherin  ↑Vimentin | H3K4me2  H3K9me2 | ↑Invasion | (Liu et al., 2020) |
| LSD1 | RORα | ↑CTNND1 | H3K9me2 | ↑Migration | (Kim et al., 2017) |
| LSD1 | Unknown | ↓TRIM37  ↑GATA3 | H3K4me2  H3K9me2 | ↓Invasion  ↓Migration | (Hu et al., 2019) |
| LSD1 | BRSM1 HDAC1/2  Co-REST | ↓Vimentin | H3K4me1/2  H3pan-ac | ↓Invasion  ↓Migration  ↓EMT | (Qiu et al., 2018) |
| LSD1 | Unknown | ↓PD-L1  ↓CCL5  ↓CXCL9 ↓CXCL10 | H3K4me2 | ↓Tumor immunogenicity | (Qin et al., 2019) |
| LSD1 | LSD1/NuRD  (MTA3) complex  SIX3 | ↓FOXC2  ↓WNT1 | H3K4me1/2  H3pan-ac | ↓Invasion  ↓EMT | (Zheng et al., 2018) |
| LSD1 | CtBP/LSD1  /CoREST complex  ZNF516 | ↓EGFR | H3K4me1/2  H3pan-ac | ↓Proliferation  ↓Invasion  ↓EMT | (Li et al., 2017) |
| KDM5A | Unknown | ↓P21  ↓BAK1 | H3K4me3 | ↑Proliferation  ↓Erlotinib sensitivity  ↓Apoptosis | (Hou et al., 2012) |
| KDM5B | Unknown | ↓HEXIM1 | H3K4me2/3 | ↑Proliferation  ↑Doxorubicin resistance  ↓Differentiation | (Montano et al., 2019) |
| KDM5B | Unknown | ↓SOX2  ↓NANOG | H3K4me3 | ↓CSCs  ↓Mammosphere formation | (Yeh et al., 2019) |
| KDM5B | EMSY | ↓miR-31 | H3K4me3 | ↑Invasion  ↑Migration  ↑Colony formation | (Viré et al., 2014) |
| KDM5B | TFAP2C  Myc | ↓CDKN1A | H3K4me3 | ↑Cell cycle | (Wong et al., 2012) |
| KDM5B | Unknown | ↓Let-7e | H3K4me3 | ↑Proliferation | (Mitra et al., 2011) |
| KDM5B | Unknown | ↓CAV1  ↓HOXA5  ↓BRCA1 | H3K4me3 | ↑Proliferation | (Yamane et al., 2007) |
| KDM5B/C | Unknown | ↓STING | H3K4me3 | ↓Immunogenicity | (Wu et al., 2018) |
| KDM5C | Unknown | ↓BRMS1 | H3K4me3 | ↑Invasion  ↑Migration | (Wang et al., 2015) |
| KDM2A | Unknown | ↓TET2 | H3K36me2/3 | ↑Invasion  ↑Migration | (Chen et al., 2017) |
| KDM2B | Unknown | ↓p15^INK4B^ ↓p16^INK4A^ ↓p57^KIP2^ | H3K4me3  H3K36me2 | ↑Proliferation | (Zheng et al., 2018) |
| KDM3A | ACK1 | ↑HOXA1 | H3K9me2 | ↑Proliferation  ↑Tamoxifen resistance | (Mahajan et al., 2014) |
| KDM3A | ER | ↑Ps2  ↑GREB1  ↑CCND1  ↑MYC  ↑XBP1 | H3K9me1/2 | ↑Proliferation  ↑Tamoxifen resistance | (Wade et al., 2015) |
| KDM3A | Unknown | ↑Cyclin D1 | H3K9me1/2 | ↑Proliferation | (Qin et al., 2017) |
| KDM3A | Unknown | ↑MYC  ↑PAX3 | H3K9me2 | ↑Migration  ↑Colony formation | (Zhao et al., 2016) |
| KDM3A | Unknown | ↑MMP9  ↑S100A  ↑JUN  ↓P53 | H3K9me2,  p53-K372me1 | ↑Invasion  ↓Apoptosis  ↑Cisplatin resistance  ↑Paclitaxel resistance | (Ramadoss et al., 2017) |
| KDM3A | Unknown | ↑BNIP3  ↑BNIP3L | H3K9me1/2 | ↑Anoikis | (Pedanou et al., 2016) |
| KDM3A | BRG1 | ↑MUC1 | H3K9me2 | ↑Invasion  ↑Migration | (Sun et al., 2019) |
| KDM4B | Unknown | ↑MYB  ↑MYC  ↑CCND1 | H3K9me3 | ↑Proliferation | (Kawazu et al., 2011) |
| KDM4B | Unknown | ↑LINE-1 | H3K9me3 | ↑DNA  damage | (Xiang et al., 2019) |
| KDM4B | ERα  HIF-1α | ↑CCNA1  ↑CCND1  ↑WEE1 | H3K9me3 | ↑Proliferation | (Yang et al., 2010) |
| KDM4C | HIF-1α | ↑BNIP3  ↑LDHA  ↑PDK1 ↑SLC2A1 ↑LOXL2 ↑L1CAM | H3K9me3 | ↑Tumor growth  ↑Metastasis | (Luo et al., 2012) |
| PHF8 | Unknown | ↑Cyclin A2 | H3K9me1 | ↑Proliferation | (Wang et al., 2016) |
| PHF8 | Unknown | ↑SNAI1 | H3K9me1/2 | ↑Proliferation  ↑EMT | (Shao et al., 2017) |
| UTX | MLL4 | ↑MMP9  ↑MMP11  ↑SIX1 | H3K27me3  H3K4me3 | ↑Proliferation  ↑Invasion | (Kim et al., 2014) |
| UTX | Unknown | ↑DICER | H3K27me3 | ↓EMT | (van den Beucken et al., 2014) |
| UTX | GATA3 | ↑DICER | H3K27me3 | ↓EMT | (Yu et al., 2019) |
| UTX | Unknown | ↑NANOG ↑SOX2  ↑KLF4 | H3K27me3 | ↑CSCs | (Lu et al., 2020) |
| UTX | MLL4  LSD1  HDAC1  DNMTs | ↓SNAIL  ↓ZEB1  ↓ZEB2 | H3K4me2  H3pan-ac | ↓EMT | (Choi et al., 2015) |
| UTX | JHDM1D  CBP | ↑CXCR4 | H3K27me3  H3K27ac | ↑Proliferation  ↑Migration | (Xie et al., 2017) |
| KDM6B | Unknown | ↑SNAI1 | H3K27me3 | ↑EMT  ↑Invasion | (Ramadoss et al., 2012) |
| KDM6B | Unknown | ↑BCL2 | H3K27me3 | ↓Apoptosis | (Svotelis et al., 2011) |
| KDM6B | Unknown | ↑IGFBP5 | H3K27me3 | ↓Proliferation | (Wang et al., 2018) |

Note: ↑, promote; ↓, inhibit

**Table S2. Functions of histone acetylation and methylation readers in BC**

| Proteins | Readers | Targets | Target proteins | Function | Refs. |
| --- | --- | --- | --- | --- | --- |
| BRD4 | Bromodomain | H4K5ac  H4K8ac | ↑WNT5A | ↑Invasion  ↑Tumorigenicity  ↑CSCs | (Shi et al., 2014; Zhang et al., 2020) |
| BRD4 | Bromodomain | H3K9ac | ↑LIFR | ↓Apoptosis  ↓HDACi sensitivity | (Zeng et al., 2016) |
| BRD4 | Bromodomain | H4K12ac | Unknown | ↑Estrogen-dependent gene transcription | (Nagarajan et al., 2015) |
| BRD4 | Bromodomain | Acetyl-lysine | ↑BRCA1  ↑RAD51 | ↑DNA damage repair | (Mio et al., 2019) |
| TRIM24 | Tandem Plant Homeodomain and bromodomain | H3K4me0  H3K23ac | Unknown | ↑Adhesion | (Tsai et al., 2010; Appikonda et al., 2018) |
| HP1 | Chromodomain | H3K9me3 H3K27me3 | ↓ZIM2 | ↑Proliferation | (Hsu et al., 2016) |
| PHF20L1 | Tudor domain | H3K27me2 | ↓HIC1  ↓KISS1  ↓BRCA1 | ↑Proliferation  ↑Metastasis  ↑Glycolysis | (Hou et al., 2020) |
| BAP18 | PHD finger | H3K4me3 | ↑MYC | ↑Proliferation  ↑Tumor growth  ↓Antiestrogen sensitivity | (Sun et al., 2020) |
| ASXL2 | PHD finger | H3K4me2 | ↑TFF1 | ↑Proliferation  ↑Tumor growth | (Park et al., 2016) |
| KDM5B | PHD finger | H3K4me0 | Unknown | ↓Migration | (Klein et al., 2014) |

Note: ↑, promote; ↓, inhibit

T**able S3. Clinical trials of epi-drugs in BC**

| Targets | Drug | Therapeutic Strategy | Conditions | Phases | Refs./NCT no. |
| --- | --- | --- | --- | --- | --- |
| HDAC | Vorinostat (SAHA) | Polytherapy  (Vorinostat,  Olaparib) | Relapsed/Refractory  and/or metastatic BC | I  (Recruiting) | NCT03742245 |
|  |  | Monotherapy | BC | I, II  (Active, not recruiting) | NCT00416130 |
|  |  | Polytherapy  (Vorinostat, Paclitaxel, Trastuzumab, Doxorubicin, Cyclophosphamide) | Locally advanced BC | I, II  (Completed) | NCT00574587 |
|  |  | Monotherapy | BC | II  (Completed) | NCT00262834 |
|  |  | Polytherapy  (Vorinostat,  Carboplatin,  Nab-Paclitaxel) | Newly diagnosed  operable BC | II  (Active, not recruiting) | NCT00616967  (Connolly et al., 2015; Connolly et al., 2018) |
|  |  | Polytherapy  (Vorinostat,  Paclitaxel,  Bevacizumab) | Metastatic BC | I, II  (Completed) | NCT00368875  (Ramaswamy et al., 2012) |
|  |  | Polytherapy  (Vorinostat,  Anastrozole,  Letrozole, Exemestane) | Stage Ⅳ BC | Completed | NCT01720602 |
|  |  | Polytherapy  (Vorinostat, Trastuzumab) | Metastatic or locally recurrent BC | I, II  (Completed) | NCT00258349 |
|  |  | Polytherapy  (Vorinostat,  Anastrozole,  Letrozole, Exemestane) | Stage Ⅳ BC | Completed | NCT01153672 |
|  |  | Polytherapy  (Vorinostat,  Radiation) | BC patients with brain metastasis | I  (Completed) | NCT00838929 |
|  |  | Monotherapy | Relapsed or refractory BC | II  (Completed) | (Vansteenkiste et al., 2008) |
|  |  | Polytherapy  (Vorinostat,  Paclitaxel,  Doxorubicin-Cyclophosphamide) | Locally advanced BC | I, II  (Completed) | (Tu et al., 2014) |
|  |  | Polytherapy  (Vorinostat,  Tamoxifen,  Pembrolizumab) | BC | II  (Terminated) | NCT02395627  (Terranova-Barberio et al., 2020) |
|  |  | Polytherapy  (Vorinostat,  Pembrolizumab,  Tamoxifen) | ER-positive BC | II  (Active, not recruiting) | NCT04190056 |
|  |  | Polytherapy  (Vorinostat,  Tamoxifen) | Hormone therapy-resistant BC | II  (Completed) | NCT00365599  (Munster et al., 2011) |
|  |  | Polytherapy  (Vorinostat,  Doxorubicin) | BC | I  (Completed) | NCT00331955  (Munster et al., 2009) |
|  |  | Polytherapy  (Vorinostat,  Ixabepilone) | Metastatic BC | I  (Completed) | NCT01084057 |
|  |  | Monotherapy | BC | I  (Completed) | NCT00788112 |
|  | Belinostat (PXD101) | Polytherapy  (Belinostat,  Ribociclib) | Metastatic BC | I  (Recruiting) | NCT04315233 |
|  |  | Polytherapy  (Belinostat,  Talazoparib) | Metastatic BC | I  (Recruiting) | NCT04703920 |
|  |  | Polytherapy  (Belinostat,  Trastuzumab) | BC | I  (Suspended) | NCT03432741 |
|  | Panobinostat (LBH- 589) | Polytherapy  (Panobinostat,  Letrozole) | Metastatic BC | I, II  (Completed) | NCT01105312  (Tan et al., 2016) |
|  |  | Monotherapy | HER2 -negative locally recurrent or metastatic BC | II  (Completed) | NCT00777049 |
|  |  | Polytherapy  (Panobinostat,  Paclitaxel,  Trastuzumab) | HER2-positive or metastatic BC | I  (Completed) | NCT00788931 |
|  |  | Polytherapy  (Panobinostat,  Capecitabine,  Lapatinib) | BC | I  (Completed) | NCT00632489 |
|  |  | Polytherapy  (Panobinostat,  Everolimus,  LCL161, QBM076,  HDM201) | TNBC | I  (Active, not recruiting) | NCT02890069 |
|  | Romidepsin | Monotherapy | BC | I  (Active, not  recruiting) | NCT01638533 |
|  |  | Polytherapy  (Romidepsin,  Cisplatin,  Nivolumab) | Metastatic TNBC, BRCA mutation-associated locally recurrent or metastatic BC | I, II  (Suspended) | NCT02393794 |
|  |  | Polytherapy  (Romidepsin,  Abraxane) | Metastatic inflammatory BC | I, II  (Terminated) | NCT01938833 |
|  |  | Monotherapy | Metastatic BC | II  (Completed) | NCT00098397 |
|  | Valproic acid (VPA) | Polytherapy  (Magnesium valproate,  Hydralazine, Doxorubicin, Cyclophosphamide) | BC | II  (Terminated) | NCT00395655 |
|  |  | Polytherapy  (Valproic acid  Epirubicin,  5-fluorouracil,  Cyclophosphamide) | BC | I  (Completed) | NCT00246103  (Munster et al., 2009) |
|  |  | Polytherapy  (Valproic acid Temsirolimus,  Cetuximab， Bevacizuma) | Recurrent BC | I  (Recruiting) | NCT01552434 |
|  | Ricolinostat  (ACY-1215) | Polytherapy  (ACY-1215,  Nab-paclitaxel) | Metastatic BC | I  (Completed) | NCT02632071 |
|  | Mocetinostat  (MGCD0103) | Polytherapy (MGCD0103,  Docetaxel) | BC | I  (Terminated) | NCT00511576 |
|  | CUDC-101 | Monotherapy | BC | I  (Completed) | NCT01171924  (Lai et al., 2010) |
|  | Entinostat (SNDX-275) | Polytherapy  (Entinostat, Exemestane) | Postmenopausal  woman with advanced BC | II  (Completed) | NCT00676663  (Yardley et al., 2013) |
|  |  | Polytherapy  (Entinostat,  Exemestane) | Postmenopausal  woman with ER-positive BC | I  (Active, not  recruiting) | NCT02820961 |
|  |  | Polytherapy  (Entinostat,  Atezolizuma,Placebo) | TNBC | I  (Active, not  recruiting) | NCT02708680 |
|  |  | Polytherapy  (Entinostat, BN-Brachyury, Adotrastuzumab,  M7824) | Advanced stage BC | I  (Recruiting) | NCT04296942 |
|  |  | Polytherapy  (Entinostat,  Exemestane,  Goserelin) | Recurrent hormone receptor-positive BC | III  (Active, not  recruiting) | NCT02115282 |
|  |  | Polytherapy  (Entinostat,  Nivolumab,  Lpilimumab) | Metastatic or locally advanced BC | I  (Active, not  recruiting) | NCT02453620 |
|  |  | Monotherapy | TNBC | I  (Terminated) | NCT03361800 |
|  |  | Polytherapy  (Entinostat,  Capecitabine) | Metastatic BC, high risk BC after neo-adjuvant therapy | I  (Recruiting) | NCT03473639 |
|  |  | Polytherapy  (Entinostat,  Exemestane) | Advanced or recurrent BC | I  (Active, not  recruiting) | NCT02623751 |
|  |  | Polytherapy  (Entinostat,  Exemestane,  Placebo) | Hormone receptor-positive, locally advanced or metastatic BC | III  (Active, not  recruiting) | NCT03538171 |
|  |  | Polytherapy  (Entinostat,  Exemestane,  Placebo) | Advanced or recurrent BC | II  (Active, not  Recruiting) | NCT03291886 |
|  |  | Polytherapy  (Entinostat,  Exemestane,  Atezolizumab,etc) | Hormone receptor-positive and HER2-negative BC | I, II  (Recruiting) | NCT03280563 |
|  |  | Polytherapy  (Entinostat,  Exemestane,  Erlotinib) | BC | I  (Completed) | NCT01594398  (Witta et al., 2012) |
|  |  | Polytherapy  (Entinostat,  Lapatinib,  Trastuzumab) | Locally recurrent or distant relapsed metastatic BC | I  (Completed) | NCT01434303 |
|  |  | Polytherapy  (Entinostat,  Azactidine) | Advanced BC | II  (Active, not recruiting) | NCT01349959 |
|  |  | Monotherapy | ER-positive BC | II  (Completed) | NCT00828854 |
| SIRT | Suramin | Polytherapy  (Suramin,  Paclitaxel) | BC | I, II  (Completed) | NCT00054028 |
| BET | GSK525762 | Polytherapy  (GSK525762,  Fulvestrant) | Hormone receptor-positive/HER2-negative advanced or metastatic BC | II  (Active, not recruiting) | NCT02964507 |
|  | ABBV-075 | Polytherapy (ABBV-075,  Venetoclax) | BC | I  (Completed) | NCT02391480  (Piha-Paul et al., 2019) |
|  | ZEN003694 | Polytherapy (ZEN003694,  Talazoparib) | TNBC | II  (Active, not recruiting) | NCT03901469 |
|  | GS-5829 | Polytherapy  (GS-5829,  Exemestane,  Fulvestrant) | ER-positive BC | I  (Completed) | NCT02392611 |
|  | MK-8628 | Monotherapy | TNBC | I  (Terminated) | NCT02698176 |
| EZH2 | SHR2554 | Polytherapy  (SHR2554,  SHR3162,  SHR3680) | Luminal advanced  BC | II  (Not yet recruiting) | NCT04355858 |
| G9a | Phenelzine | Polytherapy  (Phenelzine sulfate,  Abraxane) | Metastatic or advanced BC | I  (Completed) | NCT03505528 |
| PRMT5 | GSK3326595 | Monotherapy | BC | II  (Not net recruiting) | NCT04676516 |

Note: ↑, promote; ↓, inhibit

**Table S4. Epi-drugs and functions in BC**

| Agents | Targets | Functions | Structure | Refs. |
| --- | --- | --- | --- | --- |
| KATis | | | | |
| L002 | P300 | ↓Proliferation  ↓Tumor growth  ↑Apoptosis |  | (Yang et al., 2013) |
| Quercetin | P300 | ↓Angiogenesis |  | (Xiao et al., 2011) |
| TH1834 | TIP60  MOF | ↑Radiosensitivity  ↑Apoptosis |  | (Gao et al., 2014) |
| Garcinol | TIP60 | ↓Proliferation  ↑Apoptosis |  | (Balasubramanyam et al., 2004; Ye et al., 2014) |
| HDACis | | | | |
| Vorinostat (SAHA) | HDAC1,2,3,7,11 | ↓Proliferation ↓Invasion  ↓Migration  ↓EMT  ↓Tamoxifen resistance  ↑Cell cycle arrest  ↑Apoptosis ↑Autophagy ↑Differentiation  ↑Anoikis  ↑Drug sensitivity |  | (Shi et al., 2010; Bellarosa et al., 2012; Lauricella et al., 2012; Lee et al., 2012; Carlisi et al., 2015; Min et al., 2015; Tang et al., 2015; Lee et al., 2016; Wu et al., 2016; Zhou et al., 2016; Wawruszak et al., 2019; Steed et al., 2020) |
| Belinostat (PXD101) | HDAC6 | ↓Proliferation  ↓Invasion |  | (Zuo et al., 2020) |
| Panobinostat (LBH-589) | Pan-HDACi | ↓Proliferation  ↓Invasion  ↓Migration  ↓EMT |  | (Zhou et al., 2007; Rhodes et al., 2014) |
| Romidepsin (FK2280) | HDAC1,2,4,6 | ↓Proliferation ↑Apoptosis |  | (Cooper et al., 2012; Robertson et al., 2013; Pattarawat et al., 2020) |
| Valproic acid (VPA) | HDAC1 | ↑Cell cycle arrest  ↓Migration  ↑EMT  ↑Apoptosis  ↑Drug sensitivity  ↓Tamoxifen resistance |  | (Marchion et al., 2005; Hodges-Gallagher et al., 2007; Fortunati et al., 2008; Arakawa et al., 2009; Travaglini et al., 2009; Fortunati et al., 2010; Zhang et al., 2012; Reddy et al., 2015; Wawruszak et al., 2015; Terranova-Barberio et al., 2016; Tian et al., 2017; Zhang et al., 2019; Laengle et al., 2020) |
| Entinostat (MS-275) | HDAC1,2,3 | ↑Immunomodulatory  ↓Drug resistance  ↓ EMT |  | (Lee et al., 2014; Shah et al., 2014; Schech et al., 2015; Schech et al., 2015; Merino et al., 2016; Christmas et al., 2018; Li et al., 2018; Liu et al., 2018; Lim et al., 2019; McCaw et al., 2019) |
| LW479 | HDAC1 | ↓Proliferation ↓Metastasis |  | (Li et al., 2015) |
| MHY218 | HDAC1,4,6 | ↑Apoptosis  ↑Autophagy |  | (Park et al., 2012) |
| Liposomal trichostatin A | HDAC1 | ↑Apoptosis  ↓Cell cycle progression ↓Tumor growth |  | (Urbinati et al., 2011) |
| KBH-A145 | HDAC1 | ↓Proliferation ↑Apoptosis |  | (Kwon et al., 2009) |
| Troglitazone (TRG) | HDAC1 | ↓Viability |  | (Davies et al., 2010) |
| C02S | HDAC1  DNMT1  DNMT3A  DNMT3B | ↓Proliferation  ↓Invasion  ↓Migration  ↓Angiogenesis |  | (Yuan et al., 2019) |
| Crocetin (β-d-glucosyl) ester | HDAC2 | ↓Proliferation |  | (Mir et al., 2020) |
| Resveratrol (RVT) | HDAC2 | ↓Viability  ↑Apoptosis |  | (Izquierdo-Torres et al., 2019) |
| Oleuropein | HDAC2,3,4 | ↓Proliferation  ↓Invasion ↓Migration ↑Apoptosis |  | (Bayat et al., 2019; Mansouri et al., 2019) |
| TMU-35435 | HDAC6 | ↑Radiosensitivity  ↑Autophagy |  | (Chiu et al., 2019) |
| MPT0G211 | HDAC6 | ↓Migration  ↓Metastasis |  | (Hsieh et al., 2019) |
| 4-Hydroxybenzoic acid (4-HBA) | HDAC6 | ↑Adriamycin sensitivity |  | (Wang et al., 2018) |
| Ricolinostat (ACY1215) | HDAC6 | ↓Proliferation |  | (Putcha et al., 2015) |
| CG0006 | HDAC6 | ↓Proliferation |  | (Kim et al., 2011) |
| LYP-2 | HDAC6 | ↓Proliferation  ↑Bortezomib sensitivity |  | (Zhao et al., 2019) |
| LYP-6 |  |  |  |  |
| Chrysin | HDAC8 | ↓Proliferation  ↑Differentiation |  | (Sun et al., 2012) |
| MHY2256 | SIRT1 | ↑Apoptosis  ↑Autophagy  ↓Tumor growth |  | (Park et al., 2016) |
| Sulforaphane | SIRT1 | ↓Viability  ↓Stemness  ↓Metastasis |  | (Sinha et al., 2019; Sinha et al., 2021) |
| Amurensin G | SIRT1 | ↓Doxorubicin resistance |  | (Oh et al., 2010) |
| Psammaplin A | SIRT1 | ↓Proliferation  ↓Doxorubicin resistance |  | (Kim et al., 2015) |
| Oleuropein | SIRT1 | ↓Migration  ↓EMT  ↑Doxorubicin sensitivity |  | (Choupani et al., 2019) |
| ILS-JGB-1741(JGB1741) | SIRT1 | ↓Proliferation  ↑Apoptosis |  | (Kalle et al., 2010) |
| Splitomicin | SIRT2 | ↓Motility  ↑Paclitaxel sensitivity |  | (Bonezzi et al., 2012) |
| ICL-SIRT078 | SIRT2 | ↓Proliferation |  | (Di Fruscia et al., 2015) |
| Thioamide 53 | SIRT2 | ↓Proliferation |  | (Mellini et al., 2019) |
| RK-9123016 | SIRT2 | ↓Viability |  | (Shah et al., 2016) |
| γ-mangostin | SIRT2 | ↓Proliferation |  | (Yeong et al., 2020) |
| Cambinol | SIRT1  SIRT2 | ↓Invasion |  | (Holloway et al., 2013) |
| Sirtinol | SIRT1  SIRT2 | ↓Proliferation  ↑Apoptosis  ↑Autophagy  ↑Senescence |  | (Ota et al., 2006; Peck et al., 2010; Wang et al., 2012) |
| Salermide | SIRT1  SIRT2 | ↓Proliferation |  | (Peck, Chen et al., 2010) |
| Selisistat  (EX-527) | SIRT1  SIRT2 | ↑Cell cycle arrest |  | (Peck, Chen et al., 2010) |
| BETis | | | | |
| JQ1 | BRD2  BRD4 | ↓Proliferation  ↓Invasion ↓Migration ↓Mitotic  ↓CSCs  ↓Angiogenesis ↓Inflammatory response ↑Immunogenicity  ↑Drug sensitivity  ↓Drug resistance |  | (Belkina et al., 2013; Feng et al., 2014; Shi et al., 2014; Bihani et al., 2015; Borbely et al., 2015; Stratikopoulos et al., 2015; Andrieu et al., 2016; Marcotte et al., 2016; Pérez-Peña et al., 2016; Shu et al., 2016; da Motta et al., 2017; Sahni et al., 2017; Yang et al., 2017; Arfaoui et al., 2019; Maggisano et al., 2019; Tian et al., 2019; Walsh et al., 2019; Jing et al., 2020; Lai et al., 2020; Qiao et al., 2020; Qiao et al., 2020; Serrano-Oviedo et al., 2020) |
| I-BET151  (GSK2820151) | BRD2  BRD3  BRD4 | ↓Viability  ↑Apoptosis  ↑Ferroptosis |  | (Qiao, Chen et al., 2020; Qiao, Chen et al., 2020) |
| I-BET762  (GSK525762) | BRD2  BRD 3  BRD 4  BRDT | ↓Cell growth  ↓Lapatinib resistance |  | (Stuhlmiller et al., 2015) |
| Birabresib  (OTX015，  MK-8628) | BRD2  BRD 3  BRD 4 | ↓Proliferation  ↑Everolimus sensitivity |  | (Vázquez et al., 2017) |
| HMTis | | | | |
| GSK2816126 | EZH2 | ↓Proliferation  ↓Invasion  ↑Platinum sensitivity |  | (Gong et al., 2015; Gong et al., 2016; Hirukawa et al., 2018; Puppe et al., 2019) |
| GSK343 | EZH2 | ↓Proliferation  ↑Apoptosis  ↑Senescence  ↑Adriamycin sensitivity |  | (Yu et al., 2019; Zhang et al., 2020) |
| ZLD1039 | EZH2 | ↓Proliferation  ↓Metastasis  ↑Apoptosis |  | (Song et al., 2016) |
| MS1943 | EZH2 | ↓Proliferation  ↑EZH2 degradation |  | (Ma et al., 2020) |
| Curcumin | EZH2 | ↓Proliferation |  | (Hua et al., 2010) |
| Green tea polyphenols (GTP), epigallocatechin3-gallate (EGCG) | EZH2 | ↓Invasion |  | (Deb et al., 2015) |
| Resveratrol (RVT) | EZH2 | ↓Proliferation |  | (Chatterjee et al., 2019; Hu et al., 2019) |
| Protoberberine alkaloid pseudodehydrocorydaline (CT13) | G9a | ↓Proliferation |  | (Chen et al., 2018) |
| UNC0638 | G9a | ↓Migration  ↓Invasion  ↓EMT |  | (Liu et al., 2018) |
| GA001 | G9a | ↑Autophagy  ↑Apoptosis |  | (Zhang et al., 2017) |
| BIX-01249 | G9a | ↓Invasion  ↓Tumor growth  ↑Apoptosis  ↑Autophagy | 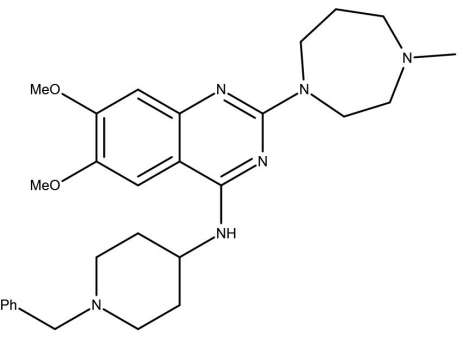 | (Kim et al., 2013; Kim et al., 2018; Kim et al., 2018) |
| UNC0646 | G9a | ↓Viability |  | (Liu et al., 2011) |
| UNC0631 |  |  |  |  |
| HKMT-1-005 | EZH2  G9a | ↓Viability | 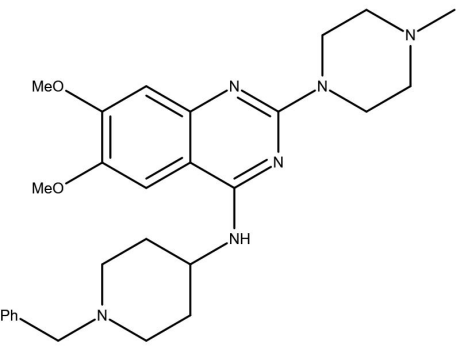 | (Curry et al., 2015) |
| HKMT-1-011 |  |  | 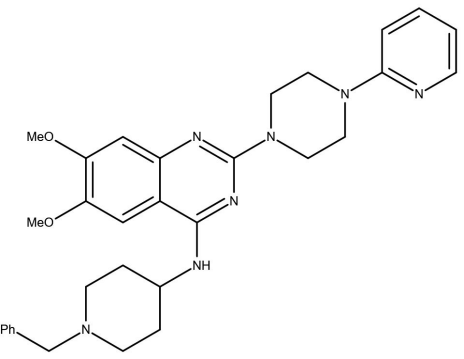 |  |
| HKMT-1-022 |  |  | 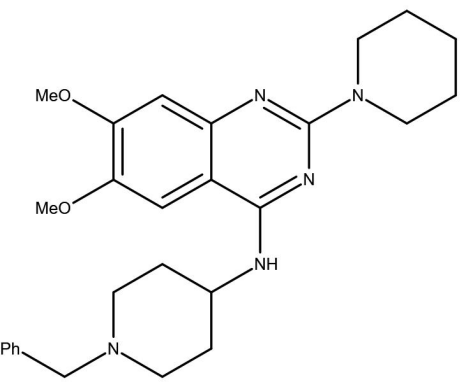 |  |
| LLY-507 | SMYD2 | ↓Proliferation |  | (Nguyen et al., 2015) |
| AZ505 | SMYD2 | ↓Proliferation  ↓Migration  ↑Apoptosis |  | (Li et al., 2018) |
| OTS186935 | SUV39H2 | ↓Tumor growth  ↑Doxorubicin sensitivity |  | (Vougiouklakis et al., 2018) |
| DC21 | SET7 | ↓Proliferation |  | (Hou et al., 2020) |
| DC-S285 | SET7 | ↓Proliferation |  | (Ding et al., 2018) |
| Cyproheptadine | SET7 | ↓Viability |  | (Takemoto et al., 2016) |
| EPZ4777 | DOT1L | ↓Proliferation  ↓Self-renewal  ↓Metastasis  ↑Differentiation |  | (Zhang et al., 2014) |
| PsA-3091 | DOT1L | ↓Proliferation  ↓Invasion  ↓Migration |  | (Byun et al., 2019) |
| Fluoro-neplanocin A (F-NepA) | DOT1L | ↓Proliferation  ↓Invasion  ↓Migration |  | (Byun et al., 2020) |
| MS023 | PRMT1  PRMT3  PRMT4  PRMT6  PRMT8 | ↓Proliferation | 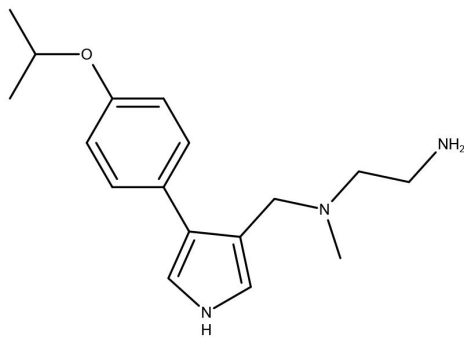 | (Eram et al., 2016) |
| DCLX069 | PRMT1 | ↓Proliferation |  | (Xie et al., 2014) |
| DCLX078 |  |  | 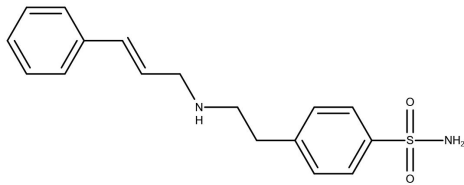 |  |
| Naproxen | PRMT1 | ↓Proliferation | 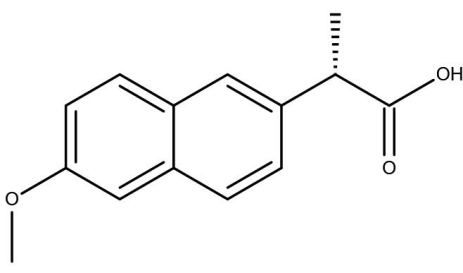 | (T et al., 2016) |
| Salvianolic acid A |  |  | 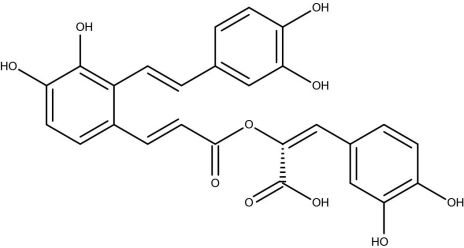 |  |
| SKI-73 | PRMT4 | ↓Invasion | 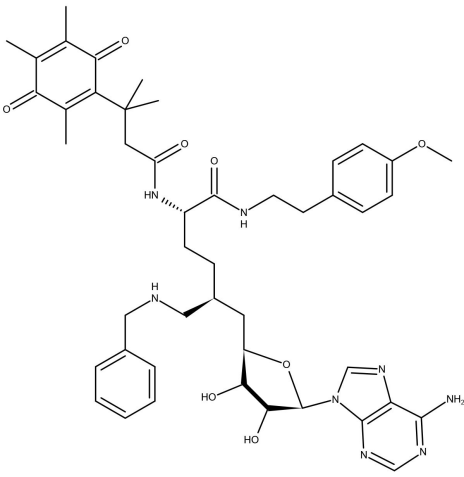 | (Cai et al., 2019) |
| LLY-283 | PRMT5 | ↓Proliferation |  | (Bonday et al., 2018) |
| Curcumin | PRMT5 | ↓Viability |  | (Chatterjee et al., 2019) |
| Licochalcone A | PRMT6 | ↓Proliferation  ↑Apoptosis | 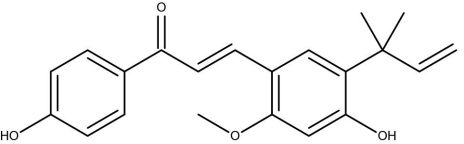 | (Gong et al., 2020) |
| KDMis | | | | |
| Tranylcypromine | LSD1 | ↓Migration  ↓Invasion  ↓Tumor growth  ↓Metastasis |  | (Ferrari-Amorotti et al., 2014) |
| Pargyline | LSD1 | ↓Proliferation  ↓Tumor growth  ↑Apoptosis |  | (Cortez et al., 2012; Vasilatos et al., 2013) |
| Phenelzine | LSD1 | ↑Immunogenicity |  | (Tan et al., 2019) |
| Iadademstat (ORY-1001) | LSD1 | ↓Mammosphere formation |  | (Cuyàs et al., 2020) |
| Isoquercitrin | LSD1 | ↑Apoptosis |  | (Xu et al., 2019) |
| 3-chloro-N0 -(2-hydroxybenzylidene) benzohydrazide (CHBH) | LSD1 | ↓Proliferation |  | (Sarno et al., 2018) |
| OBHS-LSD1i conjugate 11g | LSD1  ER | ↓Proliferation  ↑Apoptosis |  | (He et al., 2020) |
| MC3324 | LSD1  UTX | ↓Proliferation  ↓Tamoxifen resistance  ↑Apoptosis |  | (Benedetti et al., 2019) |
| YUKA1 | KDM5A | ↓Proliferation  ↓Trastuzumab resistance |  | (Gale et al., 2016) |
| Cyclopenta[c]chromen derivative 1 | KDM5A | ↓Proliferation | 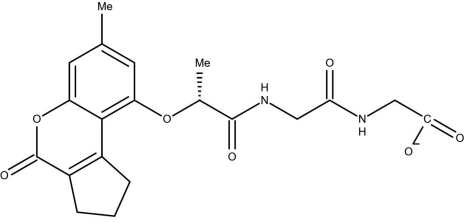 | (Yang et al., 2019) |
| Rhodiu(III) complex 1 | KDM5A | ↓Proliferation |  | (Yang et al., 2018) |
| KDM5-inh1 | KDM5A | ↓Proliferation  ↓Trastuzumab resistance  ↓Lapatinib resistance |  | (Paroni et al., 2019) |
| 2-4(4-methylphenyl)-1,2-  benzisothiazol-3(2H)-one (PBIT) | KDM5B | ↓Proliferation |  | (Sayegh et al., 2013) |
| CPI-455 | KDM5B | ↓Viability |  | (Leadem et al., 2018) |
| QC6352 | KDM4 | ↓CSCs  ↓Sphere formation |  | (Metzger et al., 2017) |
| GSKJ4 | KDM6A/B | ↓Proliferation |  | (Yan et al., 2017) |
| Deferiprone (DFP)，  Derivatives | Pan-KDMi | ↓ Proliferation  ↓Tumor growth |  | (Khodaverdian et al., 2019) |

Note: ↑, promote; ↓, inhibit

**References**

Bai, J. W., Chen, M. N., Wei, X. L., Li, Y. C., Lin, H. Y., Chen, M., et al. (2017). The zinc-finger transcriptional factor Slug transcriptionally downregulates ERα by recruiting lysine-specific demethylase 1 in human breast cancer. *Oncogenesis*. 6, e330. DOI: 10.1038/oncsis.2017.38

Bai, X. Y., Li, S., Wang, M., Li, X., Yang, Y., Xu, Z., et al. (2018). Krüppel-like factor 9 down-regulates matrix metalloproteinase 9 transcription and suppresses human breast cancer invasion. *Cancer Lett*. 412, 224-235. DOI: 10.1016/j.canlet.2017.10.027

Bhan, A., Hussain, I., Ansari, K. I., Kasiri, S., Bashyal, A. and Mandal, S. S. (2013). Antisense transcript long noncoding RNA (lncRNA) HOTAIR is transcriptionally induced by estradiol. *J Mol Biol*. 425, 3707-3722. DOI: 10.1016/j.jmb.2013.01.022

Cao, Q., Yu, J., Dhanasekaran, S. M., Kim, J. H., Mani, R. S., Tomlins, S. A., et al. (2008). Repression of E-cadherin by the polycomb group protein EZH2 in cancer. *Oncogene*. 27, 7274-7284. DOI: 10.1038/onc.2008.333

Casciello, F., Al-Ejeh, F., Kelly, G., Brennan, D. J., Ngiow, S. F., Young, A., et al. (2017). G9a drives hypoxia-mediated gene repression for breast cancer cell survival and tumorigenesis. *Proc Natl Acad Sci U S A*. 114, 7077-7082. DOI: 10.1073/pnas.1618706114

Casciello, F., Al-Ejeh, F., Miranda, M., Kelly, G., Baxter, E., Windloch, K., et al. (2020). G9a-mediated repression of CDH10 in hypoxia enhances breast tumour cell motility and associates with poor survival outcome. *Theranostics*. 10, 4515-4529. DOI: 10.7150/thno.41453

Cassandri, M., Butera, A., Amelio, I., Lena, A. M., Montanaro, M., Mauriello, A., et al. (2020). ZNF750 represses breast cancer invasion via epigenetic control of prometastatic genes. *Oncogene*. 39, 4331-4343. DOI: 10.1038/s41388-020-1277-5

Chang, C. C., Wu, M. J., Yang, J. Y., Camarillo, I. G. and Chang, C. J. (2015). Leptin-STAT3-G9a Signaling Promotes Obesity-Mediated Breast Cancer Progression. *Cancer Res*. 75, 2375-2386. DOI: 10.1158/0008-5472.Can-14-3076

Chang, C. J., Yang, J. Y., Xia, W., Chen, C. T., Xie, X., Chao, C. H., et al. (2011). EZH2 promotes expansion of breast tumor initiating cells through activation of RAF1-β-catenin signaling. *Cancer Cell*. 19, 86-100. DOI: 10.1016/j.ccr.2010.10.035

Chen, J. Y., Luo, C. W., Lai, Y. S., Wu, C. C. and Hung, W. C. (2017). Lysine demethylase KDM2A inhibits TET2 to promote DNA methylation and silencing of tumor suppressor genes in breast cancer. *Oncogenesis*. 6, e369. DOI: 10.1038/oncsis.2017.71

Chi, Y., Xue, J., Huang, S., Xiu, B., Su, Y., Wang, W., et al. (2019). CapG promotes resistance to paclitaxel in breast cancer through transactivation of PIK3R1/P50. *Theranostics*. 9, 6840-6855. DOI: 10.7150/thno.36338

Chiang, K., Zielinska, A. E., Shaaban, A. M., Sanchez-Bailon, M. P., Jarrold, J., Clarke, T. L., et al. (2017). PRMT5 Is a Critical Regulator of Breast Cancer Stem Cell Function via Histone Methylation and FOXP1 Expression. *Cell Rep*. 21, 3498-3513. DOI: 10.1016/j.celrep.2017.11.096

Chien, Y. C., Liu, L. C., Ye, H. Y., Wu, J. Y. and Yu, Y. L. (2018). EZH2 promotes migration and invasion of triple-negative breast cancer cells via regulating TIMP2-MMP-2/-9 pathway. *Am J Cancer Res*. 8, 422-434.

Cho, M. H., Park, J. H., Choi, H. J., Park, M. K., Won, H. Y., Park, Y. J., et al. (2015). DOT1L cooperates with the c-Myc-p300 complex to epigenetically derepress CDH1 transcription factors in breast cancer progression. *Nat Commun*. 6, 7821. DOI: 10.1038/ncomms8821

Choi, H. J., Park, J. H., Park, M., Won, H. Y., Joo, H. S., Lee, C. H., et al. (2015). UTX inhibits EMT-induced breast CSC properties by epigenetic repression of EMT genes in cooperation with LSD1 and HDAC1. *EMBO Rep*. 16, 1288-1298. DOI: 10.15252/embr.201540244

Chu, W., Zhang, X., Qi, L., Fu, Y., Wang, P., Zhao, W., et al. (2020). The EZH2-PHACTR2-AS1-Ribosome Axis induces Genomic Instability and Promotes Growth and Metastasis in Breast Cancer. *Cancer Res*. 80, 2737-2750. DOI: 10.1158/0008-5472.Can-19-3326

Crawford, N. T., McIntyre, A. J., McCormick, A., D'Costa, Z. C., Buckley, N. E. and Mullan, P. B. (2019). TBX2 interacts with heterochromatin protein 1 to recruit a novel repression complex to EGR1-targeted promoters to drive the proliferation of breast cancer cells. *Oncogene*. 38, 5971-5986. DOI: 10.1038/s41388-019-0853-z

Cutano, V., Di Giorgio, E., Minisini, M., Picco, R., Dalla, E. and Brancolini, C. (2019). HDAC7-mediated control of tumour microenvironment maintains proliferative and stemness competence of human mammary epithelial cells. *Mol Oncol*. 13, 1651-1668. DOI: 10.1002/1878-0261.12503

Deb, P., Bhan, A., Hussain, I., Ansari, K. I., Bobzean, S. A., Pandita, T. K., et al. (2016). Endocrine disrupting chemical, bisphenol-A, induces breast cancer associated gene HOXB9 expression in vitro and in vivo. *Gene*. 590, 234-243. DOI: 10.1016/j.gene.2016.05.009

Dong, C., Wu, Y., Wang, Y., Wang, C., Kang, T., Rychahou, P. G., et al. (2013). Interaction with Suv39H1 is critical for Snail-mediated E-cadherin repression in breast cancer. *Oncogene*. 32, 1351-1362. DOI: 10.1038/onc.2012.169

Dong, C., Wu, Y., Yao, J., Wang, Y., Yu, Y., Rychahou, P. G., et al. (2012). G9a interacts with Snail and is critical for Snail-mediated E-cadherin repression in human breast cancer. *J Clin Invest*. 122, 1469-1486. DOI: 10.1172/jci57349

Dong, C., Yuan, T., Wu, Y., Wang, Y., Fan, T. W., Miriyala, S., et al. (2013). Loss of FBP1 by Snail-mediated repression provides metabolic advantages in basal-like breast cancer. *Cancer Cell*. 23, 316-331. DOI: 10.1016/j.ccr.2013.01.022

Dong, H., Hu, J., Zou, K., Ye, M., Chen, Y., Wu, C., et al. (2019). Activation of LncRNA TINCR by H3K27 acetylation promotes Trastuzumab resistance and epithelial-mesenchymal transition by targeting MicroRNA-125b in breast Cancer. *Mol Cancer*. 18, 3. DOI: 10.1186/s12943-018-0931-9

Dong, H., Wang, W., Mo, S., Chen, R., Zou, K., Han, J., et al. (2018). SP1-induced lncRNA AGAP2-AS1 expression promotes chemoresistance of breast cancer by epigenetic regulation of MyD88. *J Exp Clin Cancer Res*. 37, 202. DOI: 10.1186/s13046-018-0875-3

Dong, H. T., Liu, Q., Zhao, T., Yao, F., Xu, Y., Chen, B., et al. (2020). Long Non-coding RNA LOXL1-AS1 Drives Breast Cancer Invasion and Metastasis by Antagonizing miR-708-5p Expression and Activity. *Mol Ther Nucleic Acids*. 19, 696-705. DOI: 10.1016/j.omtn.2019.12.016

Dou, D., Ge, X., Wang, X., Xu, X., Zhang, Z., Seng, J., et al. (2019). EZH2 Contributes To Cisplatin Resistance In Breast Cancer By Epigenetically Suppressing miR-381 Expression. *Onco Targets Ther*. 12, 9627-9637. DOI: 10.2147/ott.S214104

Du, D., Katsuno, Y., Meyer, D., Budi, E. H., Chen, S. H., Koeppen, H., et al. (2018). Smad3-mediated recruitment of the methyltransferase SETDB1/ESET controls Snail1 expression and epithelial-mesenchymal transition. *EMBO Rep*. 19, 135-155. DOI: 10.15252/embr.201744250

Du, J., Li, L., Ou, Z., Kong, C., Zhang, Y., Dong, Z., et al. (2012). FOXC1, a target of polycomb, inhibits metastasis of breast cancer cells. *Breast Cancer Res Treat*. 131, 65-73. DOI: 10.1007/s10549-011-1396-3

El Messaoudi, S., Fabbrizio, E., Rodriguez, C., Chuchana, P., Fauquier, L., Cheng, D., et al. (2006). Coactivator-associated arginine methyltransferase 1 (CARM1) is a positive regulator of the Cyclin E1 gene. *Proc Natl Acad Sci U S A*. 103, 13351-13356. DOI: 10.1073/pnas.0605692103

Feng, J., Li, L., Zhang, N., Liu, J., Zhang, L., Gao, H., et al. (2017). Androgen and AR contribute to breast cancer development and metastasis: an insight of mechanisms. *Oncogene*. 36, 2775-2790. DOI: 10.1038/onc.2016.432

Fenizia, C., Bottino, C., Corbetta, S., Fittipaldi, R., Floris, P., Gaudenzi, G., et al. (2019). SMYD3 promotes the epithelial-mesenchymal transition in breast cancer. *Nucleic Acids Res*. 47, 1278-1293. DOI: 10.1093/nar/gky1221

Frietze, S., Lupien, M., Silver, P. A. and Brown, M. (2008). CARM1 regulates estrogen-stimulated breast cancer growth through up-regulation of E2F1. *Cancer Res*. 68, 301-306. DOI: 10.1158/0008-5472.Can-07-1983

Fujii, S., Ito, K., Ito, Y. and Ochiai, A. (2008). Enhancer of zeste homologue 2 (EZH2) down-regulates RUNX3 by increasing histone H3 methylation. *J Biol Chem*. 283, 17324-17332. DOI: 10.1074/jbc.M800224200

Gala, K., Li, Q., Sinha, A., Razavi, P., Dorso, M., Sanchez-Vega, F., et al. (2018). KMT2C mediates the estrogen dependence of breast cancer through regulation of ERα enhancer function. *Oncogene*. 37, 4692-4710. DOI: 10.1038/s41388-018-0273-5

Gao, Y., Zhao, Y., Zhang, J., Lu, Y., Liu, X., Geng, P., et al. (2016). The dual function of PRMT1 in modulating epithelial-mesenchymal transition and cellular senescence in breast cancer cells through regulation of ZEB1. *Sci Rep*. 6, 19874. DOI: 10.1038/srep19874

Gong, C., Qu, S., Lv, X. B., Liu, B., Tan, W., Nie, Y., et al. (2014). BRMS1L suppresses breast cancer metastasis by inducing epigenetic silence of FZD10. *Nat Commun*. 5, 5406. DOI: 10.1038/ncomms6406

Gong, C., Yao, S., Gomes, A. R., Man, E. P., Lee, H. J., Gong, G., et al. (2016). BRCA1 positively regulates FOXO3 expression by restricting FOXO3 gene methylation and epigenetic silencing through targeting EZH2 in breast cancer. *Oncogenesis*. 5, e214. DOI: 10.1038/oncsis.2016.23

He, H., Wang, D., Yao, H., Wei, Z., Lai, Y., Hu, J., et al. (2015). Transcriptional factors p300 and MRTF-A synergistically enhance the expression of migration-related genes in MCF-7 breast cancer cells. *Biochem Biophys Res Commun*. 467, 813-820. DOI: 10.1016/j.bbrc.2015.10.060

Hirukawa, A., Smith, H. W., Zuo, D., Dufour, C. R., Savage, P., Bertos, N., et al. (2018). Targeting EZH2 reactivates a breast cancer subtype-specific anti-metastatic transcriptional program. *Nat Commun*. 9, 2547. DOI: 10.1038/s41467-018-04864-8

Hou, J., Wu, J., Dombkowski, A., Zhang, K., Holowatyj, A., Boerner, J. L., et al. (2012). Genomic amplification and a role in drug-resistance for the KDM5A histone demethylase in breast cancer. *Am J Transl Res*. 4, 247-256.

Hou, M. F., Luo, C. W., Chang, T. M., Hung, W. C., Chen, T. Y., Tsai, Y. L., et al. (2017). The NuRD complex-mediated p21 suppression facilitates chemoresistance in BRCA-proficient breast cancer. *Exp Cell Res*. 359, 458-465. DOI: 10.1016/j.yexcr.2017.08.029

Hu, X., Xiang, D., Xie, Y., Tao, L., Zhang, Y., Jin, Y., et al. (2019). LSD1 suppresses invasion, migration and metastasis of luminal breast cancer cells via activation of GATA3 and repression of TRIM37 expression. *Oncogene*. 38, 7017-7034. DOI: 10.1038/s41388-019-0923-2

Huang, S., Chi, Y., Qin, Y., Wang, Z., Xiu, B., Su, Y., et al. (2018). CAPG enhances breast cancer metastasis by competing with PRMT5 to modulate STC-1 transcription. *Theranostics*. 8, 2549-2564. DOI: 10.7150/thno.22523

Jin, W., Chen, B. B., Li, J. Y., Zhu, H., Huang, M., Gu, S. M., et al. (2012). TIEG1 inhibits breast cancer invasion and metastasis by inhibition of epidermal growth factor receptor (EGFR) transcription and the EGFR signaling pathway. *Mol Cell Biol*. 32, 50-63. DOI: 10.1128/mcb.06152-11

Jin, W., Liu, Y., Chen, L., Zhu, H., Di, G. H., Ling, H., et al. (2011). Involvement of MyoD and c-myb in regulation of basal and estrogen-induced transcription activity of the BRCA1 gene. *Breast Cancer Res Treat*. 125, 699-713. DOI: 10.1007/s10549-010-0876-1

Jin, W., Liu, Y., Xu, S. G., Yin, W. J., Li, J. J., Yang, J. M., et al. (2010). UHRF1 inhibits MDR1 gene transcription and sensitizes breast cancer cells to anticancer drugs. *Breast Cancer Res Treat*. 124, 39-48. DOI: 10.1007/s10549-009-0683-8

Jozwik, K. M., Chernukhin, I., Serandour, A. A., Nagarajan, S. and Carroll, J. S. (2016). FOXA1 Directs H3K4 Monomethylation at Enhancers via Recruitment of the Methyltransferase MLL3. *Cell Rep*. 17, 2715-2723. DOI: 10.1016/j.celrep.2016.11.028

Kang, L. J., Yu, Z. H., Cai, J., He, R., Lu, J. T., Hou, C., et al. (2019). Reciprocal transrepression between FOXF2 and FOXQ1 controls basal-like breast cancer aggressiveness. *Faseb j*. 33, 6564-6573. DOI: 10.1096/fj.201801916R

Karadedou, C. T., Gomes, A. R., Chen, J., Petkovic, M., Ho, K. K., Zwolinska, A. K., et al. (2012). FOXO3a represses VEGF expression through FOXM1-dependent and -independent mechanisms in breast cancer. *Oncogene*. 31, 1845-1858. DOI: 10.1038/onc.2011.368

Karthik, I. P., Desai, P., Sukumar, S., Dimitrijevic, A., Rajalingam, K. and Mahalingam, S. (2018). E4BP4/NFIL3 modulates the epigenetically repressed RAS effector RASSF8 function through histone methyltransferases. *J Biol Chem*. 293, 5624-5635. DOI: 10.1074/jbc.RA117.000623

Kawazu, M., Saso, K., Tong, K. I., McQuire, T., Goto, K., Son, D. O., et al. (2011). Histone demethylase JMJD2B functions as a co-factor of estrogen receptor in breast cancer proliferation and mammary gland development. *PLoS One*. 6, e17830. DOI: 10.1371/journal.pone.0017830

Kim, H. Y., Park, J. H., Won, H. Y., Lee, J. Y. and Kong, G. (2015). CBX7 inhibits breast tumorigenicity through DKK-1-mediated suppression of the Wnt/β-catenin pathway. *Faseb j*. 29, 300-313. DOI: 10.1096/fj.14-253997

Kim, J., Shin, Y., Lee, S., Kim, M., Punj, V., Lu, J. F., et al. (2018). Regulation of Breast Cancer-Induced Osteoclastogenesis by MacroH2A1.2 Involving EZH2-Mediated H3K27me3. *Cell Rep*. 24, 224-237. DOI: 10.1016/j.celrep.2018.06.020

Kim, J. H., Sharma, A., Dhar, S. S., Lee, S. H., Gu, B., Chan, C. H., et al. (2014). UTX and MLL4 coordinately regulate transcriptional programs for cell proliferation and invasiveness in breast cancer cells. *Cancer Res*. 74, 1705-1717. DOI: 10.1158/0008-5472.Can-13-1896

Kim, K., Lee, J. M., Yu, Y. S., Kim, H., Nam, H. J., Moon, H. G., et al. (2017). RORα2 requires LSD1 to enhance tumor progression in breast cancer. *Sci Rep*. 7, 11994. DOI: 10.1038/s41598-017-12344-0

Kim, S. S., Lee, M. H. and Lee, M. O. (2020). Histone methyltransferases regulate the transcriptional expression of ERα and the proliferation of tamoxifen-resistant breast cancer cells. *Breast Cancer Res Treat*. 180, 45-54. DOI: 10.1007/s10549-019-05517-0

Kim, Y., Kim, Y. S., Kim, D. E., Lee, J. S., Song, J. H., Kim, H. G., et al. (2013). BIX-01294 induces autophagy-associated cell death via EHMT2/G9a dysfunction and intracellular reactive oxygen species production. *Autophagy*. 9, 2126-2139. DOI: 10.4161/auto.26308

Li, H., Liu, Q., Wang, Z., Fang, R., Shen, Y., Cai, X., et al. (2015). The oncoprotein HBXIP modulates the feedback loop of MDM2/p53 to enhance the growth of breast cancer. *J Biol Chem*. 290, 22649-22661. DOI: 10.1074/jbc.M115.658468

Li, L., Liang, Y., Kang, L., Liu, Y., Gao, S., Chen, S., et al. (2018). Transcriptional Regulation of the Warburg Effect in Cancer by SIX1. *Cancer Cell*. 33, 368-385.e367. DOI: 10.1016/j.ccell.2018.01.010

Li, L., Liu, X., He, L., Yang, J., Pei, F., Li, W., et al. (2017). ZNF516 suppresses EGFR by targeting the CtBP/LSD1/CoREST complex to chromatin. *Nat Commun*. 8, 691. DOI: 10.1038/s41467-017-00702-5

Li, Z., Dong, M., Fan, D., Hou, P., Li, H., Liu, L., et al. (2017). LncRNA ANCR down-regulation promotes TGF-β-induced EMT and metastasis in breast cancer. *Oncotarget*. 8, 67329-67343. DOI: 10.18632/oncotarget.18622

Li, Z., Yu, D., Li, H., Lv, Y. and Li, S. (2019). Long non‑coding RNA UCA1 confers tamoxifen resistance in breast cancer endocrinotherapy through regulation of the EZH2/p21 axis and the PI3K/AKT signaling pathway. *Int J Oncol*. 54, 1033-1042. DOI: 10.3892/ijo.2019.4679

Liang, Y., Hu, J., Li, J., Liu, Y., Yu, J., Zhuang, X., et al. (2015). Epigenetic Activation of TWIST1 by MTDH Promotes Cancer Stem-like Cell Traits in Breast Cancer. *Cancer Res*. 75, 3672-3680. DOI: 10.1158/0008-5472.Can-15-0930

Lin, C., Liao, W., Jian, Y., Peng, Y., Zhang, X., Ye, L., et al. (2017). CGI-99 promotes breast cancer metastasis via autocrine interleukin-6 signaling. *Oncogene*. 36, 3695-3705. DOI: 10.1038/onc.2016.525

Liu, J., Feng, J., Li, L., Lin, L., Ji, J., Lin, C., et al. (2020). Arginine methylation-dependent LSD1 stability promotes invasion and metastasis of breast cancer. *EMBO Rep*. 21, e48597. DOI: 10.15252/embr.201948597

Liu, X., Li, C., Zhang, R., Xiao, W., Niu, X., Ye, X., et al. (2018). The EZH2- H3K27me3-DNMT1 complex orchestrates epigenetic silencing of the wwc1 gene, a Hippo/YAP pathway upstream effector, in breast cancer epithelial cells. *Cell Signal*. 51, 243-256. DOI: 10.1016/j.cellsig.2018.08.011

Lu, H., Xie, Y., Tran, L., Lan, J., Yang, Y., Murugan, N. L., et al. (2020). Chemotherapy-induced S100A10 recruits KDM6A to facilitate OCT4-mediated breast cancer stemness. *J Clin Invest*. 130, 4607-4623. DOI: 10.1172/jci138577

Luan, Q. X., Zhang, B. G., Li, X. J. and Guo, M. Y. (2016). MiR-129-5p is downregulated in breast cancer cells partly due to promoter H3K27m3 modification and regulates epithelial-mesenchymal transition and multi-drug resistance. *Eur Rev Med Pharmacol Sci*. 20, 4257-4265.

Luo, W., Chang, R., Zhong, J., Pandey, A. and Semenza, G. L. (2012). Histone demethylase JMJD2C is a coactivator for hypoxia-inducible factor 1 that is required for breast cancer progression. *Proc Natl Acad Sci U S A*. 109, E3367-3376. DOI: 10.1073/pnas.1217394109

Luo, X. G., Zhang, C. L., Zhao, W. W., Liu, Z. P., Liu, L., Mu, A., et al. (2014). Histone methyltransferase SMYD3 promotes MRTF-A-mediated transactivation of MYL9 and migration of MCF-7 breast cancer cells. *Cancer Lett*. 344, 129-137. DOI: 10.1016/j.canlet.2013.10.026

Mahajan, K., Lawrence, H. R., Lawrence, N. J. and Mahajan, N. P. (2014). ACK1 tyrosine kinase interacts with histone demethylase KDM3A to regulate the mammary tumor oncogene HOXA1. *J Biol Chem*. 289, 28179-28191. DOI: 10.1074/jbc.M114.584425

Matkar, S., Sharma, P., Gao, S., Gurung, B., Katona, B. W., Liao, J., et al. (2015). An Epigenetic Pathway Regulates Sensitivity of Breast Cancer Cells to HER2 Inhibition via FOXO/c-Myc Axis. *Cancer Cell*. 28, 472-485. DOI: 10.1016/j.ccell.2015.09.005

Mitra, D., Das, P. M., Huynh, F. C. and Jones, F. E. (2011). Jumonji/ARID1 B (JARID1B) protein promotes breast tumor cell cycle progression through epigenetic repression of microRNA let-7e. *J Biol Chem*. 286, 40531-40535. DOI: 10.1074/jbc.M111.304865

Montano, M. M., Yeh, I. J., Chen, Y., Hernandez, C., Kiselar, J. G., de la Fuente, M., et al. (2019). Inhibition of the histone demethylase, KDM5B, directly induces re-expression of tumor suppressor protein HEXIM1 in cancer cells. *Breast Cancer Res*. 21, 138. DOI: 10.1186/s13058-019-1228-7

Mu, X., Chen, M., Xiao, B., Yang, B., Singh, S. and Zhang, B. (2019). EZH2 Confers Sensitivity of Breast Cancer Cells to Taxol by Attenuating p21 Expression Epigenetically. *DNA Cell Biol*. 38, 651-659. DOI: 10.1089/dna.2019.4699

Nie, L., Wei, Y., Zhang, F., Hsu, Y. H., Chan, L. C., Xia, W., et al. (2019). CDK2-mediated site-specific phosphorylation of EZH2 drives and maintains triple-negative breast cancer. *Nat Commun*. 10, 5114. DOI: 10.1038/s41467-019-13105-5

Oktyabri, D., Ishimura, A., Tange, S., Terashima, M. and Suzuki, T. (2016). DOT1L histone methyltransferase regulates the expression of BCAT1 and is involved in sphere formation and cell migration of breast cancer cell lines. *Biochimie*. 123, 20-31. DOI: 10.1016/j.biochi.2016.01.005

Park, S. E., Yi, H. J., Suh, N., Park, Y. Y., Koh, J. Y., Jeong, S. Y., et al. (2016). Inhibition of EHMT2/G9a epigenetically increases the transcription of Beclin-1 via an increase in ROS and activation of NF-κB. *Oncotarget*. 7, 39796-39808. DOI: 10.18632/oncotarget.9290

Park, U. H., Kang, M. R., Kim, E. J., Kwon, Y. S., Hur, W., Yoon, S. K., et al. (2016). ASXL2 promotes proliferation of breast cancer cells by linking ERα to histone methylation. *Oncogene*. 35, 3742-3752. DOI: 10.1038/onc.2015.443

Pedanou, V. E., Gobeil, S., Tabariès, S., Simone, T. M., Zhu, L. J., Siegel, P. M., et al. (2016). The histone H3K9 demethylase KDM3A promotes anoikis by transcriptionally activating pro-apoptotic genes BNIP3 and BNIP3L. *Elife*. 5 DOI: 10.7554/eLife.16844

Phalke, S., Mzoughi, S., Bezzi, M., Jennifer, N., Mok, W. C., Low, D. H., et al. (2012). p53-Independent regulation of p21Waf1/Cip1 expression and senescence by PRMT6. *Nucleic Acids Res*. 40, 9534-9542. DOI: 10.1093/nar/gks858

Pruitt, K., Zinn, R. L., Ohm, J. E., McGarvey, K. M., Kang, S. H., Watkins, D. N., et al. (2006). Inhibition of SIRT1 reactivates silenced cancer genes without loss of promoter DNA hypermethylation. *PLoS Genet*. 2, e40. DOI: 10.1371/journal.pgen.0020040

Qin, L., Xu, Y., Yu, X., Toneff, M. J., Li, D., Liao, L., et al. (2017). The histone demethylase Kdm3a is required for normal epithelial proliferation, ductal elongation and tumor growth in the mouse mammary gland. *Oncotarget*. 8, 84761-84775. DOI: 10.18632/oncotarget.21380

Qin, Y., Vasilatos, S. N., Chen, L., Wu, H., Cao, Z., Fu, Y., et al. (2019). Inhibition of histone lysine-specific demethylase 1 elicits breast tumor immunity and enhances antitumor efficacy of immune checkpoint blockade. *Oncogene*. 38, 390-405. DOI: 10.1038/s41388-018-0451-5

Qiu, R., Shi, H., Wang, S., Leng, S., Liu, R., Zheng, Y., et al. (2018). BRMS1 coordinates with LSD1 and suppresses breast cancer cell metastasis. *Am J Cancer Res*. 8, 2030-2045.

Ramadoss, S., Chen, X. and Wang, C. Y. (2012). Histone demethylase KDM6B promotes epithelial-mesenchymal transition. *J Biol Chem*. 287, 44508-44517. DOI: 10.1074/jbc.M112.424903

Ramadoss, S., Guo, G. and Wang, C. Y. (2017). Lysine demethylase KDM3A regulates breast cancer cell invasion and apoptosis by targeting histone and the non-histone protein p53. *Oncogene*. 36, 47-59. DOI: 10.1038/onc.2016.174

Rampalli, S., Pavithra, L., Bhatt, A., Kundu, T. K. and Chattopadhyay, S. (2005). Tumor suppressor SMAR1 mediates cyclin D1 repression by recruitment of the SIN3/histone deacetylase 1 complex. *Mol Cell Biol*. 25, 8415-8429. DOI: 10.1128/mcb.25.19.8415-8429.2005

Ray, A., Alalem, M. and Ray, B. K. (2013). Loss of epigenetic Kruppel-like factor 4 histone deacetylase (KLF-4-HDAC)-mediated transcriptional suppression is crucial in increasing vascular endothelial growth factor (VEGF) expression in breast cancer. *J Biol Chem*. 288, 27232-27242. DOI: 10.1074/jbc.M113.481184

Ren, G., Baritaki, S., Marathe, H., Feng, J., Park, S., Beach, S., et al. (2012). Polycomb protein EZH2 regulates tumor invasion via the transcriptional repression of the metastasis suppressor RKIP in breast and prostate cancer. *Cancer Res*. 72, 3091-3104. DOI: 10.1158/0008-5472.Can-11-3546

Roy, S. S., Gonugunta, V. K., Bandyopadhyay, A., Rao, M. K., Goodall, G. J., Sun, L. Z., et al. (2014). Significance of PELP1/HDAC2/miR-200 regulatory network in EMT and metastasis of breast cancer. *Oncogene*. 33, 3707-3716. DOI: 10.1038/onc.2013.332

Salz, T., Deng, C., Pampo, C., Siemann, D., Qiu, Y., Brown, K., et al. (2015). Histone Methyltransferase hSETD1A Is a Novel Regulator of Metastasis in Breast Cancer. *Mol Cancer Res*. 13, 461-469. DOI: 10.1158/1541-7786.Mcr-14-0389

Shao, P., Liu, Q., Maina, P. K., Cui, J., Bair, T. B., Li, T., et al. (2017). Histone demethylase PHF8 promotes epithelial to mesenchymal transition and breast tumorigenesis. *Nucleic Acids Res*. 45, 1687-1702. DOI: 10.1093/nar/gkw1093

Shinchi, Y., Hieda, M., Nishioka, Y., Matsumoto, A., Yokoyama, Y., Kimura, H., et al. (2015). SUV420H2 suppresses breast cancer cell invasion through down regulation of the SH2 domain-containing focal adhesion protein tensin-3. *Exp Cell Res*. 334, 90-99. DOI: 10.1016/j.yexcr.2015.03.010

Si, X., Zang, R., Zhang, E., Liu, Y., Shi, X., Zhang, E., et al. (2016). LncRNA H19 confers chemoresistance in ERα-positive breast cancer through epigenetic silencing of the pro-apoptotic gene BIK. *Oncotarget*. 7, 81452-81462. DOI: 10.18632/oncotarget.13263

Siouda, M., Dujardin, A. D., Barbollat-Boutrand, L., Mendoza-Parra, M. A., Gibert, B., Ouzounova, M., et al. (2020). CDYL2 Epigenetically Regulates MIR124 to Control NF-κB/STAT3-Dependent Breast Cancer Cell Plasticity. *iScience*. 23, 101141. DOI: 10.1016/j.isci.2020.101141

Song, Z., Zhang, X., Lin, Y., Wei, Y., Liang, S. and Dong, C. (2019). LINC01133 inhibits breast cancer invasion and metastasis by negatively regulating SOX4 expression through EZH2. *J Cell Mol Med*. 23, 7554-7565. DOI: 10.1111/jcmm.14625

Soung, Y. H., Pruitt, K. and Chung, J. (2014). Epigenetic silencing of ARRDC3 expression in basal-like breast cancer cells. *Sci Rep*. 4, 3846. DOI: 10.1038/srep03846

Su, C. H., Lin, I. H., Tzeng, T. Y., Hsieh, W. T. and Hsu, M. T. (2016). Regulation of IL-20 Expression by Estradiol through KMT2B-Mediated Epigenetic Modification. *PLoS One*. 11, e0166090. DOI: 10.1371/journal.pone.0166090

Sun, L., Yuan, Y., Chen, J., Ma, C. and Xu, Y. (2019). Brahma related gene 1 (BRG1) regulates breast cancer cell migration and invasion by activating MUC1 transcription. *Biochem Biophys Res Commun*. 511, 536-543. DOI: 10.1016/j.bbrc.2019.02.088

Svotelis, A., Bianco, S., Madore, J., Huppé, G., Nordell-Markovits, A., Mes-Masson, A. M., et al. (2011). H3K27 demethylation by JMJD3 at a poised enhancer of anti-apoptotic gene BCL2 determines ERα ligand dependency. *Embo j*. 30, 3947-3961. DOI: 10.1038/emboj.2011.284

Tajima, K., Matsuda, S., Yae, T., Drapkin, B. J., Morris, R., Boukhali, M., et al. (2019). SETD1A protects from senescence through regulation of the mitotic gene expression program. *Nat Commun*. 10, 2854. DOI: 10.1038/s41467-019-10786-w

Tang, X., Li, G., Su, F., Cai, Y., Shi, L., Meng, Y., et al. (2020). HDAC8 cooperates with SMAD3/4 complex to suppress SIRT7 and promote cell survival and migration. *Nucleic Acids Res*. 48, 2912-2923. DOI: 10.1093/nar/gkaa039

Taniguchi, H., Jacinto, F. V., Villanueva, A., Fernandez, A. F., Yamamoto, H., Carmona, F. J., et al. (2012). Silencing of Kruppel-like factor 2 by the histone methyltransferase EZH2 in human cancer. *Oncogene*. 31, 1988-1994. DOI: 10.1038/onc.2011.387

Toth, M., Boros, I. M. and Balint, E. (2012). Elevated level of lysine 9-acetylated histone H3 at the MDR1 promoter in multidrug-resistant cells. *Cancer Sci*. 103, 659-669. DOI: 10.1111/j.1349-7006.2012.02215.x

Truax, A. D., Thakkar, M. and Greer, S. F. (2012). Dysregulated recruitment of the histone methyltransferase EZH2 to the class II transactivator (CIITA) promoter IV in breast cancer cells. *PLoS One*. 7, e36013. DOI: 10.1371/journal.pone.0036013

Tu, W. B., Shiah, Y. J., Lourenco, C., Mullen, P. J., Dingar, D., Redel, C., et al. (2018). MYC Interacts with the G9a Histone Methyltransferase to Drive Transcriptional Repression and Tumorigenesis. *Cancer Cell*. 34, 579-595.e578. DOI: 10.1016/j.ccell.2018.09.001

van den Beucken, T., Koch, E., Chu, K., Rupaimoole, R., Prickaerts, P., Adriaens, M., et al. (2014). Hypoxia promotes stem cell phenotypes and poor prognosis through epigenetic regulation of DICER. *Nat Commun*. 5, 5203. DOI: 10.1038/ncomms6203

Viré, E., Curtis, C., Davalos, V., Git, A., Robson, S., Villanueva, A., et al. (2014). The breast cancer oncogene EMSY represses transcription of antimetastatic microRNA miR-31. *Mol Cell*. 53, 806-818. DOI: 10.1016/j.molcel.2014.01.029

Wade, M. A., Jones, D., Wilson, L., Stockley, J., Coffey, K., Robson, C. N., et al. (2015). The histone demethylase enzyme KDM3A is a key estrogen receptor regulator in breast cancer. *Nucleic Acids Res*. 43, 196-207. DOI: 10.1093/nar/gku1298

Wang, J., Duan, Z., Nugent, Z., Zou, J. X., Borowsky, A. D., Zhang, Y., et al. (2016). Reprogramming metabolism by histone methyltransferase NSD2 drives endocrine resistance via coordinated activation of pentose phosphate pathway enzymes. *Cancer Lett*. 378, 69-79. DOI: 10.1016/j.canlet.2016.05.004

Wang, J. J., Zou, J. X., Wang, H., Duan, Z. J., Wang, H. B., Chen, P., et al. (2019). Histone methyltransferase NSD2 mediates the survival and invasion of triple-negative breast cancer cells via stimulating ADAM9-EGFR-AKT signaling. *Acta Pharmacol Sin*. 40, 1067-1075. DOI: 10.1038/s41401-018-0199-z

Wang, Q., Ma, S., Song, N., Li, X., Liu, L., Yang, S., et al. (2016). Stabilization of histone demethylase PHF8 by USP7 promotes breast carcinogenesis. *J Clin Invest*. 126, 2205-2220. DOI: 10.1172/jci85747

Wang, Q., Wei, J., Su, P. and Gao, P. (2015). Histone demethylase JARID1C promotes breast cancer metastasis cells via down regulating BRMS1 expression. *Biochem Biophys Res Commun*. 464, 659-666. DOI: 10.1016/j.bbrc.2015.07.049

Wang, R. H., Zheng, Y., Kim, H. S., Xu, X., Cao, L., Luhasen, T., et al. (2008). Interplay among BRCA1, SIRT1, and Survivin during BRCA1-associated tumorigenesis. *Mol Cell*. 32, 11-20. DOI: 10.1016/j.molcel.2008.09.011

Wang, T., Mao, B., Cheng, C., Zou, Z., Gao, J., Yang, Y., et al. (2018). YAP promotes breast cancer metastasis by repressing growth differentiation factor-15. *Biochim Biophys Acta Mol Basis Dis*. 1864, 1744-1753. DOI: 10.1016/j.bbadis.2018.02.020

Wang, W., Lim, K. G., Feng, M., Bao, Y., Lee, P. L., Cai, Y., et al. (2018). KDM6B Counteracts EZH2-Mediated Suppression of IGFBP5 to Confer Resistance to PI3K/AKT Inhibitor Treatment in Breast Cancer. *Mol Cancer Ther*. 17, 1973-1983. DOI: 10.1158/1535-7163.Mct-17-0802

Wang, Y., Zhang, J., Wu, L., Liu, W., Wei, G., Gong, X., et al. (2018). Tricho-rhino-phalangeal syndrome 1 protein functions as a scaffold required for ubiquitin-specific protease 4-directed histone deacetylase 2 de-ubiquitination and tumor growth. *Breast Cancer Res*. 20, 83. DOI: 10.1186/s13058-018-1018-7

Wang, Y. F., Zhang, J., Su, Y., Shen, Y. Y., Jiang, D. X., Hou, Y. Y., et al. (2017). G9a regulates breast cancer growth by modulating iron homeostasis through the repression of ferroxidase hephaestin. *Nat Commun*. 8, 274. DOI: 10.1038/s41467-017-00350-9

Wang, Z., Kong, J., Wu, Y., Zhang, J., Wang, T., Li, N., et al. (2018). PRMT5 determines the sensitivity to chemotherapeutics by governing stemness in breast cancer. *Breast Cancer Res Treat*. 168, 531-542. DOI: 10.1007/s10549-017-4597-6

Wong, P. P., Miranda, F., Chan, K. V., Berlato, C., Hurst, H. C. and Scibetta, A. G. (2012). Histone demethylase KDM5B collaborates with TFAP2C and Myc to repress the cell cycle inhibitor p21(cip) (CDKN1A). *Mol Cell Biol*. 32, 1633-1644. DOI: 10.1128/mcb.06373-11

Wu, K., Li, Z., Cai, S., Tian, L., Chen, K., Wang, J., et al. (2013). EYA1 phosphatase function is essential to drive breast cancer cell proliferation through cyclin D1. *Cancer Res*. 73, 4488-4499. DOI: 10.1158/0008-5472.Can-12-4078

Wu, L., Cao, J., Cai, W. L., Lang, S. M., Horton, J. R., Jansen, D. J., et al. (2018). KDM5 histone demethylases repress immune response via suppression of STING. *PLoS Biol*. 16, e2006134. DOI: 10.1371/journal.pbio.2006134

Wu, Y., Shi, W., Tang, T., Wang, Y., Yin, X., Chen, Y., et al. (2019). miR-29a contributes to breast cancer cells epithelial-mesenchymal transition, migration, and invasion via down-regulating histone H4K20 trimethylation through directly targeting SUV420H2. *Cell Death Dis*. 10, 176. DOI: 10.1038/s41419-019-1437-0

Wu, Y., Wang, Y., Yang, X. H., Kang, T., Zhao, Y., Wang, C., et al. (2013). The deubiquitinase USP28 stabilizes LSD1 and confers stem-cell-like traits to breast cancer cells. *Cell Rep*. 5, 224-236. DOI: 10.1016/j.celrep.2013.08.030

Xiang, Y., Yan, K., Zheng, Q., Ke, H., Cheng, J., Xiong, W., et al. (2019). Histone Demethylase KDM4B Promotes DNA Damage by Activating Long Interspersed Nuclear Element-1. *Cancer Res*. 79, 86-98. DOI: 10.1158/0008-5472.Can-18-1310

Xie, G., Liu, X., Zhang, Y., Li, W., Liu, S., Chen, Z., et al. (2017). UTX promotes hormonally responsive breast carcinogenesis through feed-forward transcription regulation with estrogen receptor. *Oncogene*. 36, 5497-5511. DOI: 10.1038/onc.2017.157

Yamane, K., Tateishi, K., Klose, R. J., Fang, J., Fabrizio, L. A., Erdjument-Bromage, H., et al. (2007). PLU-1 is an H3K4 demethylase involved in transcriptional repression and breast cancer cell proliferation. *Mol Cell*. 25, 801-812. DOI: 10.1016/j.molcel.2007.03.001

Yang, F., Sun, L., Li, Q., Han, X., Lei, L., Zhang, H., et al. (2012). SET8 promotes epithelial-mesenchymal transition and confers TWIST dual transcriptional activities. *Embo j*. 31, 110-123. DOI: 10.1038/emboj.2011.364

Yang, J., Jubb, A. M., Pike, L., Buffa, F. M., Turley, H., Baban, D., et al. (2010). The histone demethylase JMJD2B is regulated by estrogen receptor alpha and hypoxia, and is a key mediator of estrogen induced growth. *Cancer Res*. 70, 6456-6466. DOI: 10.1158/0008-5472.Can-10-0413

Yao, R., Jiang, H., Ma, Y., Wang, L., Wang, L., Du, J., et al. (2014). PRMT7 induces epithelial-to-mesenchymal transition and promotes metastasis in breast cancer. *Cancer Res*. 74, 5656-5667. DOI: 10.1158/0008-5472.Can-14-0800

Yeh, I. J., Esakov, E., Lathia, J. D., Miyagi, M., Reizes, O. and Montano, M. M. (2019). Phosphorylation of the histone demethylase KDM5B and regulation of the phenotype of triple negative breast cancer. *Sci Rep*. 9, 17663. DOI: 10.1038/s41598-019-54184-0

Yomtoubian, S., Lee, S. B., Verma, A., Izzo, F., Markowitz, G., Choi, H., et al. (2020). Inhibition of EZH2 Catalytic Activity Selectively Targets a Metastatic Subpopulation in Triple-Negative Breast Cancer. *Cell Rep*. 30, 755-770.e756. DOI: 10.1016/j.celrep.2019.12.056

Yu, C. W., Cheng, K. C., Chen, L. C., Lin, M. X., Chang, Y. C. and Hwang-Verslues, W. W. (2018). Pro-inflammatory cytokines IL-6 and CCL2 suppress expression of circadian gene Period2 in mammary epithelial cells. *Biochim Biophys Acta Gene Regul Mech*. 1861, 1007-1017. DOI: 10.1016/j.bbagrm.2018.09.003

Yu, L., Di, Y., Xin, L., Ren, Y., Liu, X., Sun, X., et al. (2017). SND1 acts as a novel gene transcription activator recognizing the conserved Motif domains of Smad promoters, inducing TGFβ1 response and breast cancer metastasis. *Oncogene*. 36, 3903-3914. DOI: 10.1038/onc.2017.30

Yu, L., Liang, Y., Cao, X., Wang, X., Gao, H., Lin, S. Y., et al. (2017). Identification of MYST3 as a novel epigenetic activator of ERα frequently amplified in breast cancer. *Oncogene*. 36, 2910-2918. DOI: 10.1038/onc.2016.433

Yu, S., Gong, X., Ma, Z., Zhang, M., Huang, L., Zhang, J., et al. (2020). Endocrine resistant breast cancer cells with loss of ERα expression retain proliferative ability by reducing caspase7-mediated HDAC3 cleavage. *Cell Oncol (Dordr)*. 43, 65-80. DOI: 10.1007/s13402-019-00439-x

Yu, S. L., Lee, D. C., Son, J. W., Park, C. G., Lee, H. Y. and Kang, J. (2013). Histone deacetylase 4 mediates SMAD family member 4 deacetylation and induces 5-fluorouracil resistance in breast cancer cells. *Oncol Rep*. 30, 1293-1300. DOI: 10.3892/or.2013.2578

Yu, W., Huang, W., Yang, Y., Qiu, R., Zeng, Y., Hou, Y., et al. (2019). GATA3 recruits UTX for gene transcriptional activation to suppress metastasis of breast cancer. *Cell Death Dis*. 10, 832. DOI: 10.1038/s41419-019-2062-7

Yu, Y., Qi, J., Xiong, J., Jiang, L., Cui, D., He, J., et al. (2019). Epigenetic Co-Deregulation of EZH2/TET1 is a Senescence-Countering, Actionable Vulnerability in Triple-Negative Breast Cancer. *Theranostics*. 9, 761-777. DOI: 10.7150/thno.29520

Zeidler, M., Varambally, S., Cao, Q., Chinnaiyan, A. M., Ferguson, D. O., Merajver, S. D., et al. (2005). The Polycomb group protein EZH2 impairs DNA repair in breast epithelial cells. *Neoplasia*. 7, 1011-1019. DOI: 10.1593/neo.05472

Zeng, Y., Qiu, R., Yang, Y., Gao, T., Zheng, Y., Huang, W., et al. (2019). Regulation of EZH2 by SMYD2-Mediated Lysine Methylation Is Implicated in Tumorigenesis. *Cell Rep*. 29, 1482-1498.e1484. DOI: 10.1016/j.celrep.2019.10.004

Zhang, H., Zhang, Y., Chen, C., Zhu, X., Zhang, C., Xia, Y., et al. (2018). A double-negative feedback loop between DEAD-box protein DDX21 and Snail regulates epithelial-mesenchymal transition and metastasis in breast cancer. *Cancer Lett*. 437, 67-78. DOI: 10.1016/j.canlet.2018.08.021

Zhang, J., Sui, S., Wu, H., Zhang, J., Zhang, X., Xu, S., et al. (2019). The transcriptional landscape of lncRNAs reveals the oncogenic function of LINC00511 in ER-negative breast cancer. *Cell Death Dis*. 10, 599. DOI: 10.1038/s41419-019-1835-3

Zhang, K. J., Tan, X. L. and Guo, L. (2020). The long non-coding RNA DANCR regulates the inflammatory phenotype of breast cancer cells and promotes breast cancer progression via EZH2-dependent suppression of SOCS3 transcription. *Mol Oncol*. 14, 309-328. DOI: 10.1002/1878-0261.12622

Zhang, R., Li, X., Liu, Z., Wang, Y., Zhang, H. and Xu, H. (2020). EZH2 inhibitors-mediated epigenetic reactivation of FOSB inhibits triple-negative breast cancer progress. *Cancer Cell Int*. 20, 175. DOI: 10.1186/s12935-020-01260-5

Zhang, Y., Liu, J., Lin, J., Zhou, L., Song, Y., Wei, B., et al. (2016). The transcription factor GATA1 and the histone methyltransferase SET7 interact to promote VEGF-mediated angiogenesis and tumor growth and predict clinical outcome of breast cancer. *Oncotarget*. 7, 9859-9875. DOI: 10.18632/oncotarget.7126

Zhao, Q. Y., Lei, P. J., Zhang, X., Zheng, J. Y., Wang, H. Y., Zhao, J., et al. (2016). Global histone modification profiling reveals the epigenomic dynamics during malignant transformation in a four-stage breast cancer model. *Clin Epigenetics*. 8, 34. DOI: 10.1186/s13148-016-0201-x

Zhao, Y., He, J., Yang, L., Luo, Q. and Liu, Z. (2018). Histone Deacetylase-3 Modification of MicroRNA-31 Promotes Cell Proliferation and Aerobic Glycolysis in Breast Cancer and Is Predictive of Poor Prognosis. *J Breast Cancer*. 21, 112-123. DOI: 10.4048/jbc.2018.21.2.112

Zheng, Q., Fan, H., Meng, Z., Yuan, L., Liu, C., Peng, Y., et al. (2018). Histone demethylase KDM2B promotes triple negative breast cancer proliferation by suppressing p15INK4B, p16INK4A, and p57KIP2 transcription. *Acta Biochim Biophys Sin (Shanghai)*. 50, 897-904. DOI: 10.1093/abbs/gmy084

Zheng, Y., Zeng, Y., Qiu, R., Liu, R., Huang, W., Hou, Y., et al. (2018). The Homeotic Protein SIX3 Suppresses Carcinogenesis and Metastasis through Recruiting the LSD1/NuRD(MTA3) Complex. *Theranostics*. 8, 972-989. DOI: 10.7150/thno.22328
